# Four New Pairs of MetO-Containing Diketopiperazine Enantiomers: Isolation, Synthesis and Potential Anti-Parkinson’s Disease Activity

**DOI:** 10.3390/md23120477

**Published:** 2025-12-13

**Authors:** Yu Lei, Zhenyu Yang, Daichun Li, Xiaojian Liao, Chamari Hettiarachchi, Bingxin Zhao, Shihai Xu

**Affiliations:** 1 Department of Chemistry, College of Chemistry and Materials Science, Jinan University, Guangzhou 510632, China; 31000652@scau.edu.cn (Y.L.); aries@stu.jnu.edu.cn (Z.Y.); ldcman123456789@163.com (D.L.); tliaoxj@jnu.edu.cn (X.L.); 2 Key Laboratory for Biobased Materials and Energy of Ministry of Education, College of Materials and Energy, South China Agricultural University, Guangzhou 510642, China; 3 Department of Chemistry, University of Colombo, Colombo 00700, Sri Lanka

**Keywords:** marine sponge, *Dysidea*, diketopiperazine, methionine sulfoxide, supercritical fluid chromatography, total synthesis

## Abstract

Four new methionine sulfoxide-containing diketopiperazines, (+)-dysidmetsulfoxide A [(+)-**1**], (+)-dysidmetsulfoxide B [(+)-**2**], (+)-dysidmetsulfoxide C [(+)-**3**] and (−)-dysidmetsulfoxide C [(−)-**3**], were isolated from the South China Sea sponge *Dysidea* sp. These compounds represented the first example of diketopiperazines possessing the unit of methionine sulfoxide (MetO) isolated from marine sponges. As it was difficult to determine the configuration of chiral sulfur atom in the thionyl group, the structures with absolute configurations of these compounds were elucidated by spectroscopic analyses and total synthesis. It was noteworthy that the purchased synthetic precursors, Fmoc-*L*- and Fmoc-*D*-MetO, were mixtures of epimers, respectively, due to the stereogenic sulfur atom in MetO, which were separated to prepare the optically pure isomers via the method of supercritical fluid chromatography (SFC). In addition, the other four optical isomers [(−)-**1**, (−)-**2**, (+)-**4** and (−)-**4**] were also synthesized. Furthermore, (+)-**1**, (−)-**1**, (+)-**3**, (+)-**4** and (−)-**4** showed potential anti-Parkinson’s disease activities in an in vivo zebrafish model.

## 1. Introduction

The large number of sulfates in the ocean provide rich sulfur sources for marine organisms, which promote the synthesis of sulfur-containing amino acids required for peptides or proteins in the biosynthetic pathway [1,2]. Methionine sulfoxide (MetO) is a class of sulfur-containing amino acid derivative, in which the sulfur atom is oxidized to a thionyl group. So far, only 16 cyclopeptides and polypeptides containing MetO have been isolated and characterized from marine sponges, which exist as diastereomeric mixtures or with unascertained configuration at the MetO unit because of the chiral sulfur atom in the thionyl group [3,4,5,6,7,8,9,10]. Notably, no MetO-containing diketopiperazine has been reported from marine sponges. It is difficult to separate such diastereoisomers as their physicochemical properties are very similar. Currently, HPLC, GC, capillary electrophoresis (CE) and supercritical fluid chromatography (SFC) are used to purify these stereoisomers. Remarkably, the method of SFC represented a significant advance on the gramme-level quantities and high purities compared with other chiral resolution methods. However, not all of these compounds can be separated via these methods [11,12,13]. In addition, the sulfoxides with the same planar structures but different configurations at sulfur atom might show different biological activities. For example, axinellasins A and D, which are two optically pure MetO-containing cycloheptapeptides originally from the marine sponge *Axinella* sp., exhibited better immunosuppressive effects than other related diastereomers [4]. Thus, it is significant to separate the chiral sulfoxide isomers and determine their absolute configurations.

The marine sponge of genus *Dysidea* (family Dysideidae, order Dictyoceratida) is widely distributed in the South China Sea, which is a prolific source of structurally diverse secondary metabolites, including terpenoids, alkaloids and sterols [14,15,16]. These compounds exhibit a broad spectrum of bioactivities, such as cytotoxicity, antioxidant, antibacterial and anti-HIV properties [17,18].

**Figure 1:**
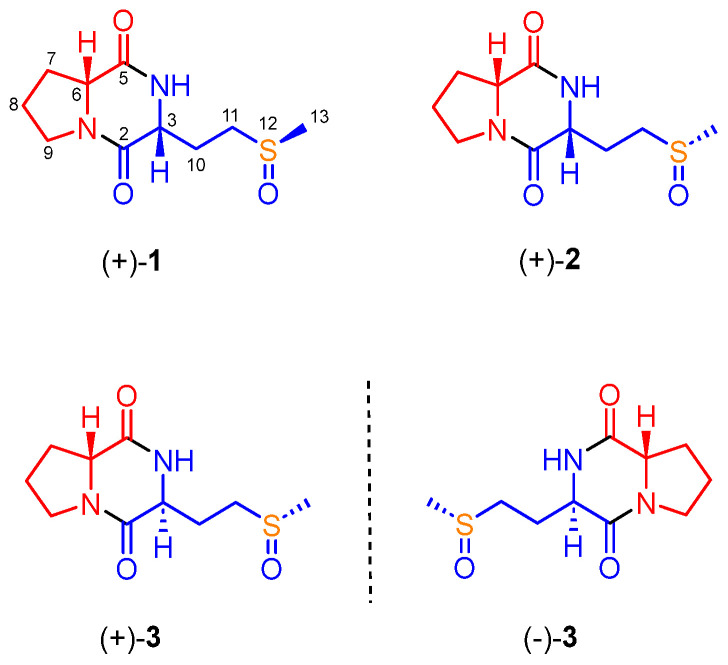
Chemical structures of (+)-**1**, (+)-**2**, (+)-**3** and (−)-**3**.

In this paper, four new MetO-containing optically pure diketopiperazines, (+)-dysidmetsulfoxide A [(+)-**1**], (+)-dysidmetsulfoxide B [(+)-**2**], (+)-dysidmetsulfoxide C [(+)-**3**] and (−)-dysidmetsulfoxide C [(−)-**3**], were isolated from the sponge *Dysidea* sp. (Figure 1). Their structures with absolute configurations were elucidated via extensive spectroscopic analysis and synthesis. Furthermore, another four optical isomers of these compounds [(−)-**1**, (−)-**2**, (+)-**4** and (−)-**4**] were also synthesized. These compounds were the first example of diketopiperazines containing MetO from marine sponges. Herein, we report the discovery, diastereomeric separation, structure elucidation, synthesis and activity investigation of **1**–**4**.

## 2. Results

(+)-Dysidmetsulfoxide A [(+)-**1**] was obtained as a white amorphous solid with [*α*${]}_{D}^{25}$ +6.4 (*c* 0.25, CH_3_OH). The molecular formula of (+)-**1** was confirmed to be C_10_H_16_N_2_O_3_S by HR-ESI-MS at *m*/*z* 245.0954 [M + H]^+^ (calcd for C_10_H_17_N_2_O_3_S: 245.0954), indicating 5 degrees of unsaturation. The IR bands implied the presence of amino (3329 cm^−1^), carbonyl (1652 cm^−1^) and sulfoxide (1019 cm^−1^) groups [14]. The ^1^H and ^13^C NMR data of (+)-**1** revealed the presence of 10 carbon signals, including two carbonyls [*δ*_C_ 172.7 (C-5) and 167.2 (C-2)], two methine [*δ*_H_ 4.36 (1H, t, *J* = 4.9 Hz, H-3) and 4.26 (1H, t, *J* = 7.9 Hz, H-6); *δ*_C_ 55.2 (C-3) and 60.4 (C-6)], five methylenes [*δ*_H_ 3.57~3.48 (2H, m, H-9), 3.00~2.26 (2H, m, H-11), 2.31 (1H, m, H-7a), 2.30~2.26 (2H, m, H-10), 2.03 (1H, m, H-7b) and 2.01~1.98 (2H, m, H-8); *δ*_C_ 50.0 (C-11), 46.4 (C-9), 29.2 (C-7), 23.9 (C-10) and 23.5 (C-8)] and one methyl [*δ*_H_ 2.66 (s, 3H, H-13); *δ*_C_ 38.2 (C-13)]. The above data (Table·1) were highly similar to those of cyelo(*L*-Pro-*L*-MetO), which was isolated from *Streptomyces* sp. YIM 63342 [19,20]. However, the absolute configuration of the sulfoxide sulfur remains to be established.

**Figure 2:**
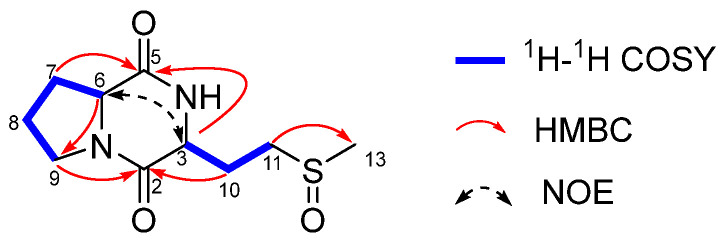
Key ^1^H-^1^H COSY, HMBC and NOE correlations of (+)-**1**.

Further analysis of 2D NMR data permitted the construction of the planar structure of diketopiperazine ring in (+)-**1**. In the ^1^H-^1^H COSY spectrum, two spin coupling systems were observed, which are drawn in bold (Figure 2). In the HMBC spectrum, the corrections from H-3 to C-5, from H-6 to C-9, from H-7b to C-5, from H-9/H-10 to C-2 as well as from H-11 to C-13 were observed, which determined the planar structure of the diketopiperazine ring in (+)-**1**. The partial relative configuration of (+)-**1** was determined via the NOESY spectrum. The presence of NOE correlation between H-3 to H-6 revealed that they were in the same orientation (Figure 2). However, the stereochemistry of the sulfur atom could not be elucidated via the NOESY spectrum. Additionally, the structure of (+)-**1** with absolute configuration was further confirmed by the method of synthesis, which will be described in the latter part of this article.

(+)-Dysidmetsulfoxide B [(+)-**2**] was also isolated as a white amorphous solid with [*α*${]}_{D}^{25}$ +6.1 (*c* 0.25, CH_3_OH), the molecular formula of which was determined as the same as (+)-**1**. The UV, IR and NMR data of (+)-**2** (Table 1) revealed high structural similarities between (+)-**1** and (+)-**2**. The only slight differences between them were at C-11 and C-13. The whole planar structure of the diketopiperazine ring in (+)-**2** was further confirmed by the analysis of 2D data. Furthermore, the relative configuration of (+)-**2** was partially determined according to the NOE correlation between H-3 and H-6 in the NOESY spectrum, which suggested that they had the same orientation (Figure 2). Thus, (+)-**2** might be the S-12 epimer of (+)-**1**. The structure of (+)-**2** with absolute configuration was further confirmed by the method of synthesis, which will be described in the latter part of this article too.

Dysidmetsulfoxide C (**3**) was obtained as a white amorphous solid, which showed the same molecular formula as (+)-**1**. The 1D NMR data of **3** (Table 1) resembled those of (+)-**1** except C-3, C-5, C-6 and C-10. The planar structure of the diketopiperazine ring in **3** was further confirmed by the ^1^H-^1^H COSY and HMBC experiments, which were the same as (+)-**1**. The relative configuration of **3** could not be determined due to the absence of key correlations in the NOESY spectrum. Meanwhile, the optical rotation of **3** was close to zero, which suggested that it was a racemic mixture. Therefore, chiral HPLC was used to separate this mixture. Eventually, compounds (+)-**3** {[*α*${]}_{D}^{25}$ +6.2 (*c* 0.25, CH_3_OH)} and (−)-**3** {[*α*${]}_{D}^{25}$ −6.4 (*c* 0.25, CH_3_OH)} were successfully obtained (Figure S1). Both of the absolute configurations of (+)-**3** and (−)-**3** were determined via the method of synthesis, which will be described in the latter part of this article too.

In order to determine the absolute configurations of these four compounds, the method of synthesis was conducted. The synthetic precursors were proline and MetO. Thus, we purchased *D*- and *L*-proline methyl esters as well as Fmoc-*L*- and Fmoc-*D*-MetO. It was worth noting that the purchased Fmoc-*L*- and Fmoc-*D*-MetO were each mixture of diastereomers due to the stereogenic sulfur atom in MetO. Therefore, the chiral isolation of Fmoc-*L*- and Fmoc-*D*-MetO was necessary to perform first. However, after attempting chiral HPLC with different chiral columns, we were unable to separate them. Ultimately, four optically pure isomers, (*S*)- and (*R*)-Fmoc-*L*-MetO as well as (*S*)- and (*R*)-Fmoc-*D*-MetO, were successfully obtained via SFC separation strategy with a Chiralpak IC-3 column. This approach enabled highly efficient preparation of the diastereomers, overcoming the limitations associated with the conventional HPLC-based purification method (Figure S2). The absolute configurations of (*S*)- and (*R*)-Fmoc-*L*-MetO were confirmed by comparing their NMR data with those reported in the literature [4], while the structures of (*S*)- and (*R*)-Fmoc-*D*-MetO were unambiguously determined through the methods of spectroscopic analyses and single crystal X-ray diffraction.

**Scheme 1:**
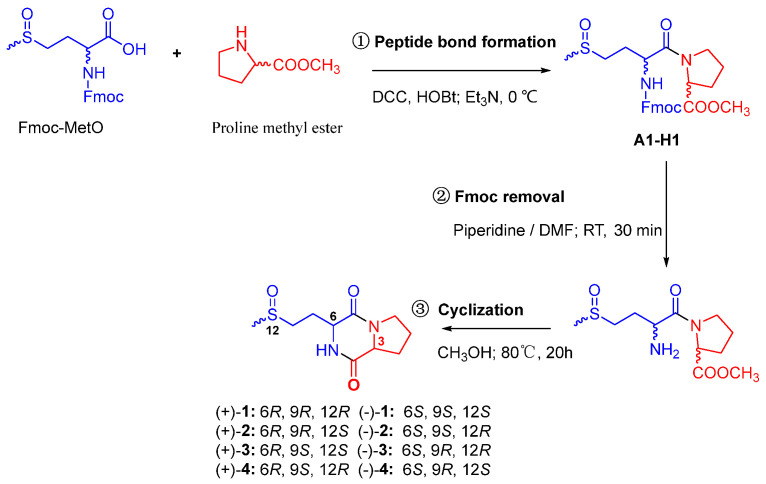
Synthetic route of **1**–**4**.

Then, the total synthesis of dysidmetsulfoxides were carried out (Scheme 1). Firstly, the *D*- and *L*-proline methyl esters were reacted with four prepared optically pure Fmoc-MetO [(*S*)- and (*R*)-Fmoc-*L*-MetO as well as (*S*)- and (*R*)-Fmoc-*D*-MetO] in the presence of dicyclohexylcarbodiimide (DCC), and triethylamine (Et_3_N) to afford **A1**–**H1**, respectively [21]. The isolated yields were approximately 86.0%. Subsequently, the use of 20% piperidine in DMF solution deprotected the *N*-terminal Fmoc groups [22]; the reaction proceeded cleanly and quantitatively (yield > 95%), thus the crude mixture was used directly in the next step without further purification. Finally, polar solvent MeOH was used to achieve a head to tail cyclization for delivering eight isomers at 80 °C, with isolated yields ranging from 26.1% to 39.1% [23]. (+)- and (−)-dysidmetsulfoxides A–D (**1**–**4**), which were then fully characterized.

The MS, NMR and optical rotations of natural (+)-**1**, (+)-**2**, (+)-**3** and (−)-**3** were compared with those of synthesized ones. As shown in Supporting Information, the spectroscopic data and specific optical rotation of natural (+)-**1**, (+)-**2**, (+)-**3**, (−)-**3** were in good agreement with those of the synthesized (+)-(3*R*, 6*R*, 12*R*)-**1**, (+)-(3*R*, 6*R*, 12*S*)-**2**, (+)-(3*R*, 6*S*, 12*S*)-**3**, (−)-(3*S*, 6*R*, 12*R*)-**3**, respectively. Thus, the structures with absolute configurations of (+)-**1**, (+)-**2**, (+)-**3** and (−)-**3** were determined.

**Figure 3:**
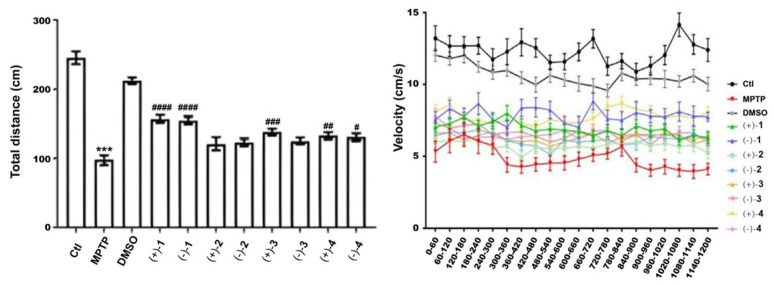
The swimming speed and distance of zebrafish in the 48-well plate (compared to the blank control group: *** *p* < 0.001; compared to the model group: ^####^*p* < 0.0001, ^###^ *p* < 0.001, ^##^ *p* < 0.01, ^#^ *p* < 0.02).

Our group has been committed to the study of neuroactivity. The previous literature reported that some diketopiperazines exhibited neuroprotective effects and anti-Parkinson’s disease (PD) activities [24]. Thus, all these synthesized compounds were evaluated for anti-Parkinson’s disease activities in an in vivo zebrafish model (Figure 3). Compounds (+)-**1**, (−)-**1**, (+)-**3**, (+)-**4** and (−)-**4** significantly enhanced both the average movement velocity and total travel distance of 1-methyl-4-phenyl-1,2,3,6-tetrahydropyridine (MPTP)-treated zebrafish with statistically significant differences observed. Specifically, (+)-**1** and (−)-**1** exhibited the most pronounced efficacy in ameliorating MPTP-induced motor deficits (*p* < 0.0001). Therefore, these compounds will be further studied and expected to become potential anti-Parkinson’s drugs.

## 3. Materials and Methods

### 3.1. General Experimental Procedures

UV spectra were measured via a Shimadzu UV-2401PC spectrometer (Shimadzu, Kyoto, Japan). IR spectra were taken on Thermo Nicolet iS50 FT-IR (Thermo Fisher Scientific, Waltham, MA USA). Optical rotations were determined with a P-2000 Digital Polarimeter (JASCO International Co., Ltd., Hachioji, Japan). NMR experiments were performed on Bruker Av 600 NMR (Bruker, Fällanden, Switzerland). HR-ESIMS spectra were acquired using an Agilent 6210 LC-ESI-Q/TOF mass spectrometer (Agilent Technologies Inc., Santa Clara, CA, USA). HPLC separation was carried on Agilent 1260 series apparatus (Agilent, Palo Alto, California, USA), equipped with YMC-Pack ODS-A (20 × 250 mm, 5.0 μm) and Phenomenox Lux^®^5 μm Amylose-1 (4.6 × 250 mm, 5 μm). SFC purifications were carried out on a Waters SFC Prep 15 system with SFC IC-3 (Daicel Corporation, Osaka Prefecture, Japan, Chiralpak, 10 × 250 mm, 5.0 μm) column. Column chromatography was performed with silica gel (300–400 mesh, Qingdao Marine, Chemical Co., Qingdao, China) and Sephadex LH-20 (GE Healthcare, Uppsala, Sweden). The Thin-Layer Chromatography silica gel plate (0.2 ± 0.03 mm, HSGH 254) was purchased from the Qingdao Marine Chemical Factory, Qingdao, China. All amino acids (Fmoc-*L*-MetO, Fmoc-*D*-MetO, *L*-Pro-OMe, *D*-Pro-OMe) were purchased from Sigma-Aldrich Chemical Corporation.

### 3.2. Animal Material

The sponge *Dysidea* sp. was collected from Xuwen County, Zhanjiang City, Guangdong Province, China, in May 2017, which was identified by Professor De-Xiang Wang. The specimen (May 2017) has been deposited in the Department of Chemistry, College of Chemistry and Materials Science, Jinan University.

### 3.3. Extraction and Isolation

The sponge *Dysidea* sp. (20.0 Kg, wet weight) was powdered and extracted with 95% EtOH four times (each time, 5 L, 3 days) at room temperature to obtain 1.8 Kg extract, which was suspended in 2 L H_2_O and partitioned by petroleum ether, EtOAc and *n*-butanol for five times, respectively. The *n*-BuOH extract (180 g) was applied to silica gel column chromatography with gradient elution using CH_2_Cl_2_/CH_3_OH (100:0–0:100) to yield six fractions (Frs. 1–6). Fr. 5 (1.2 g) was subjected to Sephadex LH-20 column with CH_2_Cl_2_/CH_3_OH (1:1) to yield 5 fractions (Frs. 5–1~5–5). Fr. 5–3 (15.5 mg) was purified by preparative reversed-phase HPLC (YMC-Pack ODS-A, CH_3_OH /H_2_O = 10/90, flow rate = 8 mL/min) to afford (+)-**1** (0.6 mg, t*_R_* = 22.3 min), (+)-**2** (0.7 mg, t*_R_* = 25.5 min), **3** (0.7 mg, t*_R_* = 28.7 min). Compound **3** was further purified by chiral column (Lux^®^5 μm Amylose-1, 5 μm, 4.6 × 250 mm, CH_3_OH, flow rate = 0.6 mL/min) to yield (+)-**3** (0.3 mg, t*_R_* = 6.7 min) and (−)-**3** (0.3 mg, t*_R_* = 7.7 min). Quality ratio of (+)-**3** and (−)-**3** were approximately 1:1.

Natural (+)-dysidmethsulfoxide A [(+)-**1**]: white amorphous solid; [*α*${]}_{D}^{25}$ +6.4 (*c* 0.25, CH_3_OH); mp 142–143 °C; UV (CH_3_OH) *λ*_max_: 224 nm; IR (KBr) *ν*_max_: 3329, 2944, 2833, 1652, 1452, 1019, 616 cm^−1^; HR-ESI-MS: 245.0954 [M + H]^+^ (calcd for C_10_H_17_N_2_O_3_S: 245.0954); ^1^H NMR and ^13^C NMR data, see Table 1.

Natural (+)-dysidmethsulfoxide B [(+)-**2**]: white amorphous solid; [*α*${]}_{D}^{25}$ +6.1 (*c* 0.25, CH_3_OH); mp 147–148 °C; UV (CH_3_OH) *λ*_max_: 224 nm; IR (KBr) *ν*_max_: 3352, 2927, 1646, 1430, 1299, 1182, 1126, 1014, 947 cm^−1^; HR-ESI-MS: 267.0762 [M + Na]^+^ (calcd for C_10_H_16_N_2_O_3_SNa: 267.0774); ^1^H NMR and ^13^C NMR data, see Table 1.

Natural dysidmethsulfoxide C (**3**): white amorphous solid; [*α*${]}_{D}^{25}$ +0.23 (*c* 0.25, CH_3_OH); mp 140–141 °C; UV (CH_3_OH) *λ*_max_: 224 nm; IR (KBr) *ν*_max_: 3380, 1645, 1449, 1297, 1159, 1119, 1007, 945 cm^−1^; HR-ESI-MS: 267.0766 [M + Na]^+^ (calcd for C_10_H_16_N_2_O_3_SNa: 267.0774); ^1^H NMR and ^13^C NMR data, see Table 1.

(+)-**3**: [*α*${]}_{D}^{25}$ +6.2 (*c* 0.25, CH_3_OH);

(−)-**3**: [*α*${]}_{D}^{25}$ −6.4 (*c* 0.25, CH_3_OH).

### 3.4. Total Synthesis of 1–4

Peptide bond formation: (*R*)-Fmoc-*D*-MetO (1.14 g, 3.0 mmol), (*S*)-Fmoc-*L*-MetO (1.14 g, 3.0 mmol), (*S*)-Fmoc-*D*-MetO (1.14 g, 3.0 mmol), (*R*)-Fmoc-*L*-MetO (1.14 g, 3.0 mmol), (*S*)-Fmoc-*L*-MetO (1.14 g, 3.0 mmol), (*R*)-Fmoc-*D*-MetO (1.14 g, 3.0 mmol), (*R*)-Fmoc-*L*-MetO (1.14 g, 3.0 mmol), (*S*)-Fmoc-*D*-MetO (1.14 g, 3.0 mmol) were placed into 8 reaction flasks (labeled Nos. 1–8) and dissolved in CH_2_Cl_2_ (10 mL), respectively. The solution was stirred at 0 °C; Et_3_N (0.30 g, 3 mmol) and DCC (0.74 g, 3.6 mmol) was then added. The mixture was stirred at 0 °C for 5 min. Then added *D*-Proline methyl esters (1.16 g, 3.0 mmol) to reaction flasks Nos. 1, 3, 5, 7 and *L*-Proline methyl esters (1.16 g, 3.0 mmol) to reaction flasks Nos. 2, 4, 6 and 8 at room temperature for 12 h, respectively. Upon stirring for 12 h, the reaction mixtures were quenched with water and extracted with CH_2_Cl_2_, then dried over Na_2_SO_4_. The residues were purified via flash chromatography (silica gel, petroleum/ethyl acetate = 95:5) to afford **A1**–**H1** (1.3 g, 86.0%) as a white solid.

Fmoc removal: The Fmoc deprotection was carried out using 20% piperidine/DMF (V_piperidine_:V_DMF_ = 1:4) at room temperature for 20 min.

Cyclization: We added 20 mL of the polar solvent CH_3_OH to each of the eight reaction bottles mentioned above. The mixtures were stirred at 80 °C for 24 h. After the reaction was confirmed via TLC monitoring, the solutions were concentrated under reduced pressure. The resulting residues were individually purified using flash chromatography (silica gel, petroleum/ethyl acetate = 90:10) to afford (+)-**1** (0.08 g, 34.7%), (−)-**1** (0.07 g, 30.4%), (+)-**2** (0.09 g, 39.1%), (−)-**2** (0.09 g, 39.1%), (+)-**3** (0.06 g, 26.1%), (−)-**3** (0.08 g, 34.7%), (+)-**4** (0.07 g, 30.4%), (−)-**4** (0.09 g, 39.1%), respectively.

(+)-dysidmethsulfoxide A [(+)-1]: white needle-like crystals (CH_3_OH): mp 142–143 °C; [*α*${]}_{D}^{25}$ +6.6 (*c* 0.25, CH_3_OH); HR-ESI-MS *m*/*z*: 245.0954 [M + H]^+^ (calcd for C_10_H_17_N_2_O_3_S: 245.0954); ^1^H NMR (600 MHz, CD_3_OD): *δ*_H_ 4.42 (1H, d, *J* = 5.4 Hz, H-3), 4.33 (1H, m, H-6), 3.63~3.52 (2H, m, H-9), 3.00 (2H, m, H-11), 2.73 (3H, s, H-13), 2.40~2.30 (3H, m, H-10, 7a), 2.12~1.93 (3H, m, H-8, 7b); ^13^C NMR (150 MHz, CD_3_OD): *δ*_C_ 172.6 (C-5), 167.1 (C-2), 60.3 (C-6), 55.1 (C-3), 49.9 (C-11), 46.4 (C-9), 38.2 (C-13), 29.2 (C-7), 23.8 (C-10), 23.5 (C-8).

(−)-dysidmethsulfoxide A [(−)-1]: white needle-like crystals (CH_3_OH): mp 142–143 °C; [*α*${]}_{D}^{25}$ −6.4 (*c* 0.25, CH_3_OH); HR-ESI-MS *m*/*z*: 267.0762 [M + Na]^+^ (calcd for C_10_H_16_N_2_O_3_SNa: 267.0774); ^1^H NMR (600 MHz, CD_3_OD): *δ*_H_ 4.42 (1H, d, *J* = 5.4 Hz, H-3), 4.33 (1H, m, H-6), 3.62~3.52 (2H, m, H-9), 3.00 (2H, m, H-11), 2.73 (3H, s, H-13), 2.40~2.29 (3H, m, H-10, 7a), 2.12–1.92 (3H, m, H-8, 7b); ^13^C NMR (150 MHz, CD_3_OD): *δ*_C_ 172.7 (C-5), 167.2 (C-2), 60.4 (C-6), 55.1 (C-3), 49.9 (C-11), 46.4 (C-9), 38.2 (C-13), 29.2 (C-7), 23.9 (C-10), 23.5 (C-8).

(+)-dysidmethsulfoxide B [(+)-2]: white solid: mp 147–148 °C; [*α*${]}_{D}^{25}$ +6.2 (*c* 0.25, CH_3_OH); HR-ESI-MS *m*/*z*: 267.0763 [M + Na]^+^ (calcd for C_10_H_16_N_2_O_3_SNa: 267.0774); ^1^H NMR (600 MHz, CD_3_OD): *δ*_H_ 4.40 (1H, d, *J* = 5.2 Hz, H-3), 4.31 (1H, m, H-6), 3.64~3.52 (2H, m, H-9), 3.07 (1H, m, H-11a), 2.92 (1H, m, H-11b), 2.71 (3H, s, H-13), 2.35 (3H, m, H-10, 7a), 2.13~1.91 (3H, m, H-10, 7b); ^13^C NMR (150 MHz, CD_3_OD): *δ*_C_ 172.7 (C-5), 167.2 (C-2), 60.4 (C-6), 55.3 (C-3), 49.8 (C-11), 46.5 (C-9), 38.0 (C-13), 29.3 (C-7), 23.8 (C-10), 23.5 (C-8).

(−)-dysidmethsulfoxide A [(−)-2]: white solid: mp 147–148 °C; [*α*${]}_{D}^{25}$ −6.1 (*c* 0.25, CH_3_OH); HR-ESI-MS *m*/*z*: 267.0777 [M + Na]^+^ (calcd for C_10_H_16_N_2_O_3_SNa: 267.0774); ^1^H NMR (600 MHz, CD_3_OD): *δ*_H_ 4.41 (1H, d, *J* = 5.1 Hz, H-3), 4.32 (1H, m, H-6), 3.65~3.52 (2H, m, H-9), 3.08 (1H, m, H-11a), 2.93 (1H, m, H-11b), 2.72 (3H, s, H-13), 2.35 (3H, m, H-10, 7a), 2.13~1.92 (3H, m, H-10, 7b); ^13^C NMR (150 MHz, CD_3_OD): *δ*_C_ 172.6 (C-5), 167.1 (C-2), 60.3 (C-6), 55.2 (C-3), 49.7 (C-11), 46.4 (C-9), 38.0 (C-13), 29.2 (C-7), 23.8 (C-10), 23.5 (C-8).

(+)-dysidmethsulfoxide C [(+)-3]: white solid: mp 143–144 °C; [*α*${]}_{D}^{25}$ +6.1 (*c* 0.25, CH_3_OH); HR-ESI-MS *m*/*z*: 267.0777 [M + Na]^+^ (calcd for C_10_H_16_N_2_O_3_SNa 267.0774); ^1^H NMR (600 MHz, CD_3_OD): *δ*_H_ 4.42 (1H, dd, *J* = 10.0, 6.5 Hz, H-6), 4.09 (1H, dd, *J* = 8.0, 6.2 Hz, H-3), 3.71 (1H, m, H-9a), 3.61 (1H, m, H-9b), 3.09 (1H, m, H-11a), 2.99 (1H, m, H-11b), 2.78 (3H, s, H-13), 2.50~2.39 (1H, m, H-7a), 2.32 (2H, m, H-10), 2.13 (1H, m, H-7b), 2.04 (2H, m, H-8); ^13^C NMR (150 MHz, CD_3_OD): *δ*_C_ 171.1 (C-5), 167.2 (C-2), 59.3 (C-6), 57.6 (C-3), 50.5 (C-11), 46.7 (C-9), 38.2 (C-13), 29.9 (C-7), 28.0 (C-10), 22.9 (C-8).

(−)-dysidmethsulfoxide C [(−)-3]: white solid: mp 143–144 °C; [*α*${]}_{D}^{25}$ −6.1 (*c* 0.25, CH_3_OH); HR-ESI-MS *m*/*z*: 267.0782 [M + Na]^+^ (calcd for C_10_H_16_N_2_O_3_SNa 267.0774); ^1^H NMR (600 MHz, CD_3_OD): *δ*_H_ 4.42 (1H, dd, *J* = 9.9, 6.4 Hz, H-6), 4.09 (1H, dd, *J* = 7.9, 6.3 Hz, H-3), 3.71 (1H, m, H-9a), 3.61 (1H, m, H-9b), 3.09 (1H, m, H-11a), 2.99 (1H, m, H-11b), 2.78 (3H, s, H-13), 2.49~2.39 (1H, m, H-7a), 2.32 (2H, m, H-10), 2.13 (1H, m, H-7b), 2.04 (2H, m, H-8); ^13^C NMR (150 MHz, CD_3_OD): *δ*_C_ 171.2 (C-5), 167.2 (C-2), 59.3 (C-6), 57.6 (C-3), 50.4 (C-11), 46.8 (C-9), 38.2 (C-13), 29.9 (C-7), 28.0 (C-10), 22.9 (C-8).

(+)-dysidmethsulfoxide D [(+)-4]: white solid: mp 141–142 °C; [*α*${]}_{D}^{25}$ +5.4 (*c* 0.25, CH_3_OH); HR-ESI-MS *m*/*z*: 267.0777 [M + Na]^+^ (calcd for C_10_H_16_N_2_O_3_SNa 267.0774); ^1^H NMR (600 MHz, CD_3_OD): *δ*_H_ 4.32 (1H, dd, *J* = 9.9, 6.5 Hz, H-6), 4.01 (1H, dd, *J* = 7.9, 6.4 Hz, H-3), 3.65~3.56 (1H, m, H-9a), 3.51 (1H, m, H-9b), 2.99 (1H, m, H-11a), 2.86 (1H, m, H-11b), 2.67 (3H, s, H-13), 2.34 (1H, m, H-7a), 2.22 (2H, m, H-10), 2.04 (1H, m, H-7b), 1.97~1.92 (2H, m, H-8); ^13^C NMR (150 MHz, CD_3_OD): *δ*_C_ 171.2 (C-5), 167.3 (C-2), 59.3 (C-6), 57.6 (C-3), 50.6 (C-11), 46.8 (C-9), 38.3 (C-13), 29.9 (C-7), 28.0 (C-10), 23.0 (C-8).

(−)-dysidmethsulfoxide D [(−)-4]: white solid: mp 141–142 °C; [*α*${]}_{D}^{25}$ −6.4 (*c* 0.25, CH_3_OH); HR-ESI-MS *m*/*z*: 267.0772 [M + Na]^+^ (calcd for C_10_H_16_N_2_O_3_SNa 267.0774); ^1^H NMR (600 MHz, CD_3_OD): *δ*_H_ 4.33 (1H, dd, *J* = 10.1, 6.4 Hz, H-6), 4.02 (1H, dd, *J* = 8.0, 6.4 Hz, H-3), 3.66~3.57 (1H, m, H-9a), 3.52 (1H, m, H-9b), 3.00 (1H, m, H-11a), 2.87 (1H, m, H-11b), 2.68 (3H, s, H-13), 2.35 (1H, m, H-7a), 2.23 (2H, m, H-10), 2.04 (1H, m, H-7b), 1.97~1.91 (2H, m, H-8); ^13^C NMR (150 MHz, CD_3_OD): *δ*_C_ 171.2 (C-5), 167.2 (C-2), 59.3 (C-6), 57.5 (C-3), 50.4 (C-11), 46.7 (C-9), 38.3 (C-13), 29.8 (C-7), 27.9 (C-10), 22.9 (C-8).

### 3.5. Anti-Parkinson’s Disease Assay

The anti-Parkinson’s disease assays were performed using a zebrafish (Danio rerio) model induced by the neurotoxin MPTP according to a previous method [18]. This model was selected based on its well-established relevance for PD research: zebrafish exhibit conserved dopaminergic neural circuits and neuro-chemical pathways with mammals, and MPTP specifically induces dopaminergic neuron degeneration accompanied by quantifiable motor deficits, thereby recapitulating core pathological features of PD. To ensure the validity of the model and the assay, both negative controls (blank and control groups) and a positive control (MPTP-induced model group) were included in the experimental design. The assay was performed according to a previous method. At a concentration of 50 μM, the anti-PD activities of four pairs of synthetic enantiomers **1**–**4** were evaluated along with MPTP.

Experimental Grouping: Take 1-day post-fertilization (1 dpf) zebrafish juveniles, remove their membranes and put them into a 6-well plate. Randomly place 30 fish into each well and set up 4 groups: zebrafish with no-treatment group (blank group, serving as a negative control), DMSO control group (control group, serving as a solvent negative control), MPTP-treated zebrafish (model group, serving as a positive control for PD induction) and zebrafish co-treated with MPTP and **1**–**4** group (treatment group).

MPTP and Samples Treatment: The control group was given 5 mL of 1 × 10^3^ fish culture water treatment. The model group was treated with 5 mL of 50 μM MPTP. The treatment group was treated with 5 mL of 50 μM plus 50 μM MPTP. Each group was processed continuously for 5 dpf.

Zebrafish Behavioral Test: The zebrafish larvae from each group were collected, cleaned and placed in 48-well plates (1 per well, with 1 mL of fish framing water) on the 5th day, and the testing time was 20 min. The locomotion of each larva was recorded using Zebrabox Revolution (ViewPoint, Lyon, France). Zebralab software (https://www.viewpoint.fr/product/zebrafish/visual-function/zebralab, accessed on 1 July 2025) was used to analyze the digital tracks, and the average speed was analyzed every 60 s.

## 4. Conclusions

In summary, four new MetO-containing diketopiperazines had been isolated from the marine sponge *Dysidea* sp., which were the first examples of diketopiperazines with stereochemically defined MetO from marine sponges. The structures with absolute configurations of these compounds were elucidated by spectroscopic analyses and total synthesis. In particular, SFC technology was used to separate the purchased synthetic precursors Fmoc-*L*- and Fmoc-*D*-MetO. Eventually, eight optically pure isomers were synthesized. Furthermore, compounds (+)-**1** and (−)-**1** exhibited potential anti-Parkinson’s disease activities in an in vivo zebrafish model. Future studies will focus on molecular docking to explore their interactions with potential targets such as MAO-B and adenosine A2A receptors. Our work could provide a reliable guidance for the diastereomer separation and structure determination of MetO-containing compounds as well as the study of anti-Parkinson’s drugs.

## Figures and Tables

**Table 1:** ^1^H and ^13^C NMR data of (+)-**1**, (+)-**2** and **3** (in CD_3_OD, *δ*, *J* in Hz).

No.	(+)-1		(+)-2		3	
	*δ*_H_	*δ*_C_	*δ*_H_	*δ*_C_	*δ*_H_	*δ*_C_
2	-	167.2	-	167.2	-	167.3
3	4.36 t (4.9)	55.2	4.39 t (5.3)	55.3	3.96 m	57.7
4	-	-	-	-	-	-
5	-	172.7	-	172.7	-	171.2
6	4.26 t (7.9)	60.4	4.30 t (7.9)	60.4	4.31 dd (9.9, 6.5)	59.4
7	a 2.31 m	29.2	a 2.35 m	29.3	a 2.35 m	29.9
	b 2.03 m		b 2.08 m		b 2.04 m	
8	2.01~1.98 m	23.5	2.04~2.02 m	23.5	1.94~1.92 m	23.0
9	3.57~3.48 m	46.4	3.61~3.53 m	46.5	a 3.63~3.58 m	46.8
					b 3.55~3.48 m	
10	2.30~2.26 m	23.9	2.34~2.29 m	23.8	2.24~2.20 m	28.0
11	3.00~2.26 m	50.0	a 3.09~3.02 m	49.8	a 2.99~2.93 m	50.6
			b 2.91 m		b 2.87 m	
13	2.66 s	38.2	2.71 s	37.9	2.67 s	38.2

## Data Availability

Data are contained within the article and Supplementary Materials.

## Supplementary Materials

The following supporting information can be downloaded at https://www.mdpi.com/article/10.3390/md23120477/s1. Figure S1: Chiral HPLC separation profile of (+)-3 and (−)-3; Figure S2: The UPC2 separation of Fmoc-*L*-MetO and Fmoc-*D*-MetO on Chiralpak IC-3 column; Figures S3–S6: X-ray crystallographic data of (*S*)-Fmoc-*D*-MetO, (*R*)-Fmoc-*D*-MetO, (+)-**1** and (−)-**1**; Figure S7–S80: The UV, IR, HR, ESI-MS and NMR spectra of all natural and synthetic compounds.
