# Supplementary material for: Four New Pairs of MetO-Containing Diketopiperazine Enantiomers: Isolation, Synthesis and Potential Anti-Parkinson’s Disease Activity
Source: Mar Drugs. 2025 Dec 13;23(12):477. doi: 10.3390/md23120477 (PMC12734934; doi:10.3390/md23120477)
Supplement: Supplementary file 1 [file marinedrugs-23-00477-s001.zip › marinedrugs-4002856-supplementary.pdf]

Supporting Information

Four new pairs of MetO-containing diketopiperazine enantiomers: isolation, synthesis and potential anti-Parkinson's Disease activity

Yu Lei[1,2,†], Zhenyu Yang[1,†], Daichun Li[1], Xiaojian Liao[1], Chamari Hettiarachchi[3,*], Bingxin Zhao[1,*], Shihai Xu[1,*]

[1] Department of Chemistry, College of Chemistry and Materials Science, Jinan University, Guangzhou, 510632, P. R. China

[2] Key Laboratory for Biobased Materials and Energy of Ministry of Education, College of Materials and Energy, South China Agricultural University, Guangzhou, 510642, P. R. China

[3] Molecular Biology & Biochemistry, Department of Chemistry, University of Colombo, Colombo 3, Sri Lanka

* Corresponding authors. Shi-Hai Xu, E-mail: txush@jnu.edu.cn; Bing-Xin Zhao, E-mail: zbx840622@163.com; Chamari Hettiarachchi, E-mail: chamarih@chem.cmb.ac.lk.

† These authors contributed equally to this work.

Table of Contents

1 Experimental section 1

1.1 (+)-**3** and (-)-**3** enantiomers separation 1

1.2 **Fmoc-*L*-** and **Fmoc-*D*-MetO** diastereomers separation 1

1.3 Physico-chemical constants of **Fmoc-*L*-** and **Fmoc-*D*-MetO** diastereomers 2

2 X-ray crystallographic data 4

2.1 X-ray crystallographic data for **(*S*)-Fmoc-*D*-MetO** 4

2.2 X-ray crystallographic data for **(*R*)-Fmoc-*D*-MetO** 4

2.3 X-ray crystallographic data for synthetic (+)-**1** 5

2.4 X-ray crystallographic data for synthetic (-)-**1** 6

3 UV, IR, HR-ESI-MS and NMR spectra 7

Fig. S7 UV spectrum of natural (+)-**1** 7

Fig. S8 IR spectrum of natural (+)-**1** 7

Fig. S9 HR-ESI-MS spectrum of natural (+)-**1** 8

Fig. S10 ^{1}H NMR spectrum (600 MHz, CD_3OD) of natural (+)-**1** 8

Fig. S11 ^{13}C NMR spectrum (150 MHz, CD_3OD) of natural (+)-**1** 9

Fig. S12 DEPT-135 spectrum of natural (+)-**1** 9

Fig. S13 ^{1}H-^{1}H COSY spectrum of natural (+)-**1** 10

Fig. S14 HSQC spectrum of natural (+)-**1** 10

Fig. S15 HMBC spectrum of natural (+)-**1** 11

Fig. S16 NOESY spectrum of natural (+)-**1** 11

Fig. S17 UV spectrum of natural (+)-**2** 12

Fig. S18 IR spectrum of natural (+)-**2** 12

Fig. S19 HR-ESI-MS spectrum of natural (+)-**2** 12

Fig. S20 ^{1}H NMR spectrum (600 MHz, CD_3OD) of natural (+)-**2** 13

Fig. S21 ^{13}C NMR spectrum (150 MHz, CD_3OD) of natural (+)-**2** 13

Fig. S22 DEPT-135 spectrum of natural (+)-**2** 14

Fig. S23 ^{1}H-^{1}H COSY spectrum of natural (+)-**2** 14

Fig. S24 HSQC spectrum of natural (+)-**2** 15
Fig. S25 HMBC spectrum of natural (+)-**2** 15
Fig. S26 NOESY spectrum of natural (+)-**2** 16
Fig. S27 UV spectrum of natural **3** 16
Fig. S28 IR spectrum of natural **3** 17
Fig. S29 HR-ESI-MS spectrum of natural **3** 17
Fig. S30 ^{1}H NMR spectrum (600 MHz, CD_3OD) of natural **3** 18
Fig. S31 ^{13}C NMR spectrum (150 MHz, CD_3OD) of natural **3** 18
Fig. S32 DEPT-135 spectrum of natural **3** 19
Fig. S33 ^{1}H-^{1}H COSY spectrum of natural **3** 19
Fig. S34 HSQC spectrum of natural **3** 20
Fig. S35 HMBC spectrum of natural **3** 20
Fig. S36 NOESY spectrum of natural **3** 21
Fig. S37 HR-ESI-MS spectrum of **(*S*)-Fmoc-*L*-MetO** 21
Fig. S38 ^{1}H NMR spectrum (600 MHz, DMSO-d_6) of **(*S*)-Fmoc-*L*-MetO** 22
Fig. S39 ^{13}C NMR spectrum (150 MHz, DMSO-d_6) of **(*S*)-Fmoc-*L*-MetO** 22
Fig. S40 HR-ESI-MS spectrum of **(*R*)-Fmoc-*L*-MetO** 23
Fig. S41 ^{1}H NMR spectrum (600 MHz, DMSO-d_6) of **(*R*)-Fmoc-*L*-MetO** 23
Fig. S42 ^{13}C NMR spectrum (150 MHz, DMSO-d_6) of **(*R*)-Fmoc-*L*-MetO** 24
Fig. S43 HR-ESI-MS spectrum of **(*S*)-Fmoc-*D*-MetO** 24
Fig. S44 ^{1}H NMR spectrum (600 MHz, DMSO-d_6) of **(*S*)-Fmoc-*D*-MetO** 25
Fig. S45 ^{13}C NMR spectrum (150 MHz, DMSO-d_6) of **(*S*)-Fmoc-*D*-MetO** 25
Fig. S46 HR-ESI-MS spectrum of **(*R*)-Fmoc-*D*-MetO** 26
Fig. S47 ^{1}H NMR spectrum (600 MHz, DMSO-d_6) of **(*R*)-Fmoc-*D*-MetO** 26
Fig. S48 ^{13}C NMR spectrum (150 MHz, DMSO-d_6) of **(*R*)-Fmoc-*D*-MetO** 27
Fig. S49 HR-ESI-MS spectrum of **A1** 27
Fig. S50 HR-ESI-MS spectrum of **B1** 28
Fig. S51 HR-ESI-MS spectrum of **C1** 28
Fig. S52 HR-ESI-MS spectrum of **D1** 29
Fig. S53 HR-ESI-MS spectrum of **E1** 29

Fig. S54 HR-ESI-MS spectrum of **F1** 30

Fig. S55 HR-ESI-MS spectrum of **G1** 30

Fig. S56 HR-ESI-MS spectrum of **H1** 31

Fig. S57 HR-ESI-MS spectrum of synthetic (+)-**1** 31

Fig. S58 ^{1}H NMR spectrum (600 MHz, CD_3OD) of synthetic (+)-**1** 32

Fig. S59 ^{13}C NMR spectrum (150 MHz, CD_3OD) of synthetic (+)-**1** 32

Fig. S60 HR-ESI-MS spectrum of synthetic (-)-**1** 33

Fig. S61 ^{1}H NMR spectrum (600 MHz, CD_3OD) of synthetic (-)-**1** 33

Fig. S62 ^{13}C NMR spectrum (150 MHz, CD_3OD) of synthetic (-)-**1** 34

Fig. S63 HR-ESI-MS spectrum of synthetic (+)-**2** 34

Fig. S64 ^{1}H NMR spectrum (600 MHz, CD_3OD) of synthetic (+)-**2** 35

Fig. S65 ^{13}C NMR spectrum (150 MHz, CD_3OD) of synthetic (+)-**2** 35

Fig. S66 HR-ESI-MS spectrum of synthetic (-)-**2** 36

Fig. S67 ^{1}H NMR spectrum (600 MHz, CD_3OD) of synthetic (-)-**2** 36

Fig. S68 ^{13}C NMR spectrum (150 MHz, CD_3OD) of synthetic (-)-**2** 37

Fig. S69 HR-ESI-MS spectrum of synthetic (+)-**3** 37

Fig. S70 ^{1}H NMR spectrum (600 MHz, CD_3OD) of synthetic (+)-**3** 38

Fig. S71 ^{13}C NMR spectrum (150 MHz, CD_3OD) of synthetic (+)-**3** 38

Fig. S72 HR-ESI-MS spectrum of synthetic (-)-**3** 39

Fig. S73 ^{1}H NMR spectrum (600 MHz, CD_3OD) of synthetic (-)-**3** 39

Fig. S74 ^{13}C NMR spectrum (150 MHz, CD_3OD) of synthetic (-)-**3** 40

Fig. S75 HR-ESI-MS spectrum of synthetic (+)-**4** 40

Fig. S76 ^{1}H NMR spectrum (600 MHz, CD_3OD) of synthetic (+)-**4** 41

Fig. S77 ^{13}C NMR spectrum (150 MHz, CD_3OD) of synthetic (+)-**4** 41

Fig. S78 HR-ESI-MS spectrum of synthetic (-)-**4** 42

Fig. S79 ^{1}H NMR spectrum (600 MHz, CD_3OD) of synthetic (-)-**4** 42

Fig. S80 ^{13}C NMR spectrum (150 MHz, CD_3OD) of synthetic (-)-**4** 43

1 Experimental section

1.1 (+)-**3** and (-)-**3** enantiomers separation

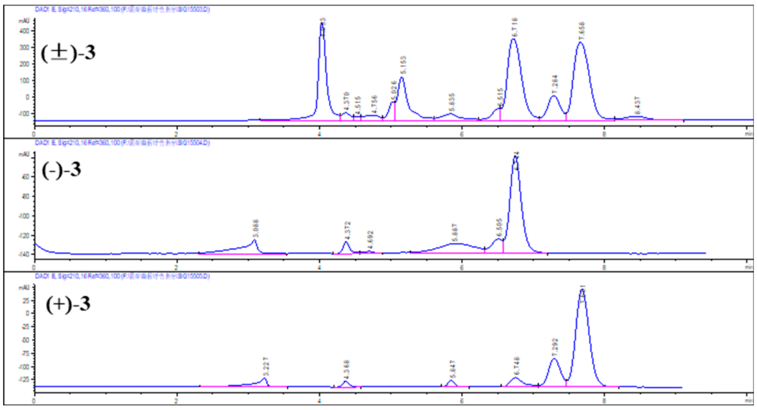

Fig. S1 Chiral HPLC separation profile of (+)-**3** and (-)-**3.**

1.2 **Fmoc-*L*-** and **Fmoc-*D*-MetO** diastereomers separation

The Fmoc-*L*-MetO diastereomers were purified by Waters SFC Prep 15 system using semi-preparative Chiralpak IC-3 column (5 μm, 10 × 250 mm, CH_3OH/CO_2/0.1 % TFA = 30/70, flow rate = 5.0 mL/min) to yield (*S*)-Fmoc-*L*-MetO (2.3 g, t_R = 7.8 min), (*R*)-Fmoc-*L*-MetO (2.5 g, t_R = 9.0 min). The Fmoc-*D*-MetO diastereomers were also separated using the same method to obtain (*S*)-Fmoc-*D*-MetO (2.5 g, t_R = 10.8 min) and (*R*)-Fmoc-*D*-MetO (2.0 g, t_R = 12.2 min).

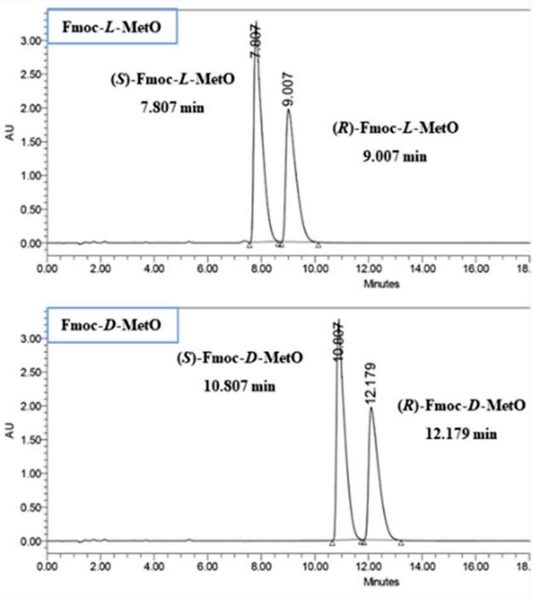

Fig. S2 The UPC^2 separation of Fmoc-*L*-MetO and Fmoc-*D*-MetO on Chiralpak IC-3 column (4.6 × 150 mm, 3 μm; 2.5 mL/min, 10 % MeOH/CO_2/0.1 % TFA).

1.3 Physico-chemical constants of **Fmoc-*L*-** and **Fmoc-*D*-MetO** diastereomers

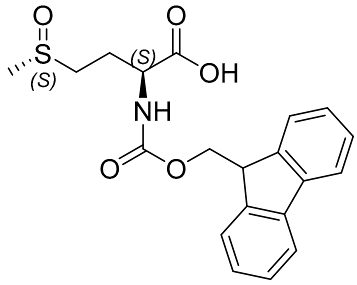

(*S*)-Fmoc-*L*-MetO: white amorphous solid; mp. 180-181 °C; HR-ESI-MS *m/z*: 388.1225 [M + H]$^+$ (calcd for $C_{20}H_{22}NO_5S$: 388.1223); ^{1}H NMR (600 MHz, DMSO-d_6): δ_H 7.84 (2H, d, J = 7.6 Hz), 7.74~7.64 (2H, m), 7.37 (2H, t, J = 7.4 Hz), 7.33 (2H, td, J = 7.5, 2.0 Hz), 4.32-4.24 (2H, m), 4.18 (1H, t, J = 7.1 Hz), 4.03 (1H, td, J = 8.7, 4.8 Hz), 2.77~2.63 (2H, m), 2.50 (3H, s), 2.06 (1H, m), 1.91 (1H, m); ^{13}C NMR (150 MHz, DMSO-d_6): δ_C 173.1, 156.2, 143.8, 143.8, 140.8, 127.7, 127.1, 125.3, 120.2, 65.7, 53.1, 49.8, 46.7, 38.1, 24.3.

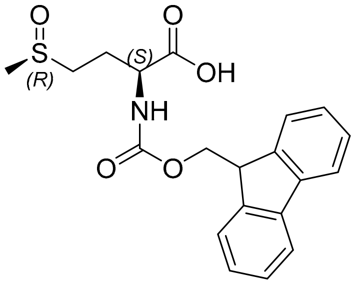

(*R*)-Fmoc-*L*-MetO: white amorphous solid; mp. 179-180 °C; HR-ESI-MS *m/z*: 388.1219 [M + H]$^+$ (calcd for $C_{20}H_{22}NO_5S$ 388.1223); ^{1}H NMR (600 MHz, DMSO-d_6): δ_H 7.87 (2H, d, J = 7.5 Hz), 7.76~7.66 (2H, m), 7.40 (2H, t, J = 7.5 Hz), 7.31 (2H, td, J = 7.4, 1.4 Hz), 4.29 (2H, d, J = 7.0 Hz), 4.21 (1H, t, J = 7.1 Hz), 4.07 (1H, m), 2.82 (1H, m), 2.63 (1H, m), 2.50 (3H, s), 2.10 (1H, m), 1.95 (1H, m); ^{3}C NMR (150 MHz, DMSO-d_6): δ_C 173.1, 156.2, 143.8, 143.8, 140.7, 127.7, 127.1, 125.3, 120.2, 120.1, 65.7, 52.8, 49.5, 46.7, 37.8, 23.7.

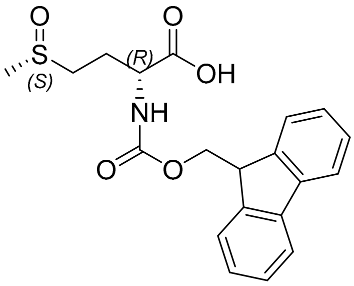

(*S*)-Fmoc-*D*-MetO: white needle-like crystals (CH_2Cl_2: MeOH=1:1); mp. 179-180 °C; HR-ESI-MS *m/z*: 388.1225 $[M + H]^+$ (calcd for $C_{20}H_{22}NO_5S$: 388.1223); ^1H NMR (600 MHz, DMSO-d_6): δ_H 7.88 (2H, d, *J* = 7.5 Hz), 7.77~7.68 (2H, m), 7.41 (2H, t, *J* = 7.5 Hz), 7.32 (2H, td, *J* = 7.4, 1.4 Hz), 4.30 (2H, d, *J* = 7.0 Hz), 4.22 (1H, t, *J* = 7.1 Hz), 4.08 (1H, td, *J* = 9.0, 4.4 Hz), 2.83 (1H, m), 2.64 (1H, m), 2.52 (3H, s), 2.11 (1H, m), 1.96 (1H, m); ^{13}C NMR (150 MHz, DMSO-d_6): δ_C 173.1, 156.1, 143.8, 143.8, 140.7, 127.7, 127.1, 125.3, 120.1, 120.1, 65.6, 52.8, 49.5, 46.7, 37.8, 23.7.

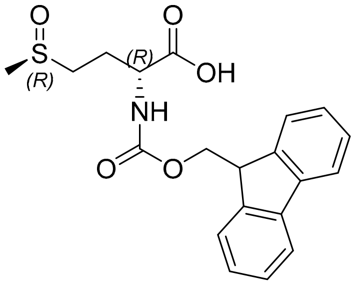

(*R*)-Fmoc-*D*-MetO: white needle-like crystals (CH_2Cl_2: MeOH=1:1); mp. 180-181 °C; HR-ESI-MS *m/z*: 388.1225 $[M + H]^+$ (calcd for $C_{20}H_{22}NO_5S$: 388.1223); ^1H NMR (600 MHz, DMSO-d_6): δ_H 7.89 (2H, d, *J* = 7.5 Hz), 7.79~7.70 (2H, m), 7.42 (2H, td, *J* = 7.4, 1.2 Hz), 7.33 (2H, td, *J* = 7.4, 1.3 Hz), 4.31 (2H, d, *J* = 7.8 Hz), 4.23 (1H, t, *J* = 7.0 Hz), 4.09 (1H, td, *J* = 8.7, 4.8 Hz), 2.75 (2H, m), 2.55 (3H, s), 2.12 (1H, m), 1.95 (1H, m); ^{13}C NMR (150 MHz, DMSO-d_6): δ_C 173.1, 156.1, 143.8, 143.8, 140.7, 127.7, 127.1, 125.3, 120.1, 65.7, 53.1, 49.8, 46.7, 38.1, 24.2.

2 X-ray crystallographic data

2.1 X-ray crystallographic data for **(*S*)-Fmoc-*D*-MetO**

Crystal Data: Upon crystallization from CH_3OH using the vapor diffusion method, needles of **(*S*)-Fmoc-*D*-MetO** were obtained. $C_{20}H_{23}NO_6S$ (M = 406.48 g/mol); orthorhombic, space group $P2_12_12_1$; a = 5.58061 (16) Å, b = 14.7294 (5) Å, c = 23.8516 (9) Å, V = 1960.58 (12) Å^3, Z = 4, T = 120.00 K, μ (Cu Kα) = 1.792 mm^{-1}, D_{cacl} = 1.3770 g/cm^3, 6306 reflections measured (7.06° ≤ 2θ ≤ 147. 72°), 3452 unique (R_{int} = 0.0463), which were used in all calculations. The final R_1 value was 0.0606 ($I > 2\sigma(I)$). The final R_1 value was 0.0664 (all data). The final wR_2 value was 0.1688 (all data). The goodness of fit for F^2 was 1.049. Flack parameter = 0.02 (3). Crystallographic data for **(*S*)-Fmoc-*D*-MetO** have been deposited in the Cambridge Crystallographic Data Center as a supplementary publication. Copies of the data can be obtained, free of charge, on application to the Director, CCDC 2491344, 12 Union Road, Cambridge CB2 1EZ, UK (fax: +44-(0)1223−336033, or email: deposit@ccdc.cam.ac.uk).

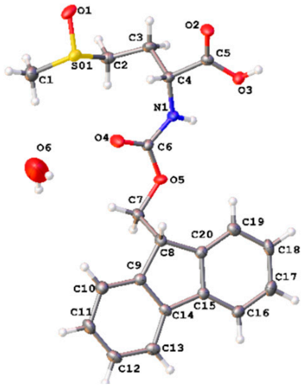

Fig. S3 X-ray (Cu Kα) ORTEP drawings of **(*S*)-Fmoc-*D*-MetO.**

2.2 X-ray crystallographic data for **(*R*)-Fmoc-*D*-MetO**

Crystal Data: Upon crystallization from CH_3OH using the vapor diffusion method, needles of **(*R*)-Fmoc-*D*-MetO** were obtained. $C_{20}H_{21}NO_5S$ (M = 387.45 g/mol); monoclinic, space group $P2_1$; a = 5.4401 (1) Å, b = 15.6876 (2) Å, c = 11.0463 (2) Å, V = 941.26 (3) Å^3, Z = 2, T = 120.00 (10) K, μ (Cu Kα) = 1.846 mm^{-1}, D_{cacl} = 1.3810 g/cm^3, 7033 reflections measured (8.02° ≤ 2θ ≤ 147. 26°), 3691 unique (R_{int} = 0.0362), which were used in all calculations. The final R_1 value was 0.0452 ($I > 2\sigma(I)$). The final

R_1 value was 0.0469 (all data). The final wR_2 value was 0.1223 (all data). The goodness of fit for F^2 was 1.056. Flack parameter = -0.00 (2). Crystallographic data for **(*S*)-Fmoc-*D*-MetO** have been deposited in the Cambridge Crystallographic Data Center as a supplementary publication. Copies of the data can be obtained, free of charge, on application to the Director, CCDC 2491347, 12 Union Road, Cambridge CB2 1EZ, UK (fax: +44-(0)1223−336033, or email: deposit@ccdc.cam.ac.uk).

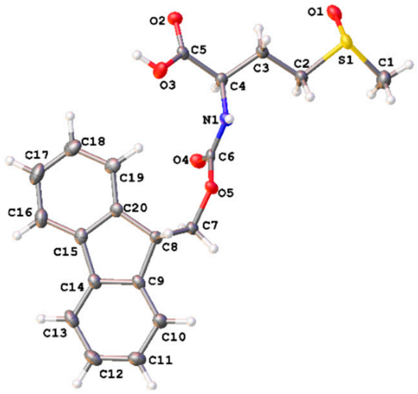

Fig. S4 X-ray (Cu Kα) ORTEP drawings of **(*R*)-Fmoc-*D*-MetO.**

2.3 X-ray crystallographic data for synthetic (+)-**1**

Crystal Data: Upon crystallization from CH_3OH using the vapor diffusion method, needles of (+)-**1** were obtained. $C_{10}H_6N_2O_3S$ (M = 257.22 g/mol); rthorhombic, space group $P2_12_12_1$; α = 8.88080 (10) Å, b = 9.14320 (10) Å, c = 14.2998 (2) Å, V = 1161.13 (2) Å^3, Z = 5, T = 169.99 (10) K, μ (Cu Kα) = 3.562 mm^{-1}, D_{cacl} = 1.839 g/cm^3, 7070 reflections measured (8.02° ≤ 2θ ≤ 147. 26°), 2295 unique (R_{int} = 0.0323), which were used in all calculations. The final R_1 value was 0.0380 ($I > 2\sigma(I)$). The final wR_2 value was 0.0981 ($I > 2\sigma(I)$). The final R_1 value was 0.0383 (all data). The final wR_2 value was 0.0983 (all data). The goodness of fit for F^2 was 1.066. Flack parameter = 0.013 (9). Crystallographic data for (+)-**1** have been deposited in the Cambridge Crystallographic Data Center as a supplementary publication. Copies of the data can be obtained, free of charge, on application to the Director, CCDC 2491346, 12 Union Road, Cambridge CB2 1EZ, UK (fax: +44-(0)1223−336033, or email: deposit@ccdc.cam.ac.uk).

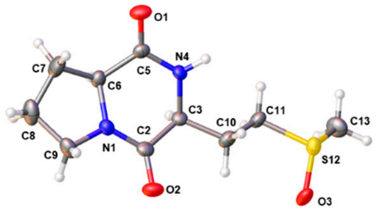

Fig. S5 X-ray (Cu Kα) ORTEP drawings of synthetic (+)-**1.**

2.4 X-ray crystallographic data for synthetic (-)-1

Crystal Data: Upon crystallization from CH_3OH using the vapor diffusion method, needles of (-)-**1** were obtained. $C_{10}H_{16}N_2O_3S$ (M = 244.32 g/mol); orthorhombic, space group $P2_12_12_1$; α = 8.8778 (2) Å, b = 9.1026 (2) Å, c = 14.2677 (2) Å, V = 1152.99 (4) Å^3, Z = 4, T = 119.99 (10) K, μ (Cu Kα) = 2.477 mm^{-1}, D_{cacl} = 1.4074 g/cm^3, 6311 reflections measured (11.54° ≤ 2θ ≤ 147. 94°), 2269 unique (R_{int} =0.0274), which were used in all calculations. The final R_1 value was 0.0329 ($I > 2\sigma(I)$). The final R_1 value was 0.0332 (all data). The final wR_2 value was 0.0846 (all data). The goodness of fit for F^2 was 1.056. Flack parameter = 0.002 (14). Crystallographic data for (-)-**1** have been deposited in the Cambridge Crystallographic Data Center as a supplementary publication. Copies of the data can be obtained, free of charge, on application to the Director, CCDC 2491345, 12 Union Road, Cambridge CB2 1EZ, UK (fax: +44-(0)1223−336033, or email: deposit@ccdc.cam.ac.uk).

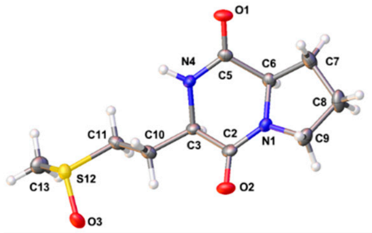

Fig. S6 X-ray (Cu Kα) ORTEP drawings of synthetic (-)-**1.**

3 UV, IR, HR-ESI-MS and NMR spectra

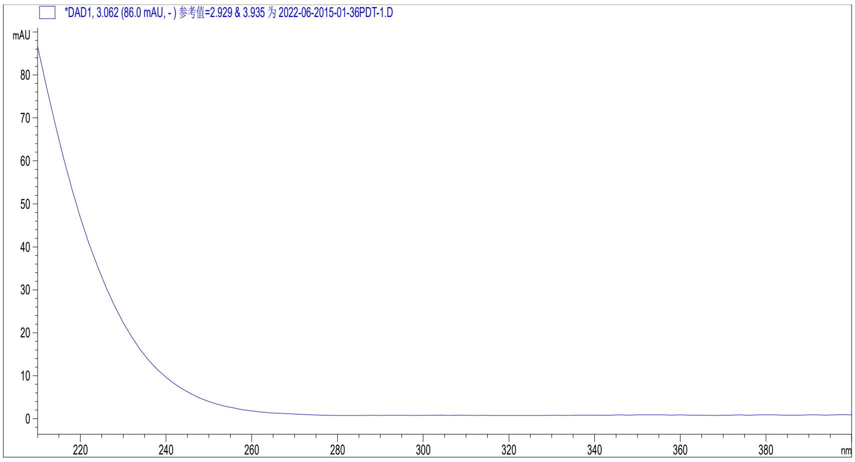

Fig. S7 UV spectrum of natural (+)-**1.**

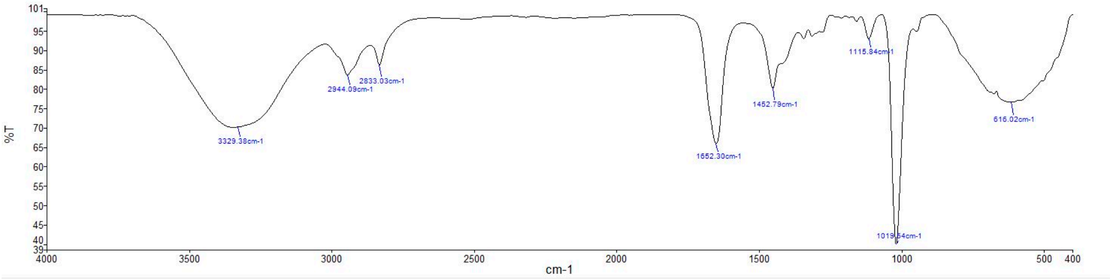

Fig. S8 IR spectrum of natural (+)-**1.**

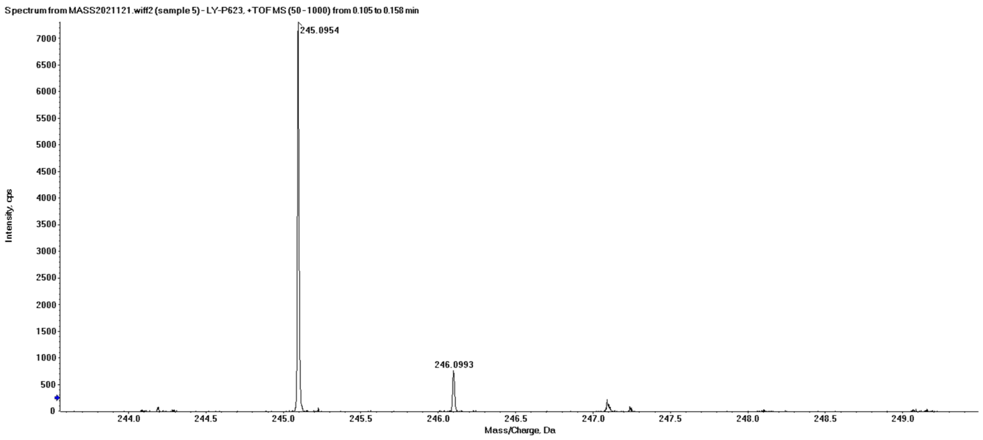

Hit	Formula	m/z	RDB	ppm	MS Rank	MSMS ppm	MSMS Rank	Found
1	$C_{10}H_{16}N_2O_3S$	245.0954	4.0	-0.2	1			NA/NA

Fig. S9 HR-ESI-MS spectrum of natural (+)-**1.**

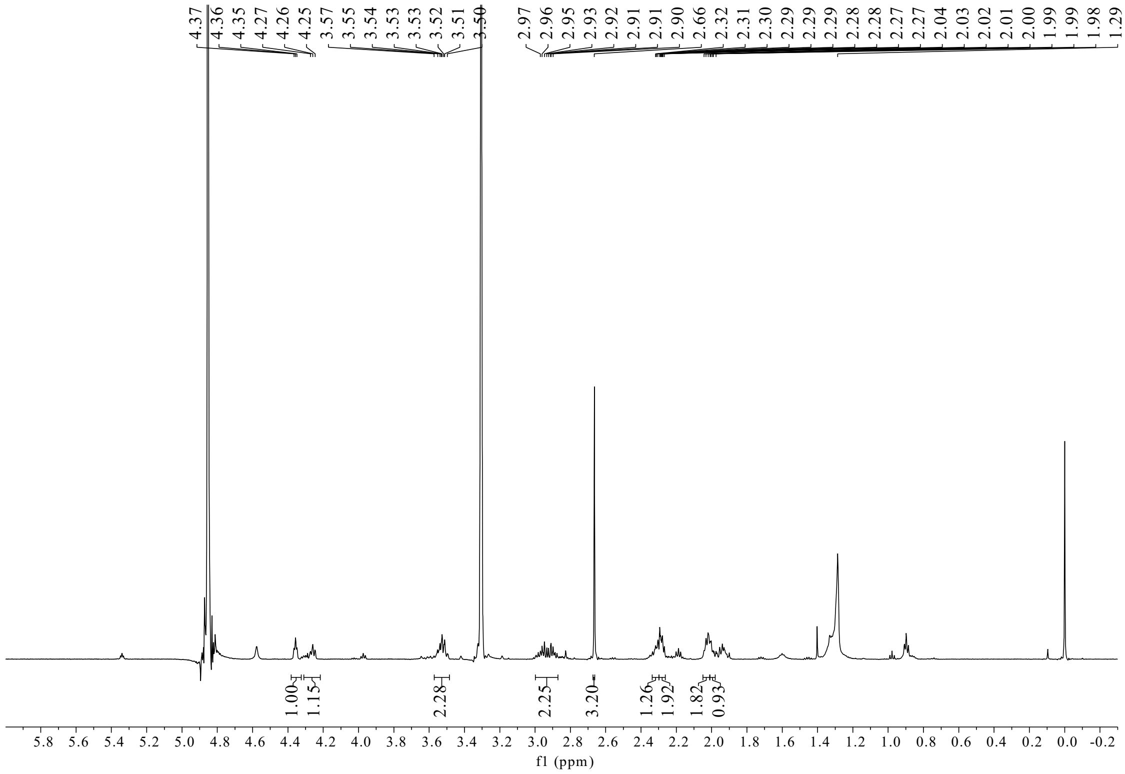

Fig. S10 ^{1}H NMR spectrum (600 MHz, CD_3OD) of natural (+)-**1.**

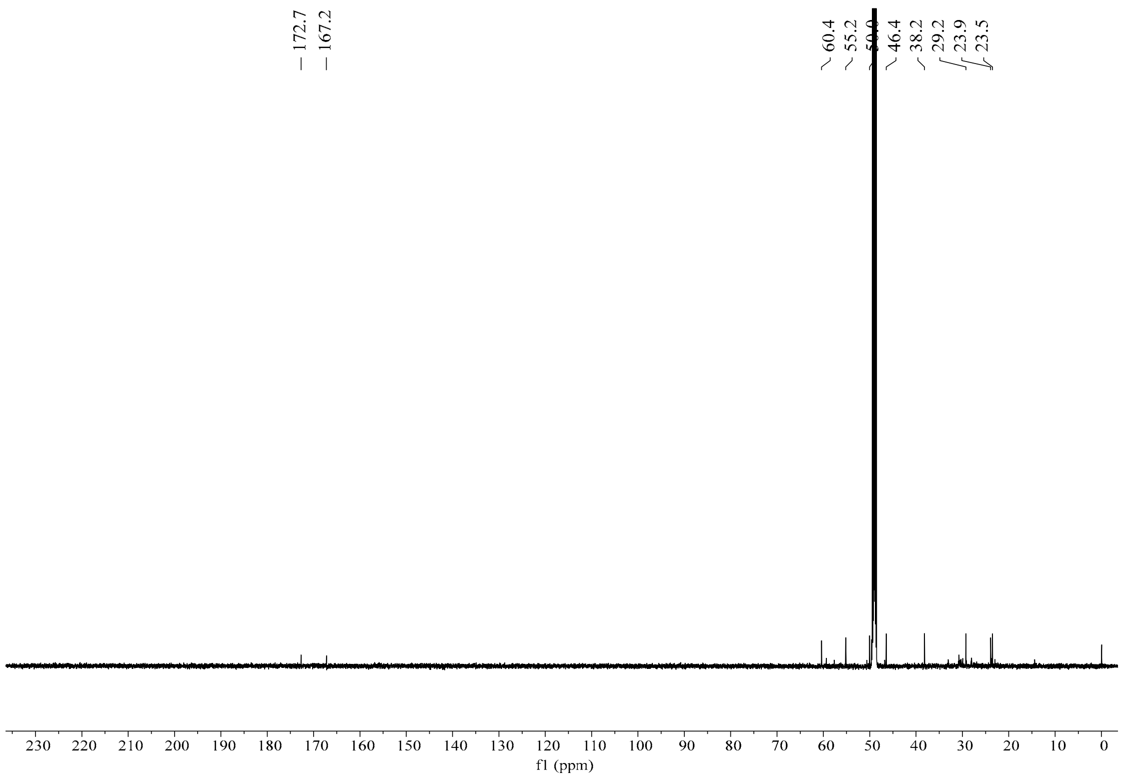

Fig. S11 ^{13}C NMR spectrum (150 MHz, CD_3OD) of natural (+)-**1.**

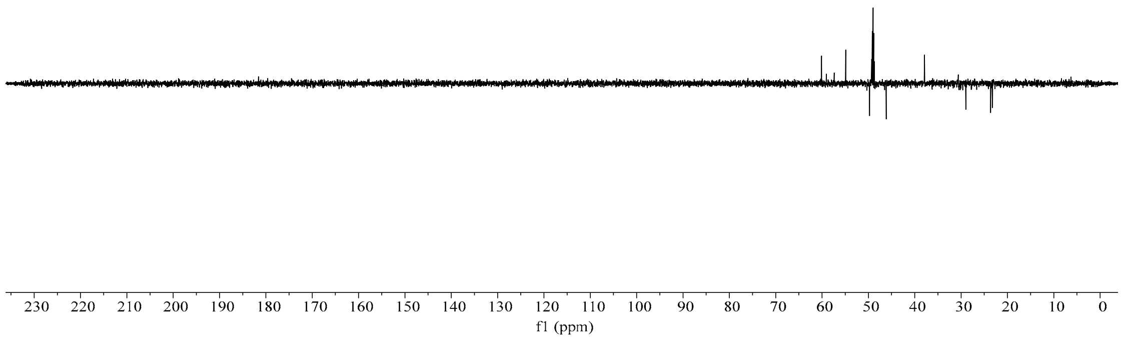

Fig. S12 DEPT-135 spectrum of natural (+)-**1.**

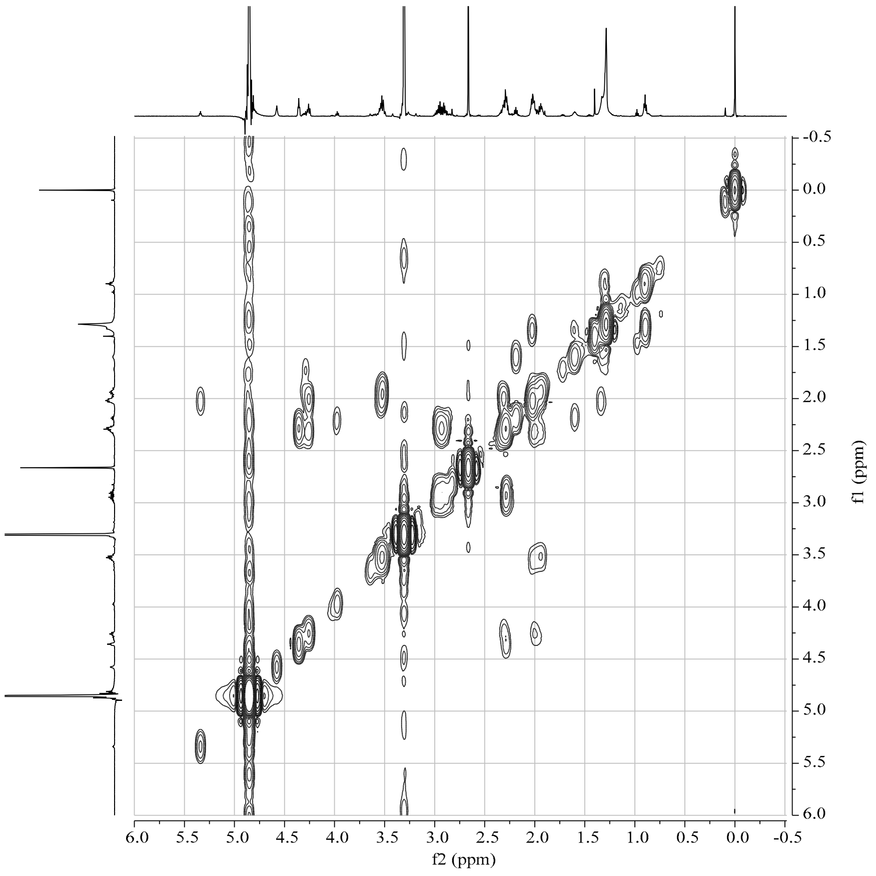

Fig. S13 ^{1}H-^{1}H COSY spectrum of natural (+)-**1.**

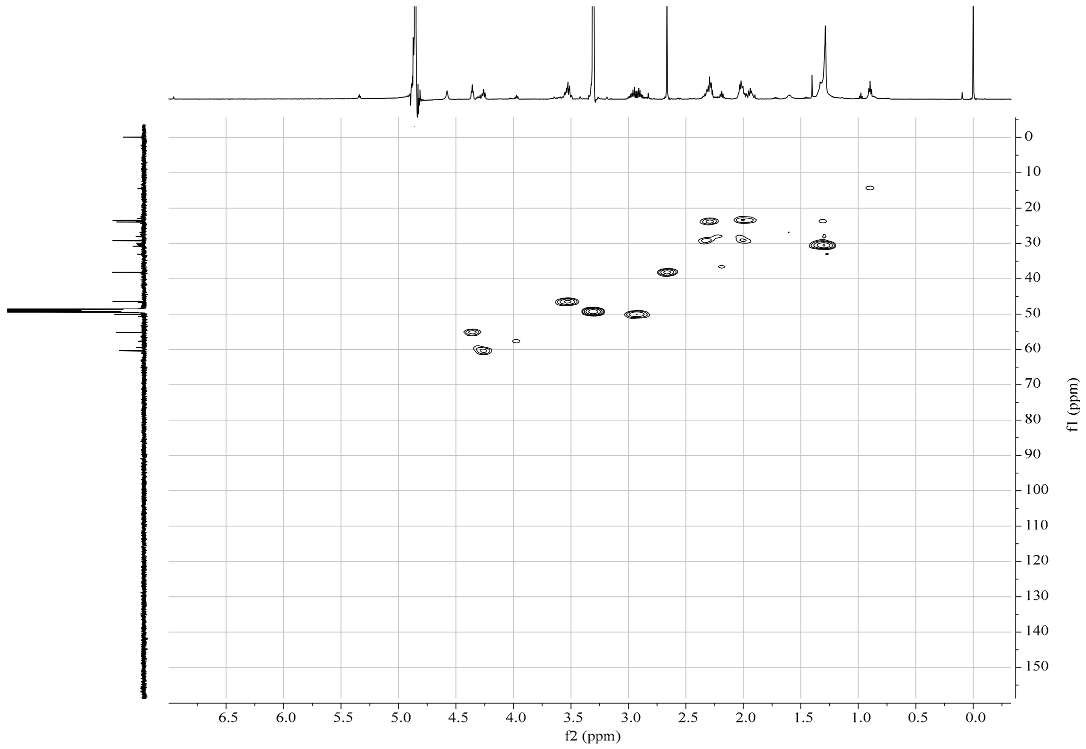

Fig. S14 HSQC spectrum of natural (+)-**1.**

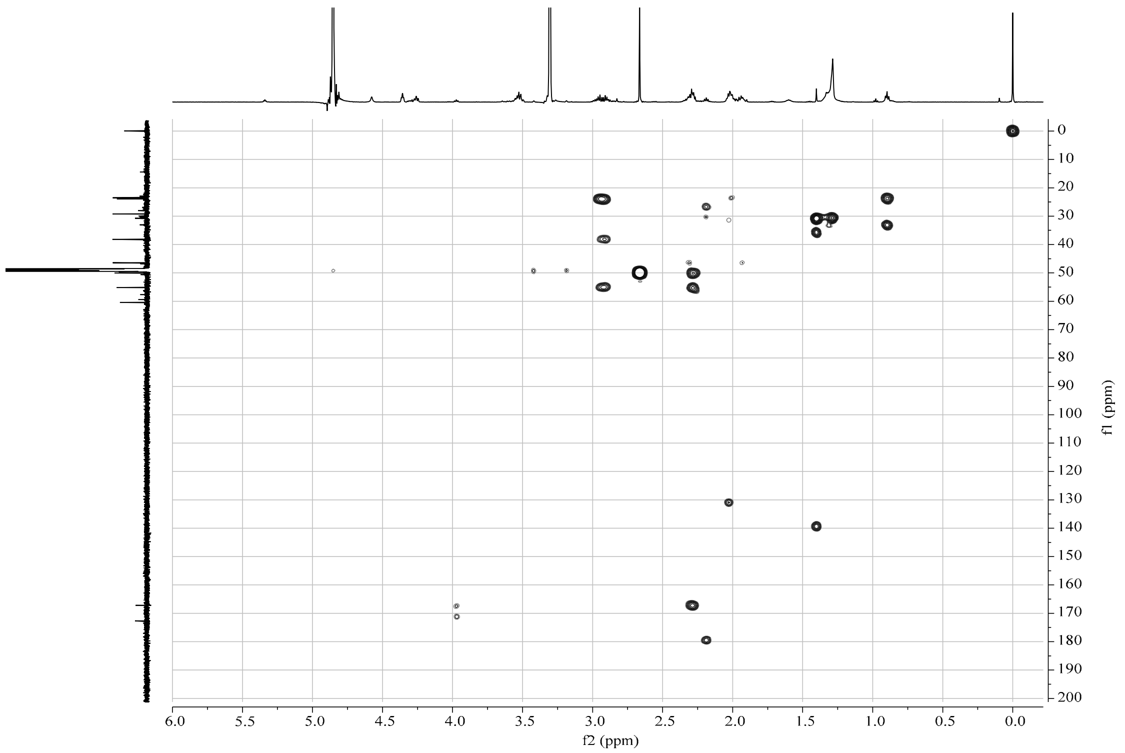

Fig. S15 HMBC spectrum of natural (+)-**1.**

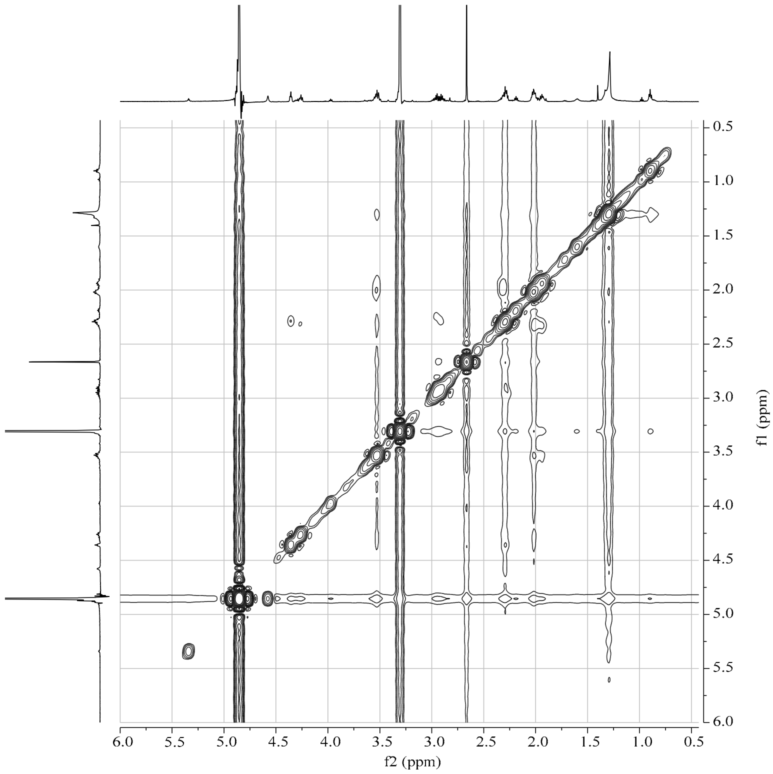

Fig. S16 NOESY spectrum of natural (+)-**1.**

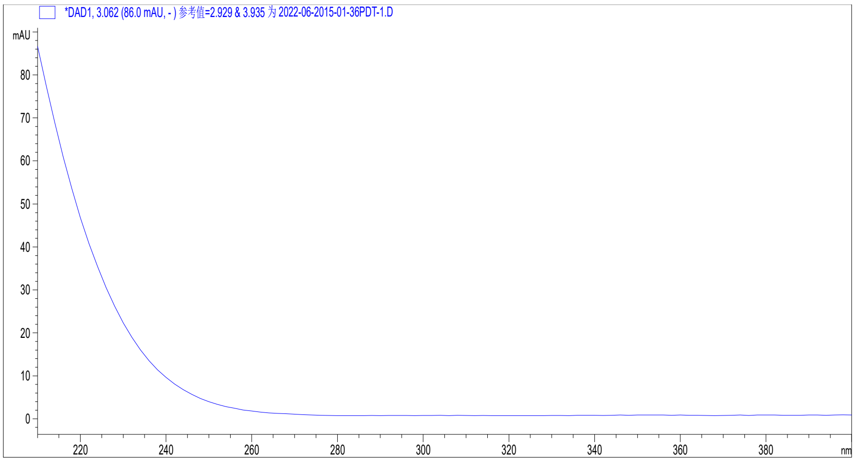

Fig. S17 UV spectrum of natural (+)-**2.**

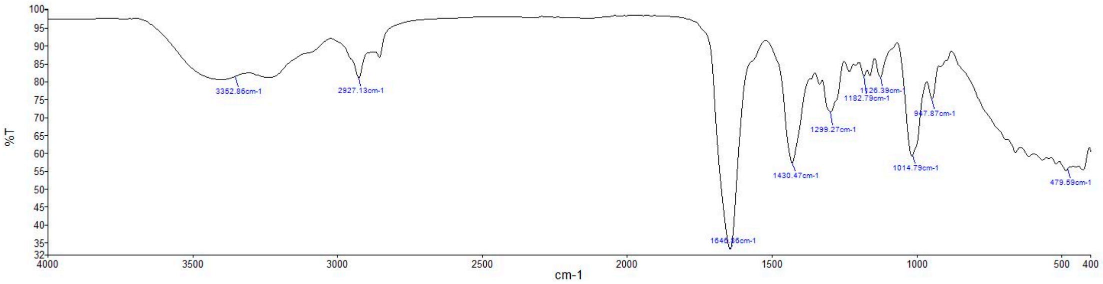

Fig. S18 IR spectrum of natural (+)-**2.**

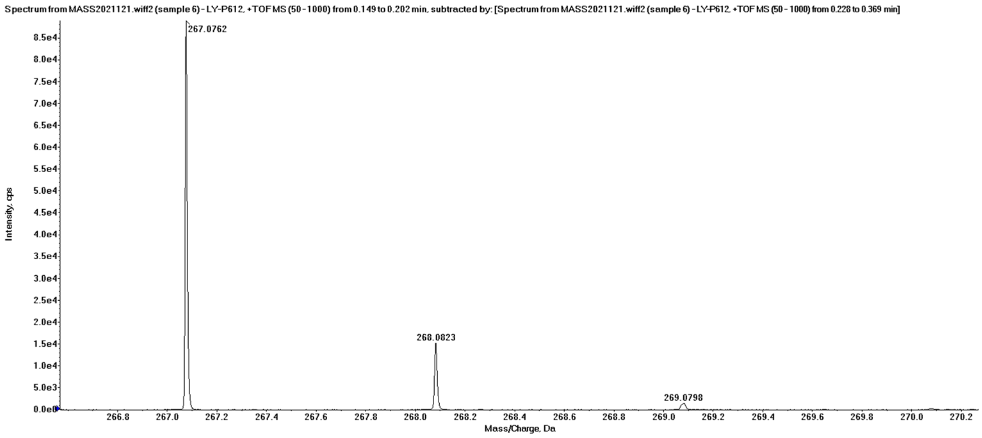

Hit	Formula	m/z	RDB	ppm	MS Rank	MSMS ppm	MSMS Rank	Found
1	$C_{10}H_{16}N_2O_3S$	267.0774	4.0	-4.4	1			NA/NA

Fig. S19 HR-ESI-MS spectrum of natural (+)-**2.**

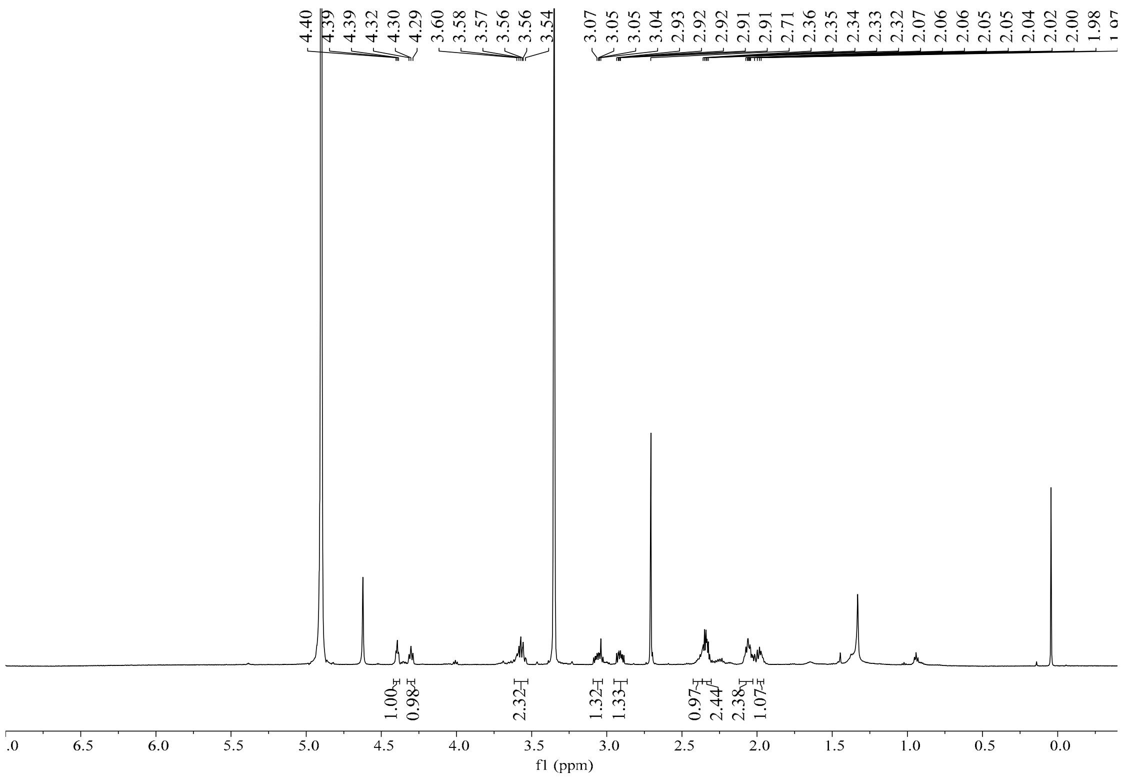

Fig. S20 ^{1}H NMR spectrum (600 MHz, CD3OD) of natural (+)-**2.**

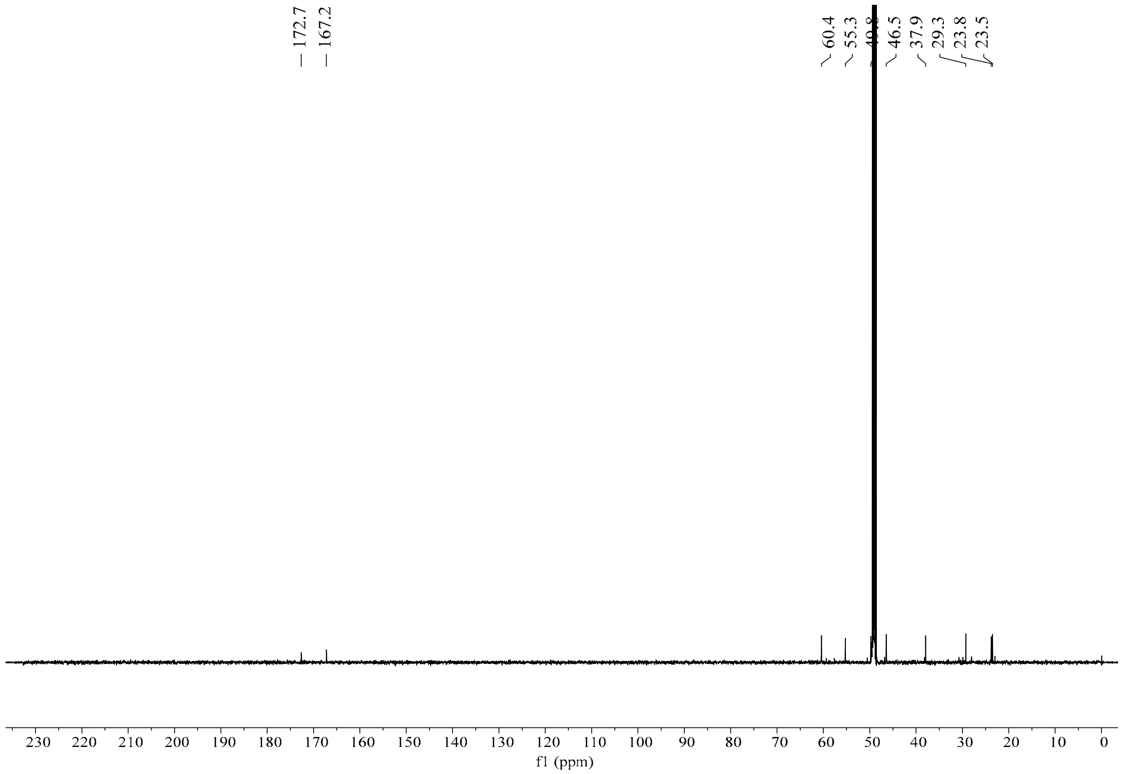

Fig. S21 ^{13}C NMR spectrum (150 MHz, CD3OD) of natural (+)-**2.**

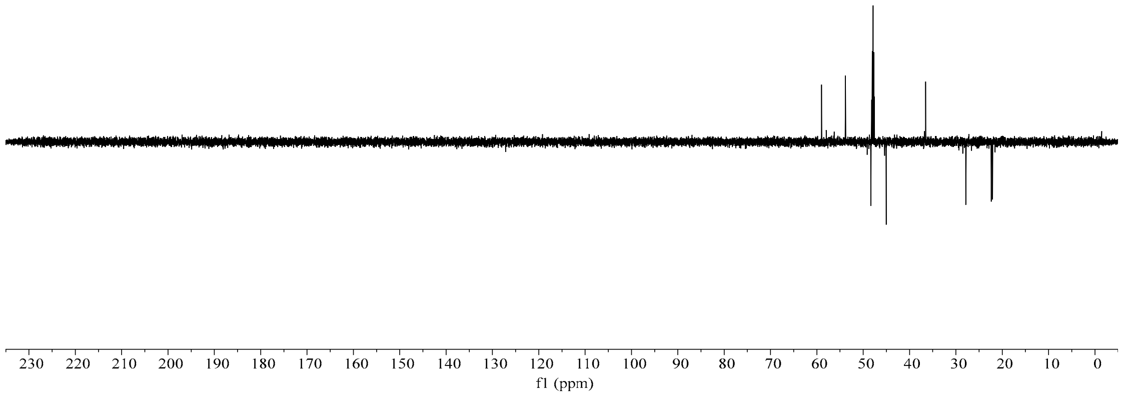

Fig. S22 DEPT-135 spectrum of natural (+)-**2.**

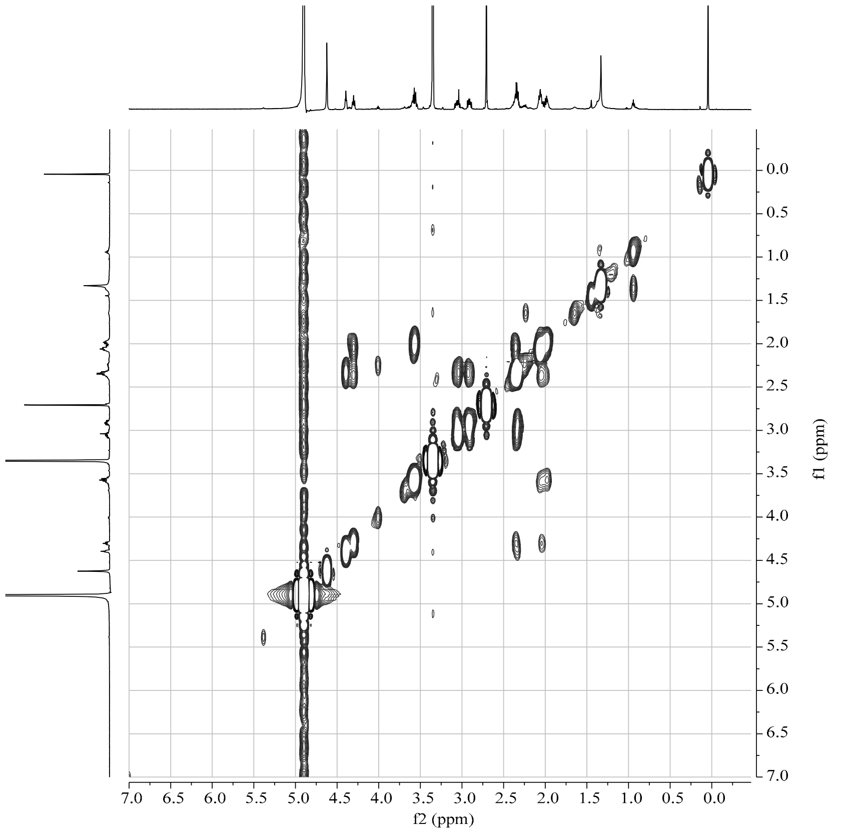

Fig. S23 ¹H-¹H COSY spectrum of natural (+)-**2.**

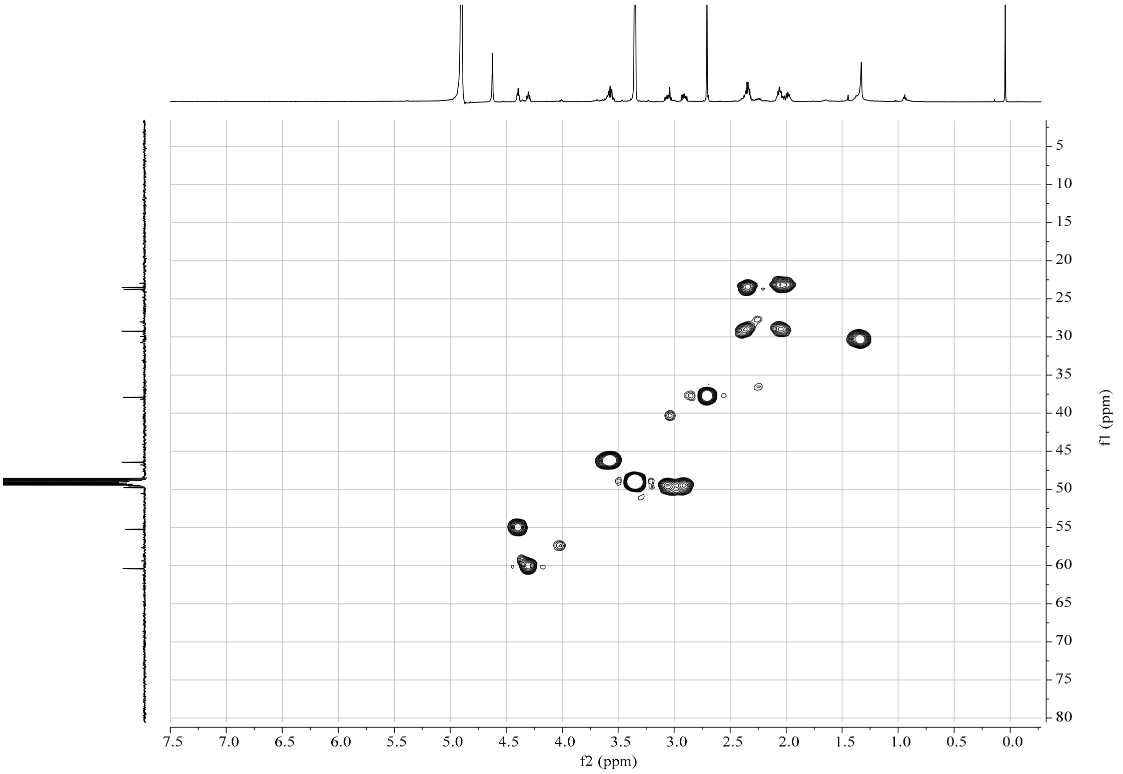

Fig. S24 HSQC spectrum of natural (+)-**2.**

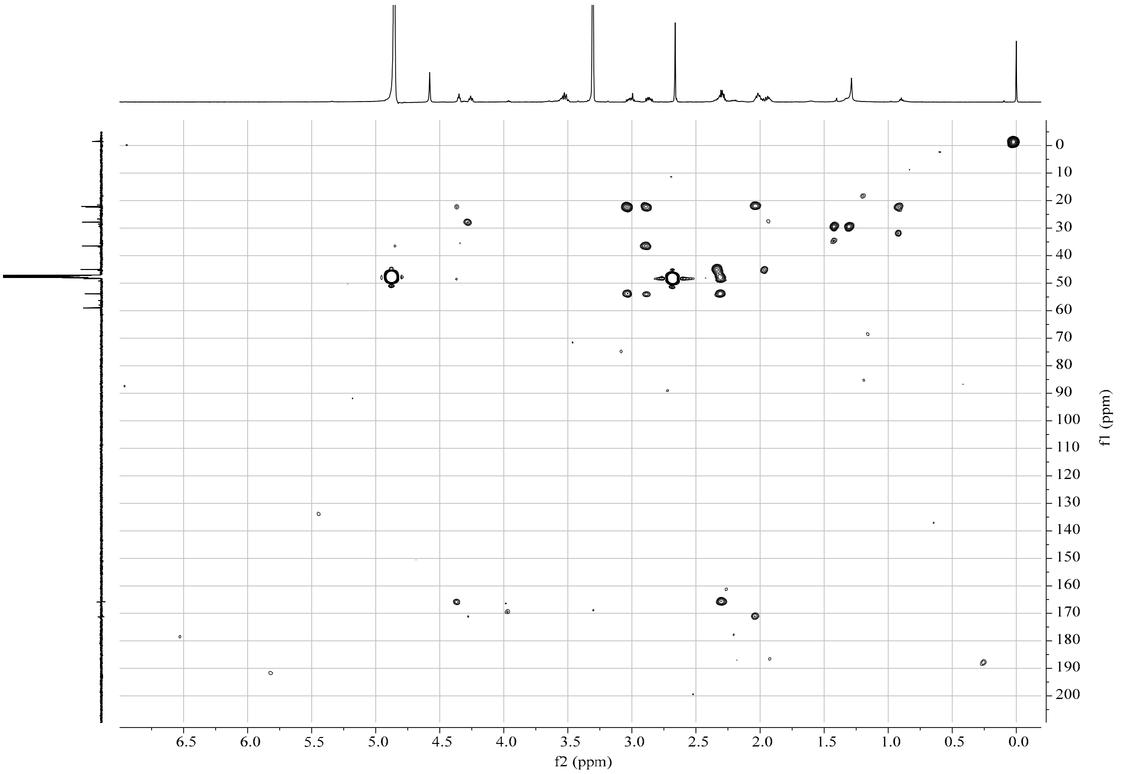

Fig. S25 HMBC spectrum of natural (+)-**2.**

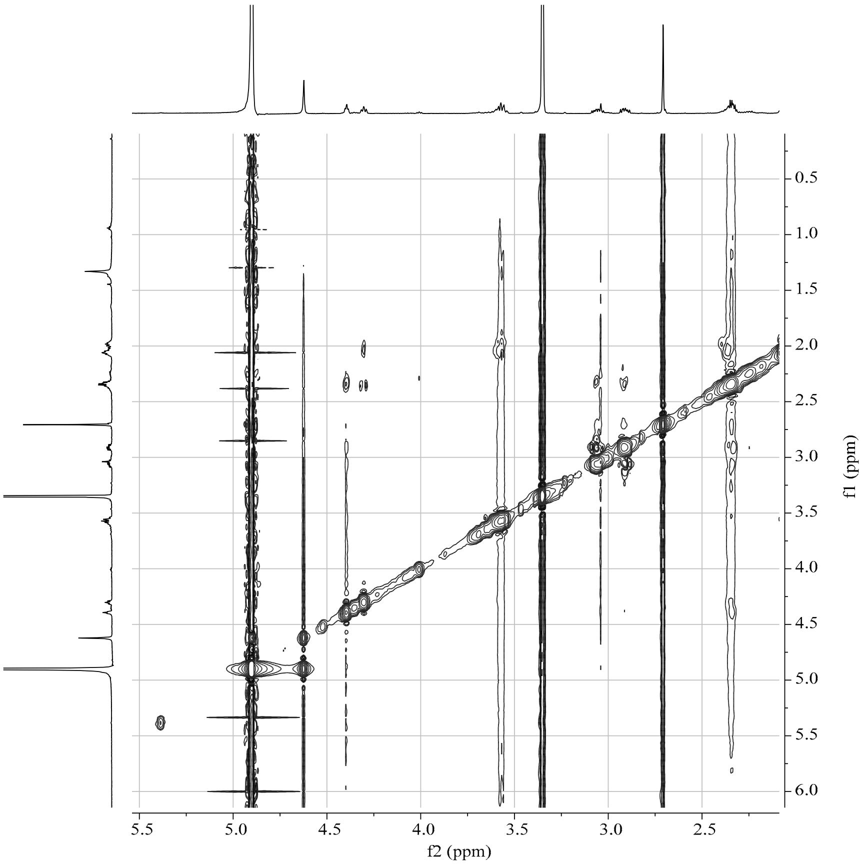

Fig. S26 NOESY spectrum of natural (+)-**2.**

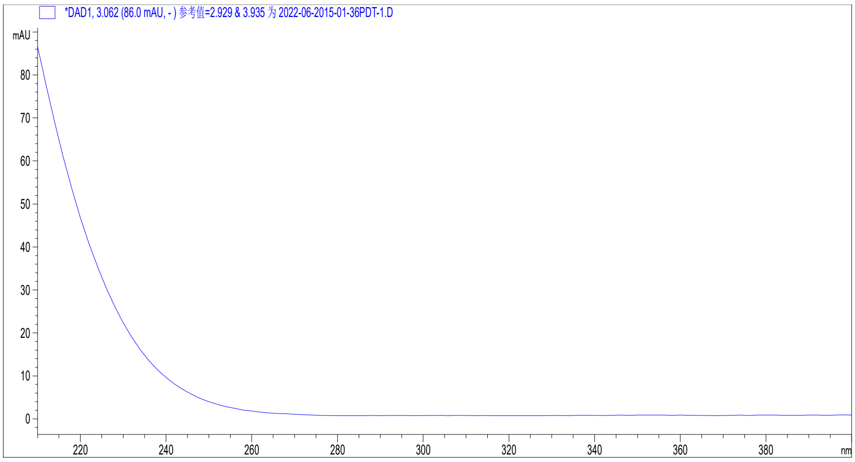

Fig. S27 UV spectrum of natural **3.**

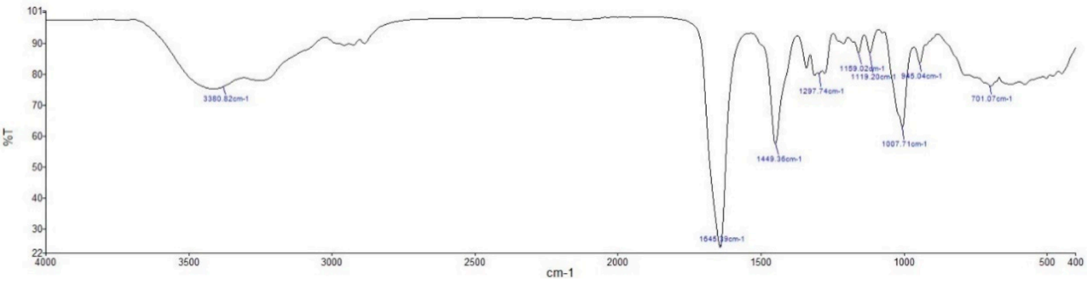

Fig. S28 IR spectrum of natural **3.**

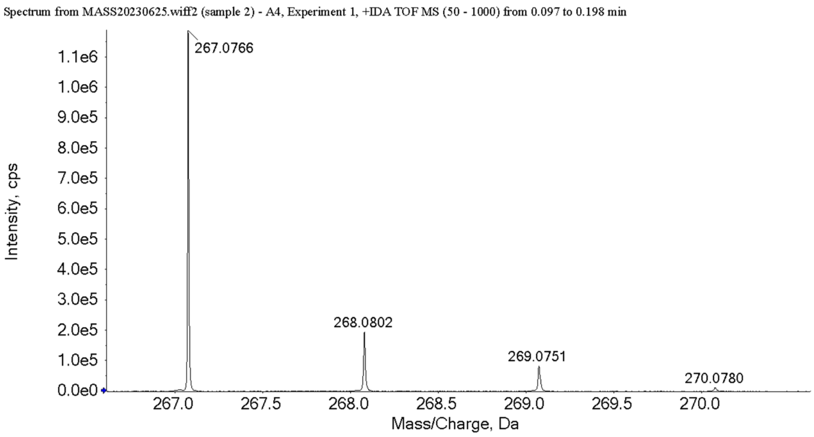

Hit	Formula	m/z	RDB	ppm	MS Rank	MSMS ppm	MSMS Rank	Found
1	$C_{10}H_{16}N_2O_3S$	267.0774	4.0	-2.9	1			NA/NA

Fig. S29 HR-ESI-MS spectrum of natural **3.**

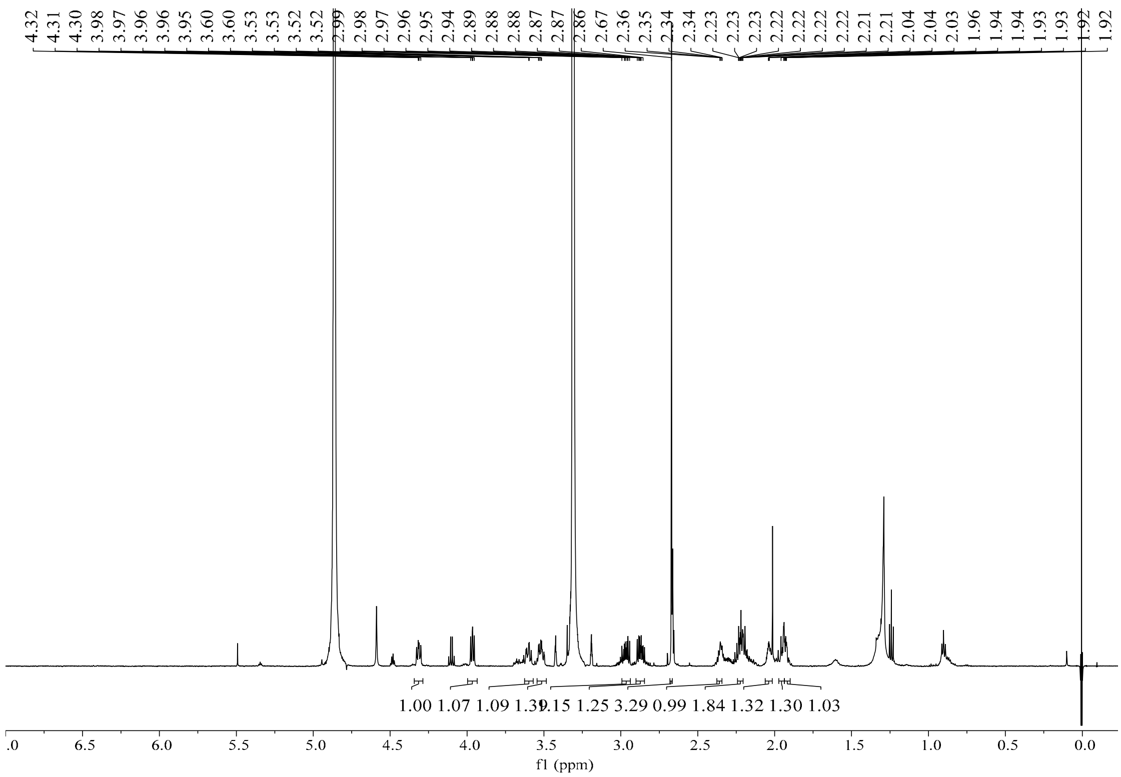

Fig. S30 ^{1}H NMR spectrum (600 MHz, CD_3OD) of natural **3.**

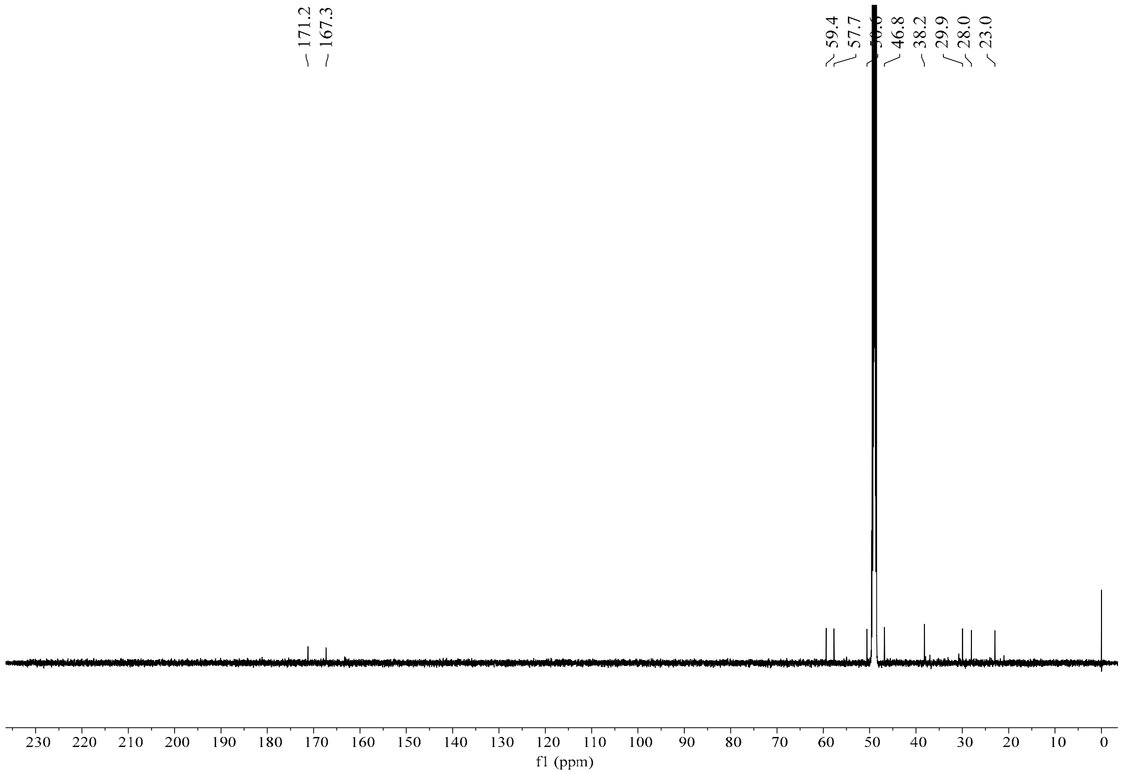

Fig. S31 ^{13}C NMR spectrum (150 MHz, CD_3OD) of natural **3.**

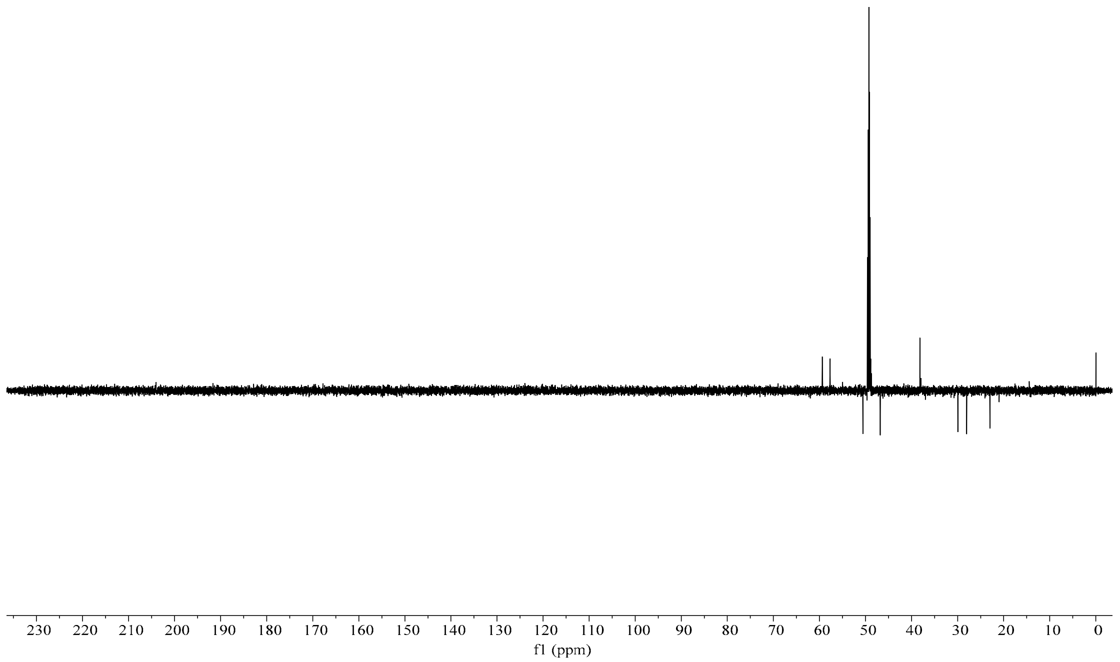

Fig. S32 DEPT-135 spectrum of natural **3.**

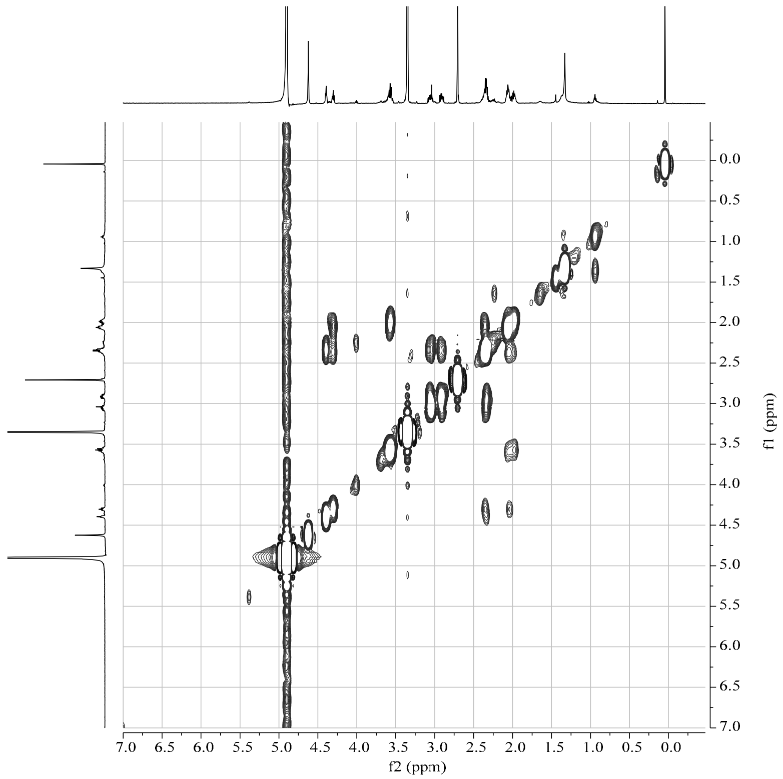

Fig. S33 ^{1}H-^{1}H COSY spectrum of natural **3.**

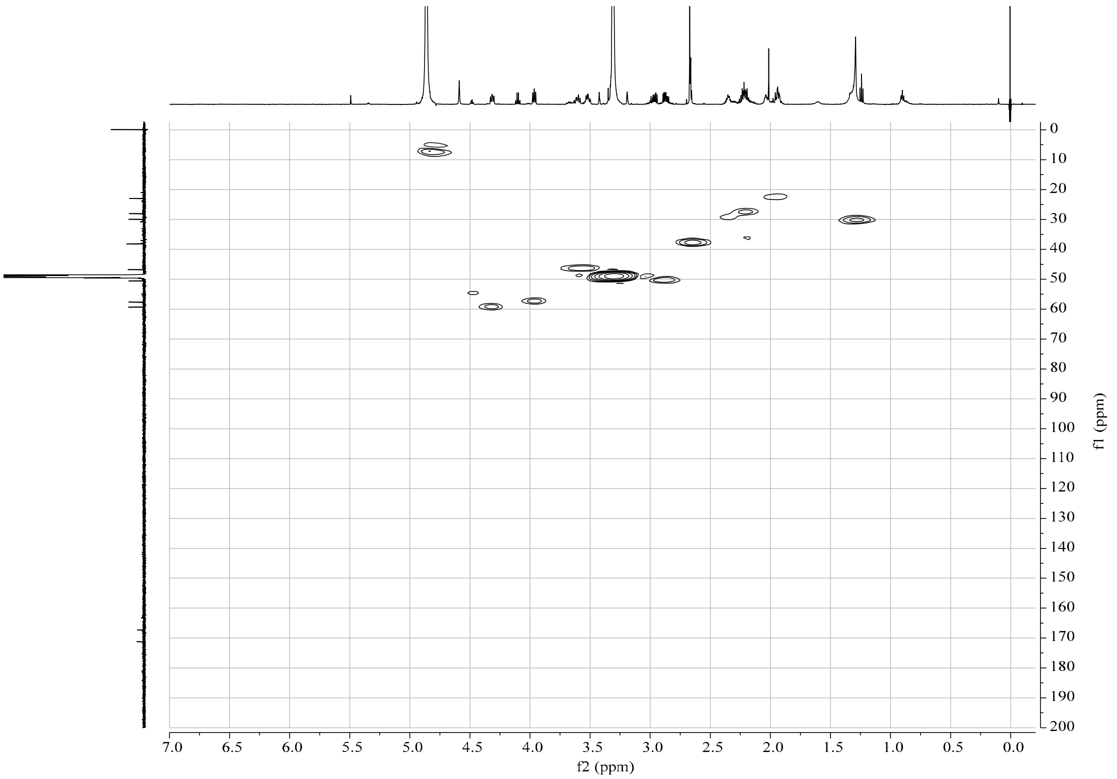

Fig. S34 HSQC spectrum of natural **3.**

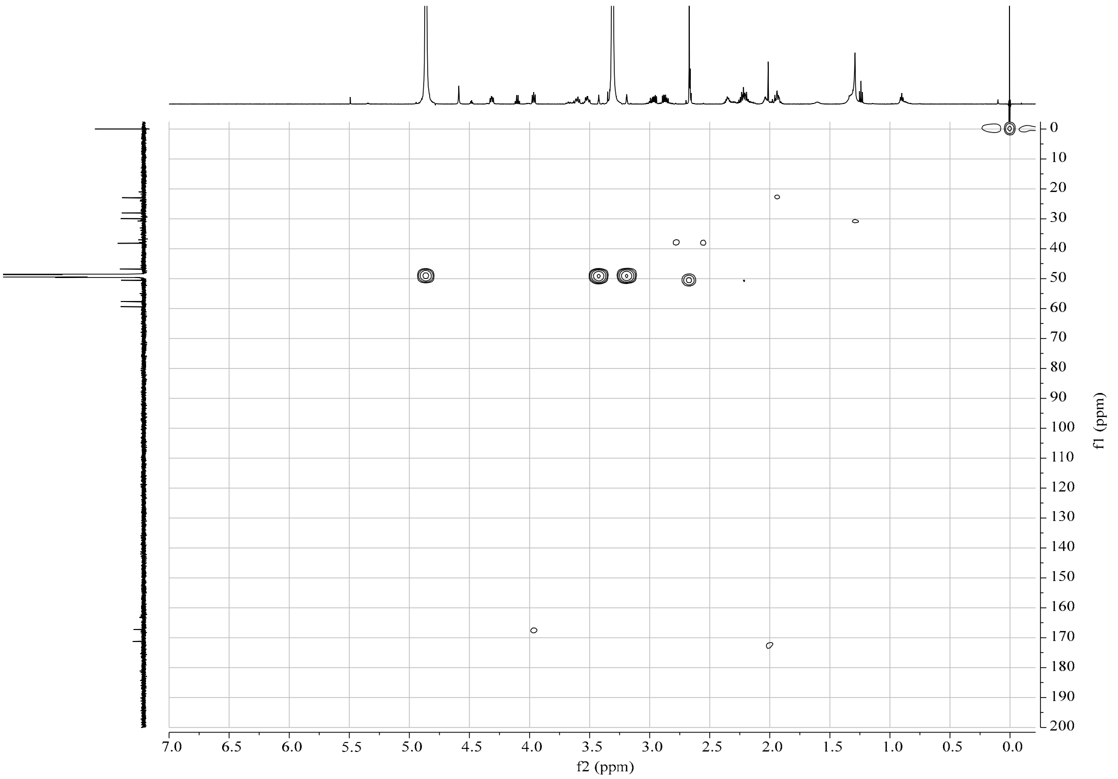

Fig. S35 HMBC spectrum of natural **3.**

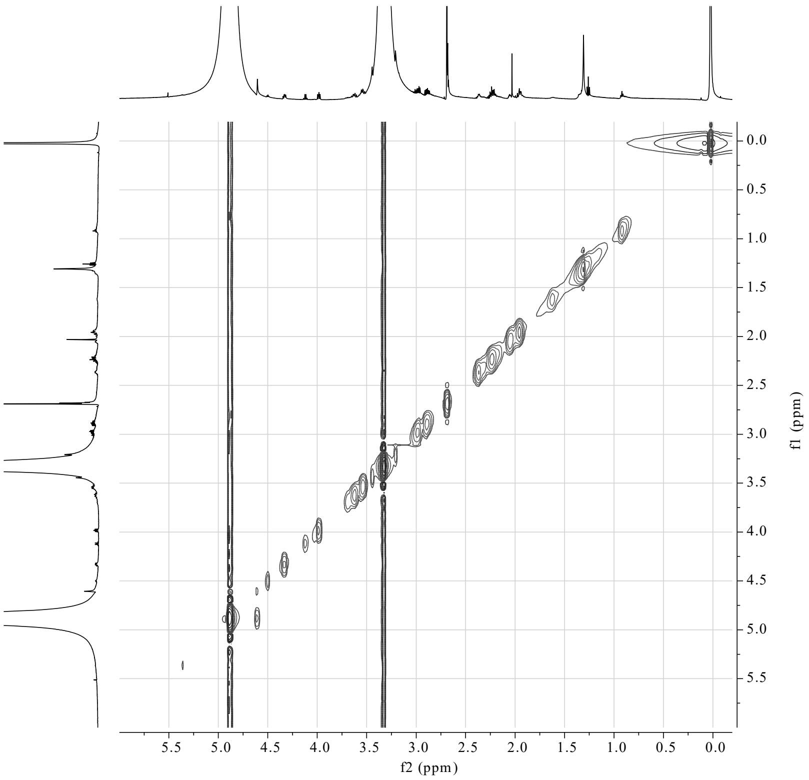

Fig. S36 NOESY spectrum of natural **3.**

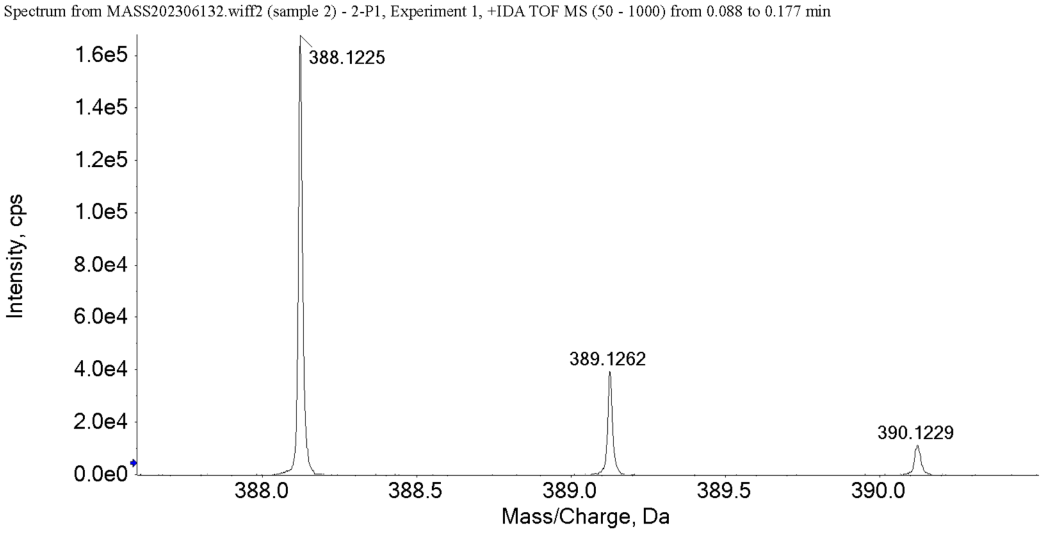

Hit	Formula	m/z	RDB	ppm	MS Rank	MSMS ppm	MSMS Rank	Found
1	$C_{20}H_{21}NO_5S$	388.1213	11.0	3.0	1			NA/NA

Fig. S37 HR-ESI-MS spectrum of **(*S*)-Fmoc-*L*-MetO.**

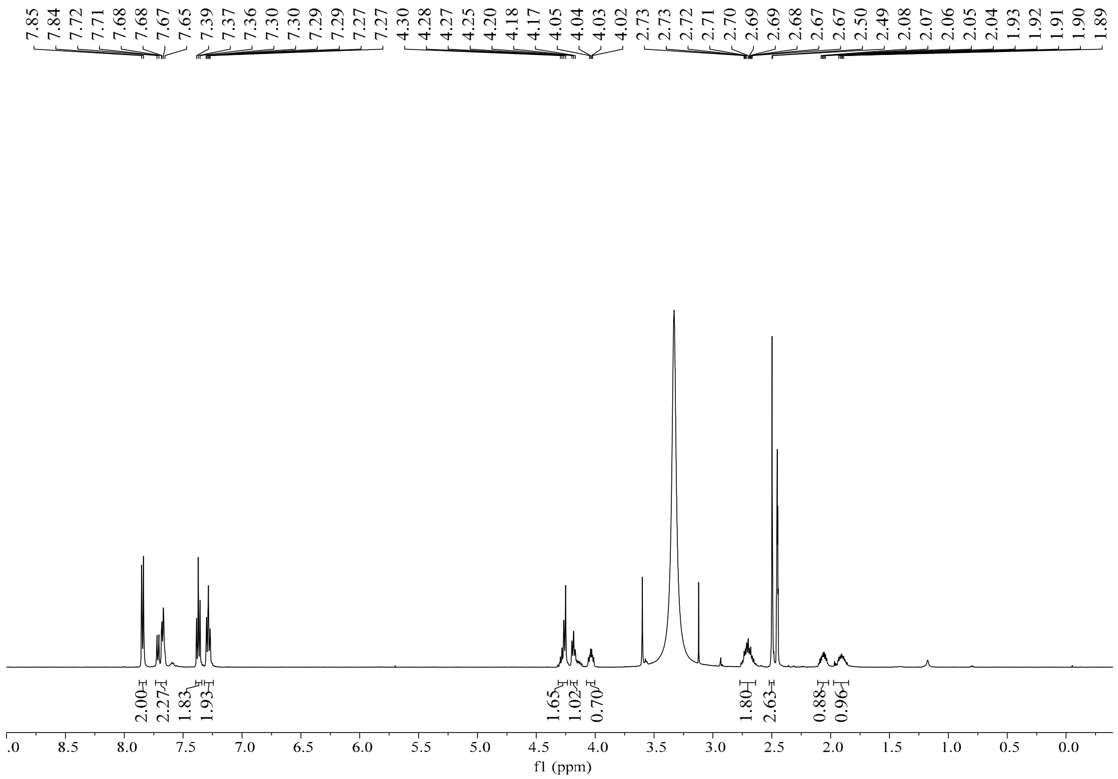

Fig. S38 ^{1}H NMR spectrum (600 MHz, DMSO-d_6) of **(*S*)-Fmoc-*L*-MetO.**

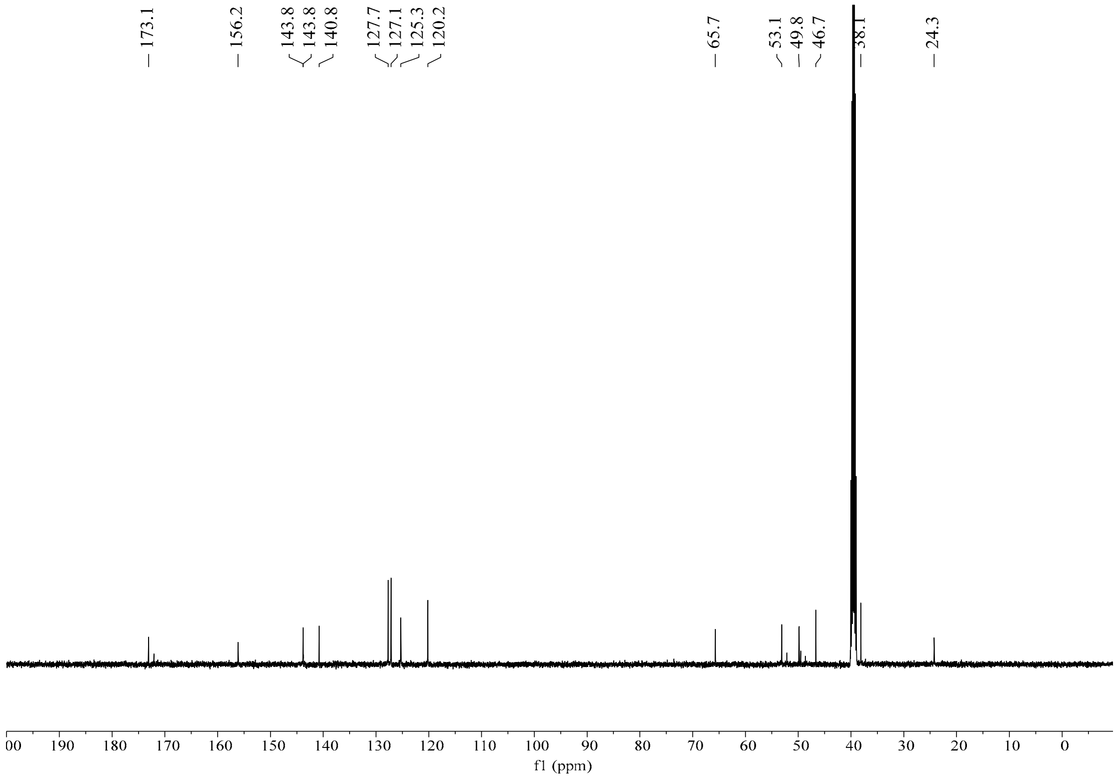

Fig. S39 ^{13}C NMR spectrum (150 MHz, DMSO-d_6) of **(*S*)-Fmoc-*L*-MetO.**

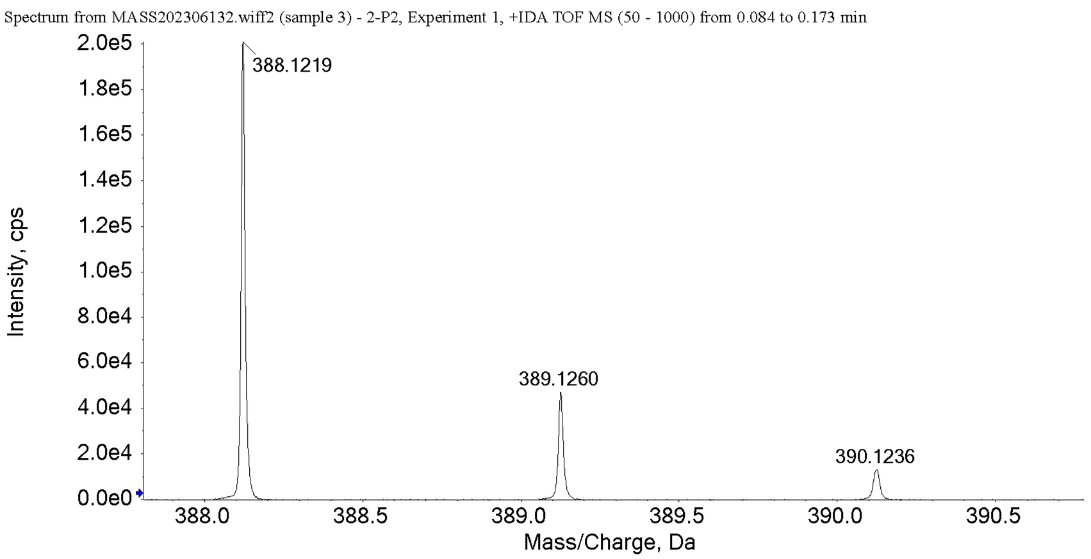

Hit	Formula	m/z	RDB	ppm	MS Rank	MSMS ppm	MSMS Rank	Found
1	$C_{20}H_{21}NO_5S$	388.1213	11.0	1.5	1			NA/NA

Fig. S40 HR-ESI-MS spectrum of **(*R*)-Fmoc-*L*-MetO.**

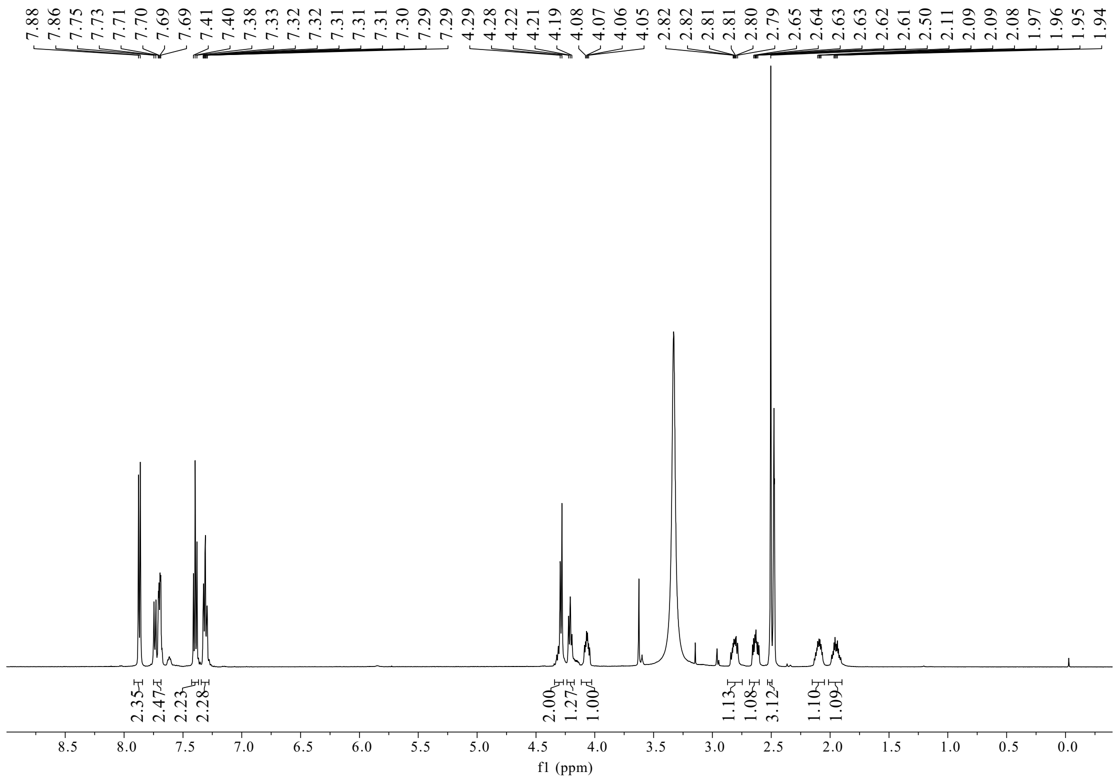

Fig. S41 ^{1}H NMR spectrum (600 MHz, DMSO-d_6) of **(*R*)-Fmoc-*L*-MetO.**

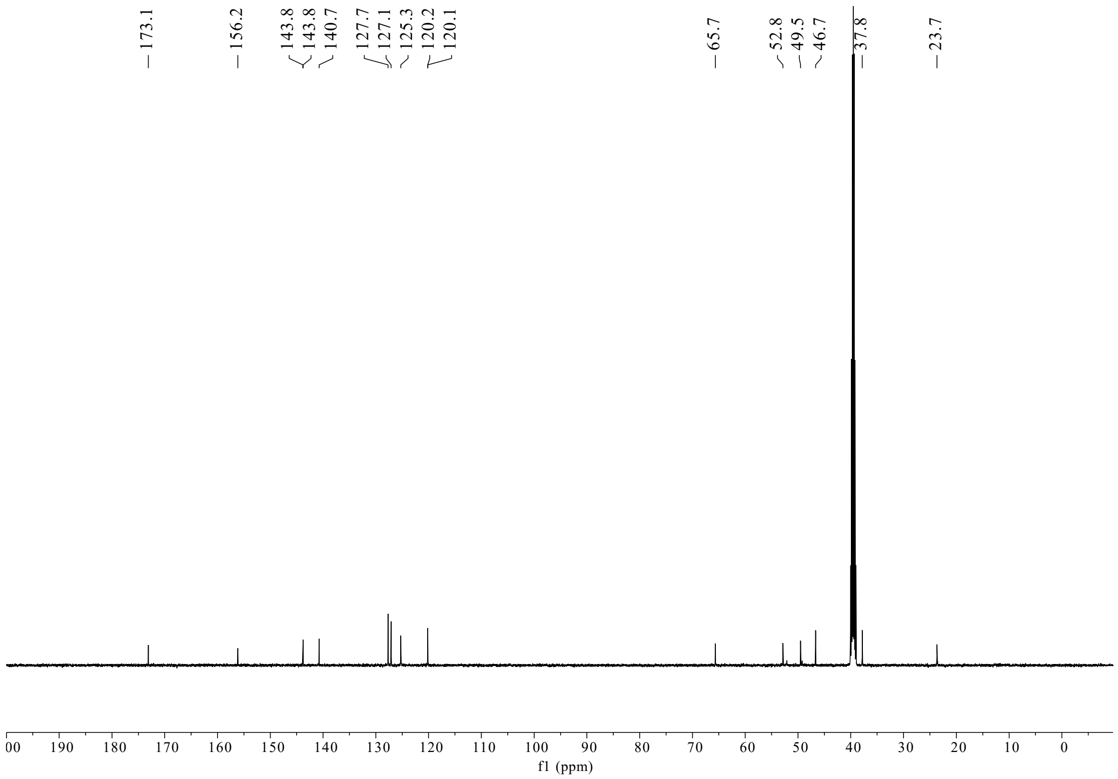

Fig. S42 ^{13}C NMR spectrum (150 MHz, DMSO-d_6) of **(*R*)-Fmoc-*L*-MetO.**

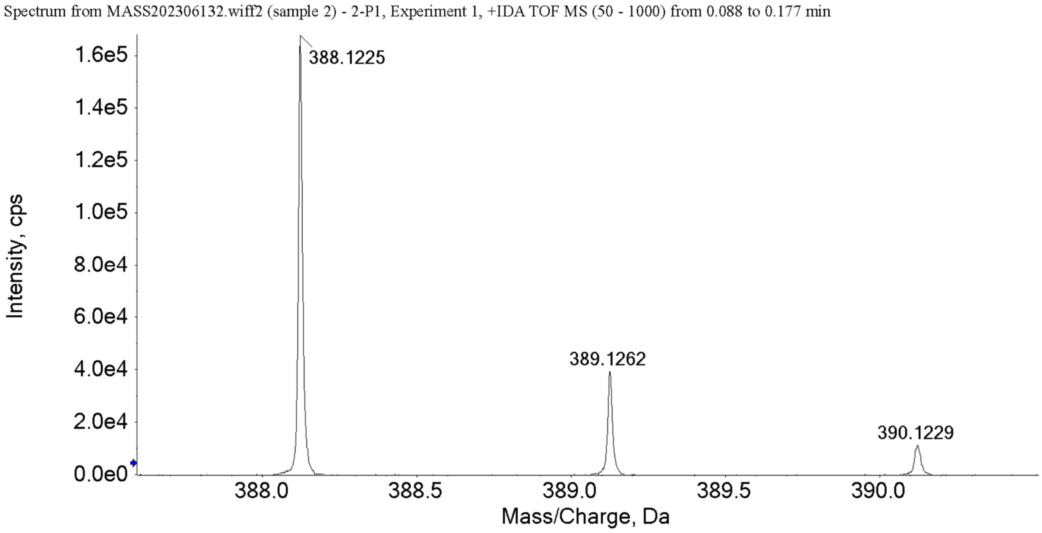

Hit	Formula	m/z	RDB	ppm	MS Rank	MSMS ppm	MSMS Rank	Found
1	$C_{20}H_{21}NO_5S$	388.1213	11.0	3.0	1			NA/NA

Fig. S43 HR-ESI-MS spectrum of **(*S*)-Fmoc-*D*-MetO.**

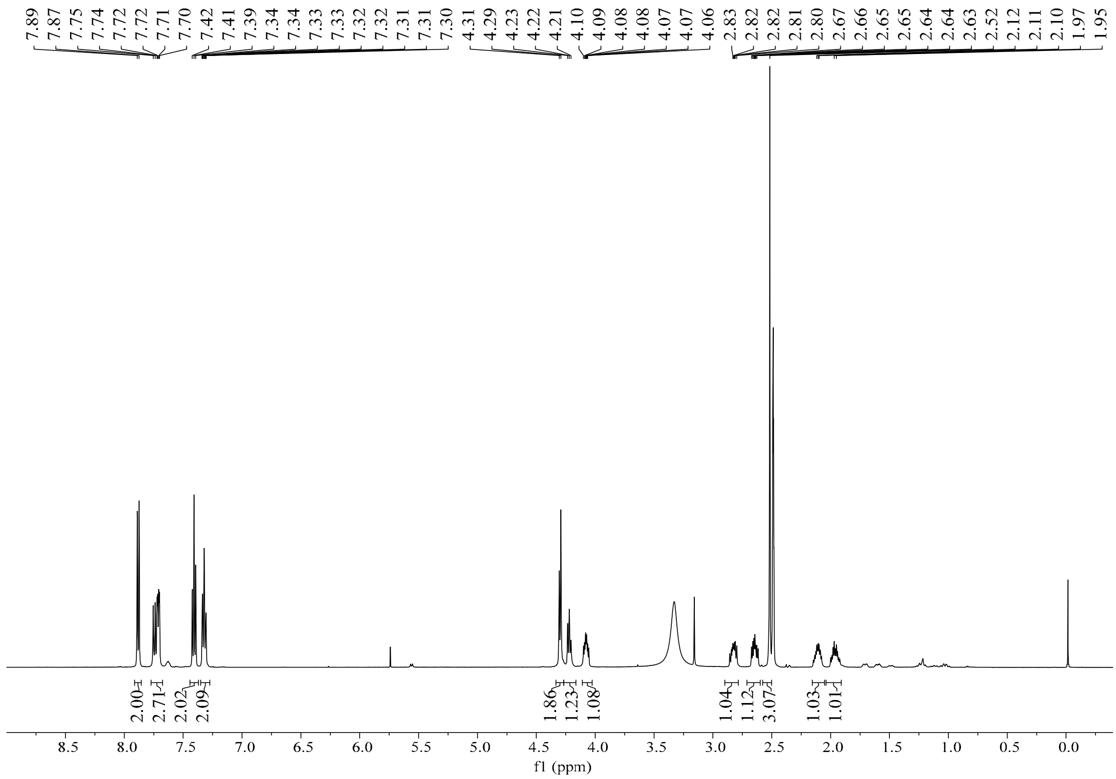

Fig. S44 ^{1}H NMR spectrum (600 MHz, DMSO-d_6) of **(*S*)-Fmoc-*D*-MetO.**

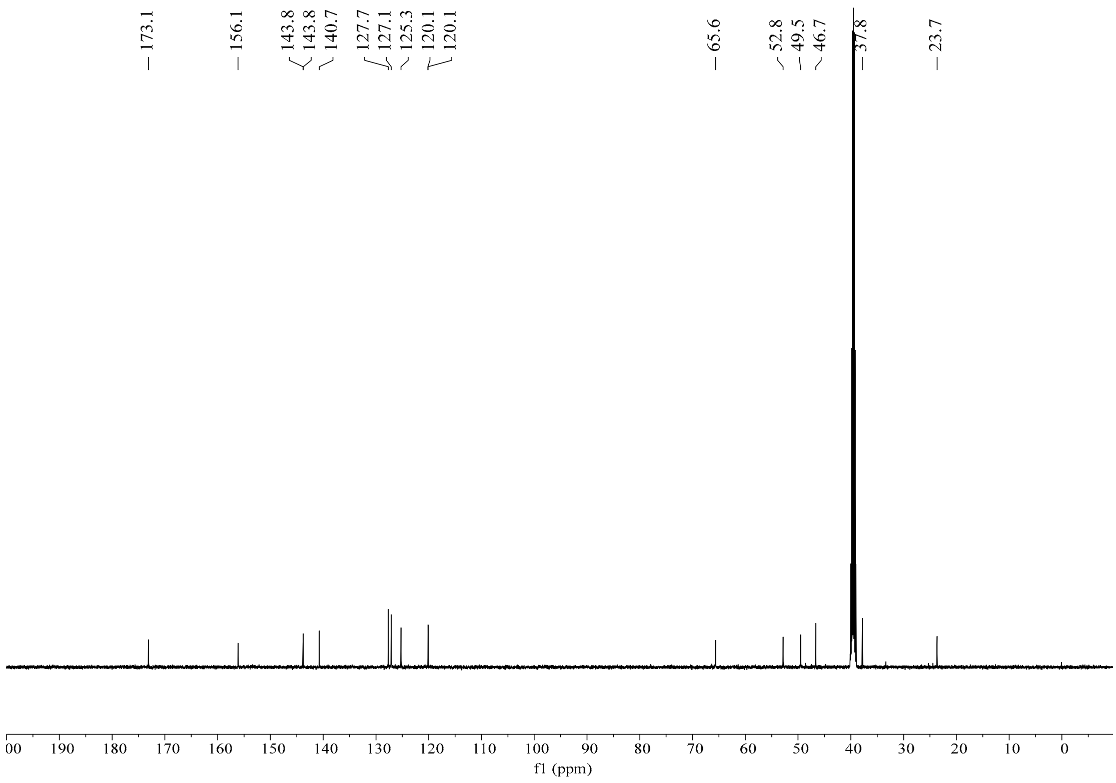

Fig. S45 ^{13}C NMR spectrum (150 MHz, DMSO-d_6) of **(*S*)-Fmoc-*D*-MetO.**

Spectrum from MASS202306132.wiff2 (sample 2) - 2-P1, Experiment 1, +IDA TOF MS (50 - 1000) from 0.088 to 0.177 min

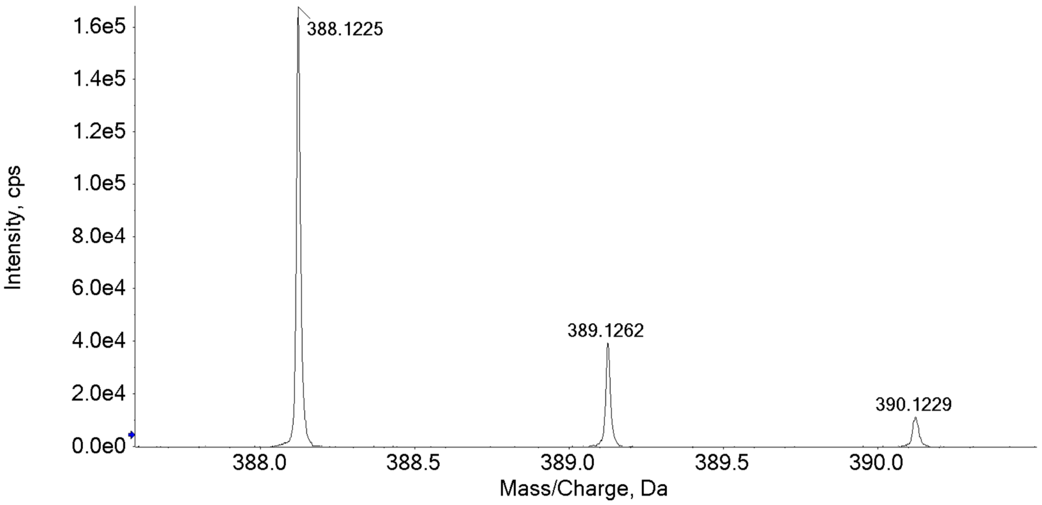

Hit	Formula	m/z	RDB	ppm	MS Rank	MSMS ppm	MSMS Rank	Found
1	$C_{20}H_{21}NO_5S$	388.1213	11.0	3.0	1			NA/NA

Fig. S46 HR-ESI-MS spectrum of **(*R*)-Fmoc-*D*-MetO.**

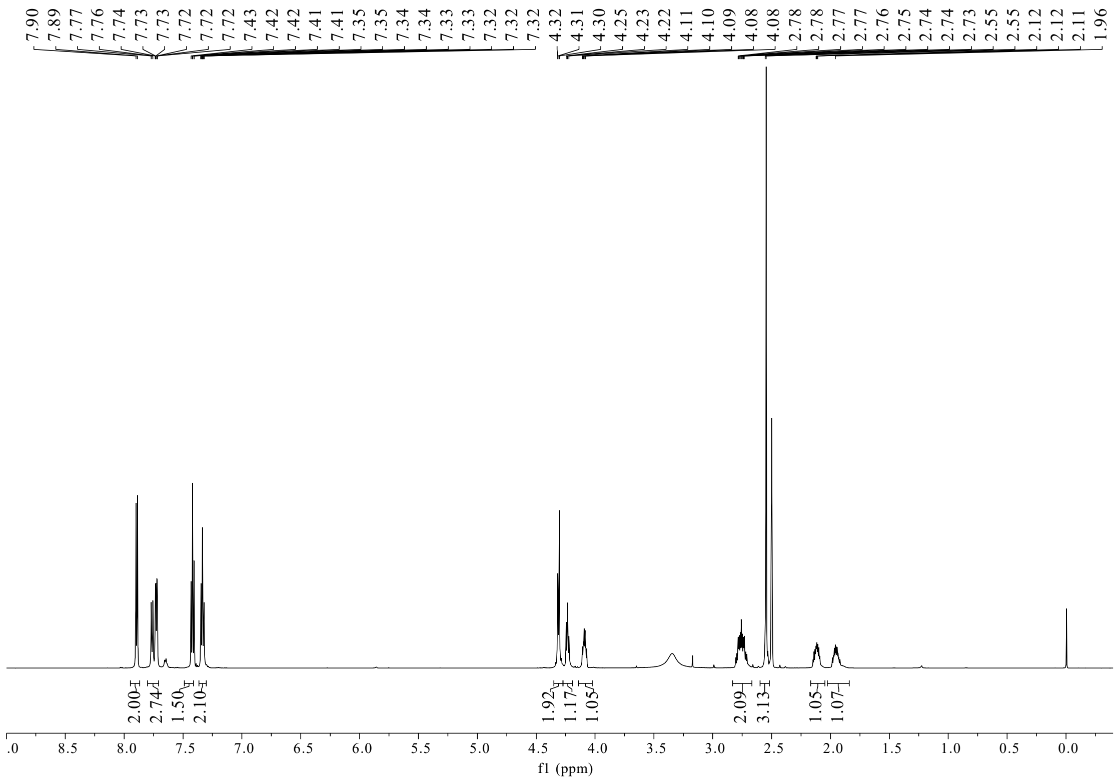

Fig. S47 ^{1}H NMR spectrum (600 MHz, DMSO-*d*6) of **(*R*)-Fmoc-*D*-MetO.**

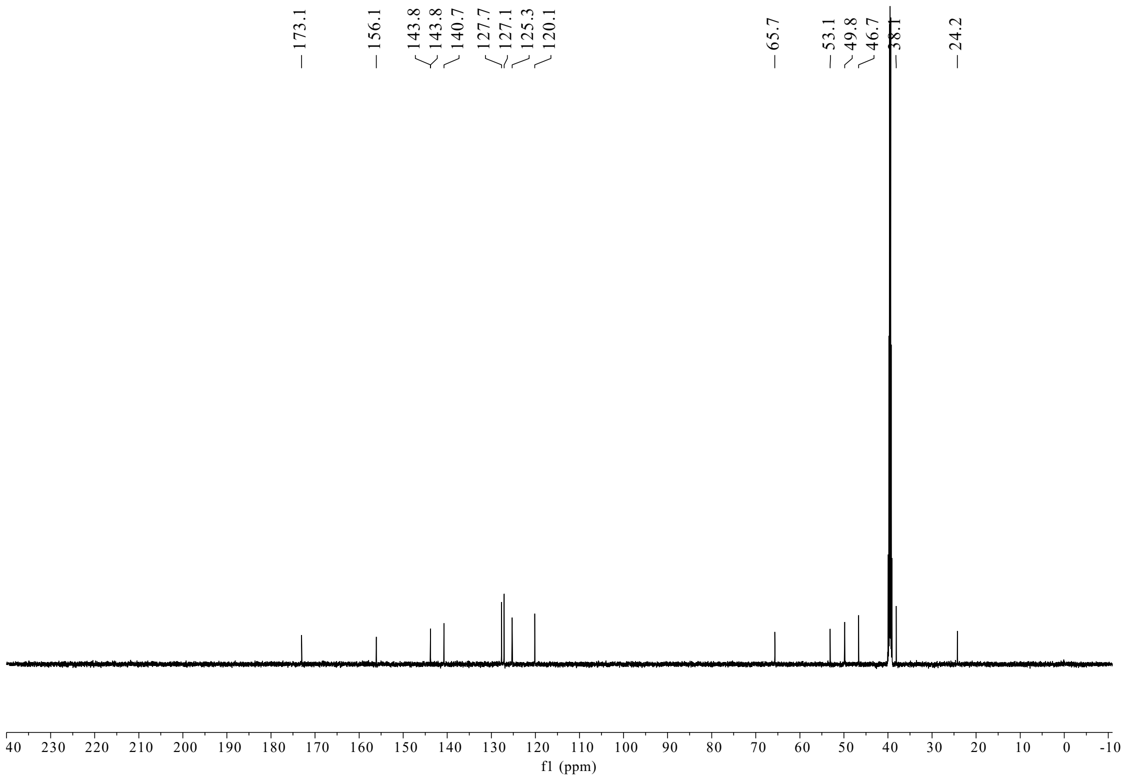

Fig. S48 ^{13}C NMR spectrum (150 MHz, DMSO-d_6) of **(*R*)-Fmoc-*D*-MetO.**

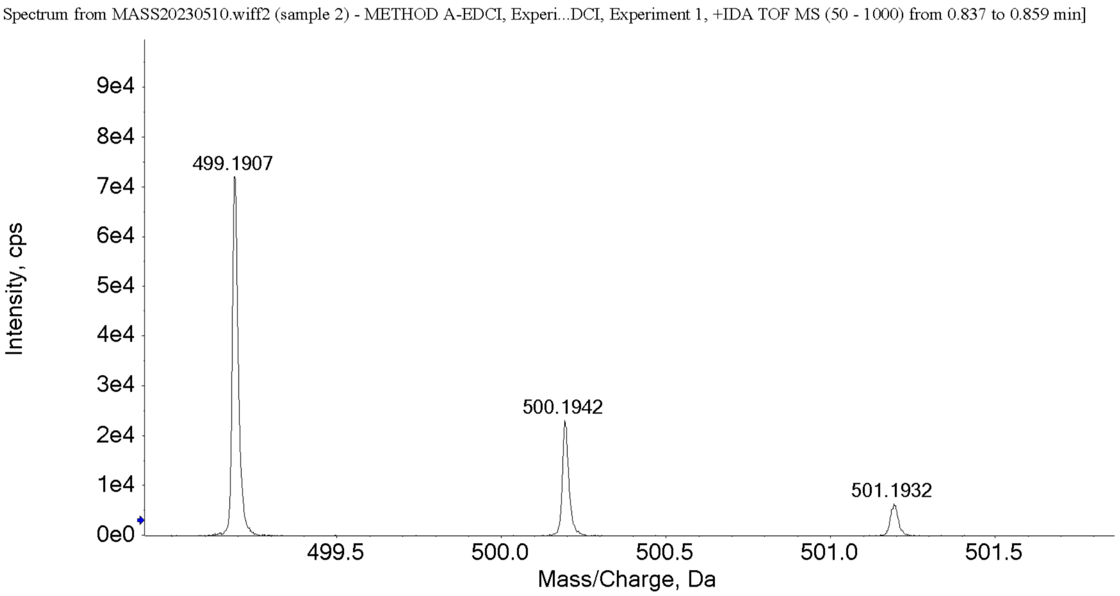

Hit	Formula	m/z	RDB	ppm	MS Rank	MSMS ppm	MSMS Rank	Found
1	$C_{26}H_{30}N_2O_6S$	499.1897	13.0	3.1	1			NA/NA

Fig. S49 HR-ESI-MS spectrum of **A1.**

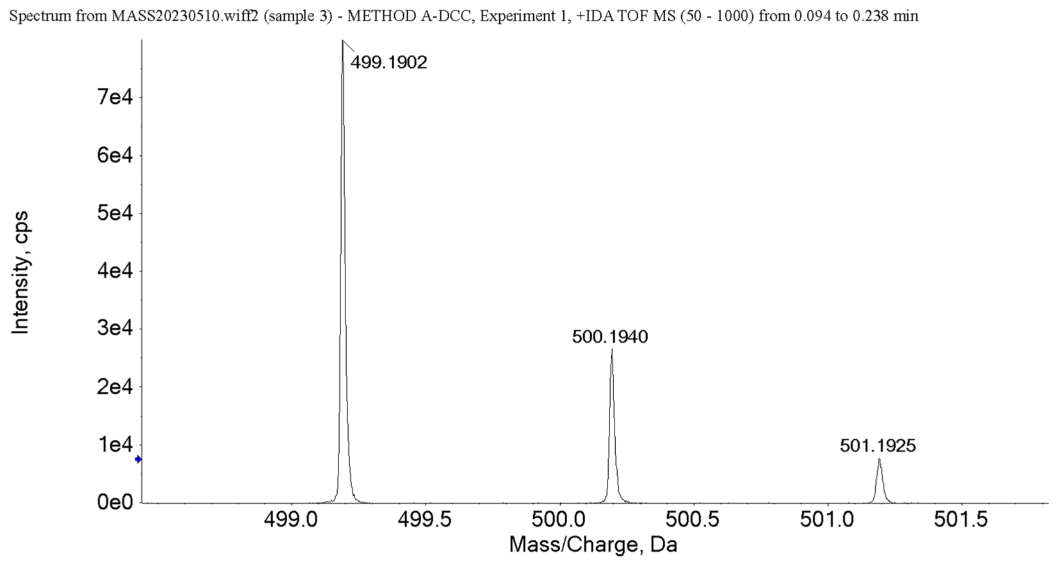

Hit	Formula	m/z	RDB	ppm	MS Rank	MSMS ppm	MSMS Rank	Found
1	$C_{26}H_{30}N_2O_6S$	499.1897	13.0	0.9	1			NA/NA

Fig. S50 HR-ESI-MS spectrum of **B1.**

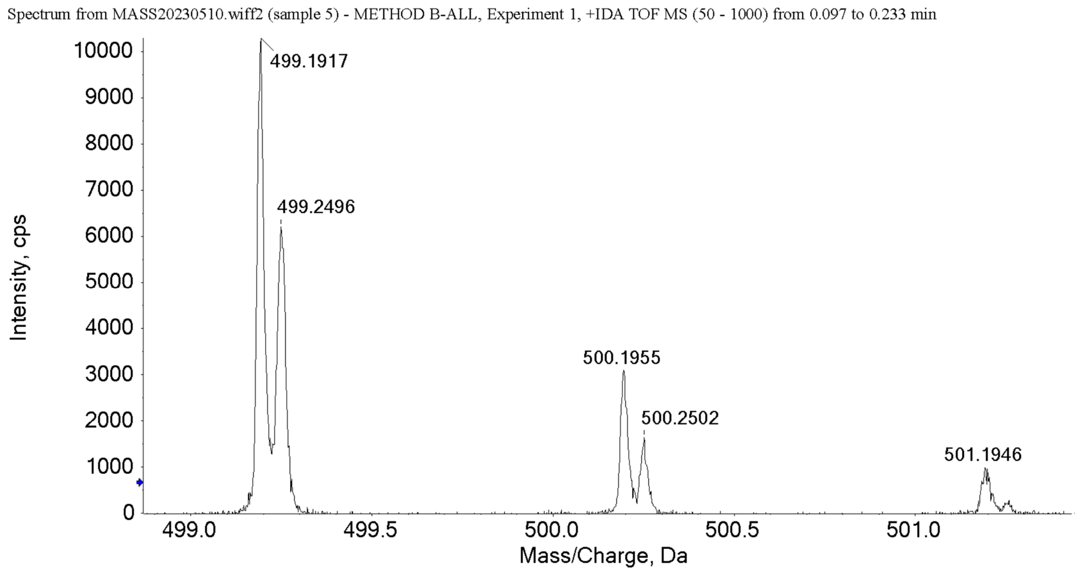

Hit	Formula	m/z	RDB	ppm	MS Rank	MSMS ppm	MSMS Rank	Found
1	$C_{26}H_{30}N_2O_6S$	499.1897	13.0	3.9	1			NA/NA

Fig. S51 HR-ESI-MS spectrum of **C1.**

Spectrum from MASS20230625.wiff2 (sample 5) - G2, Experiment 1, +IDA TOF MS (50 - 1000) from 0.049 to 0.094 min

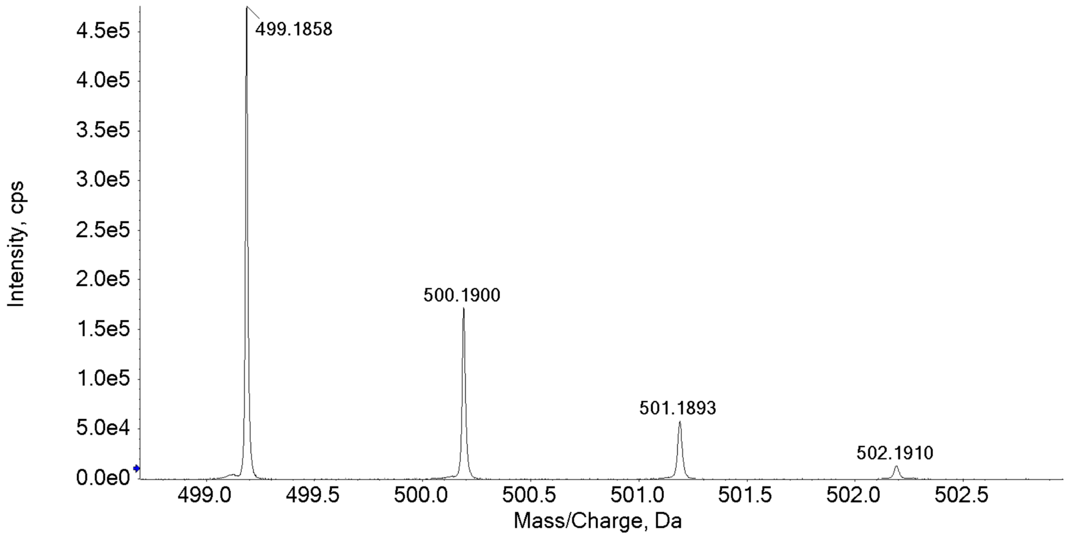

Hit	Formula	m/z	RDB	ppm	MS Rank	MSMS ppm	MSMS Rank	Found
1	$C_{26}H_{30}N_2O_6S$	499.1897	13.0	-7.9	1			NA/NA

Fig. S52 HR-ESI-MS spectrum of **D1.**

Spectrum from mass20230515.wiff2 (sample 41) - STEP3, Experiment 1, +I... STEP3, Experiment 1, +IDA TOF MS (50 - 1000) from 0.723 to 0.743 min]

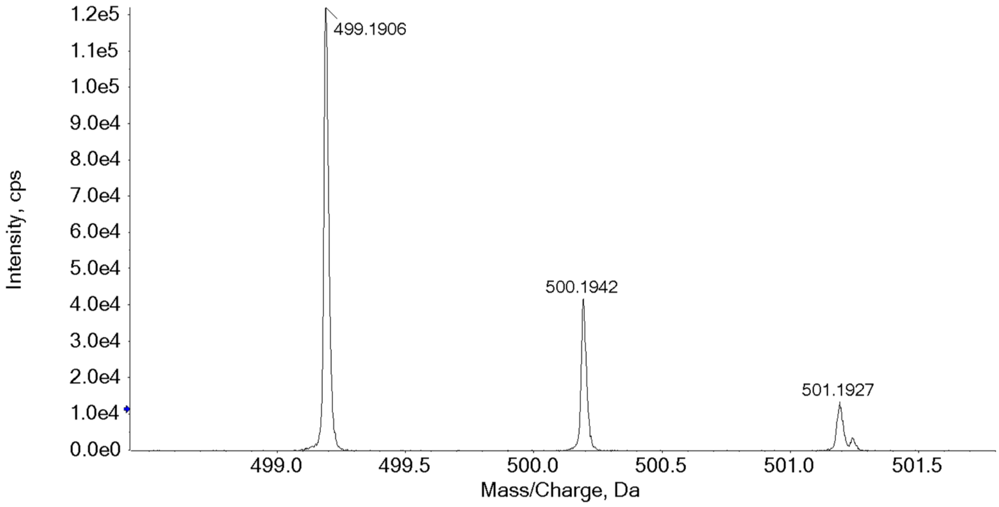

Hit	Formula	m/z	RDB	ppm	MS Rank	MSMS ppm	MSMS Rank	Found
1	$C_{26}H_{30}N_2O_6S$	499.1897	13.0	1.7	1			NA/NA

Fig. S53 HR-ESI-MS spectrum of **E1.**

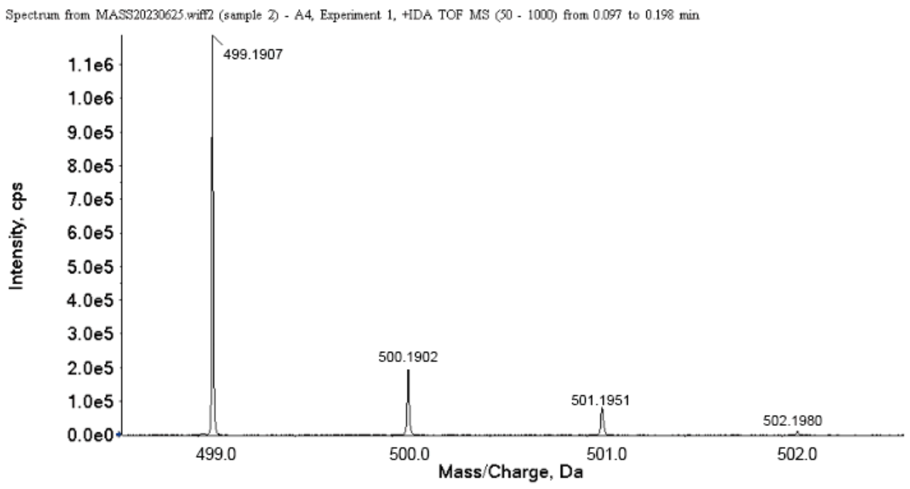

Hit	Formula	m/z	RDB	ppm	MS Rank	MSMS ppm	MSMS Rank	Found
1	$C_{26}H_{30}N_2O_6S$	499.1897	13.0	3.1	1			NA/NA

Fig. S54 HR-ESI-MS spectrum of **F1.**

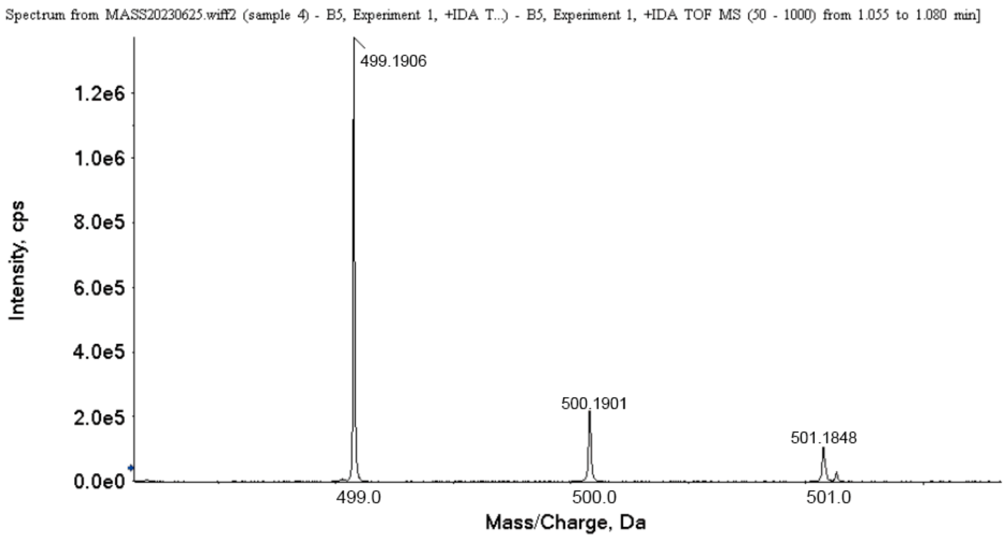

Hit	Formula	m/z	RDB	ppm	MS Rank	MSMS ppm	MSMS Rank	Found
1	$C_{26}H_{30}N_2O_6S$	499.1897	13.0	1.7	1			NA/NA

Fig. S55 HR-ESI-MS spectrum of **G1.**

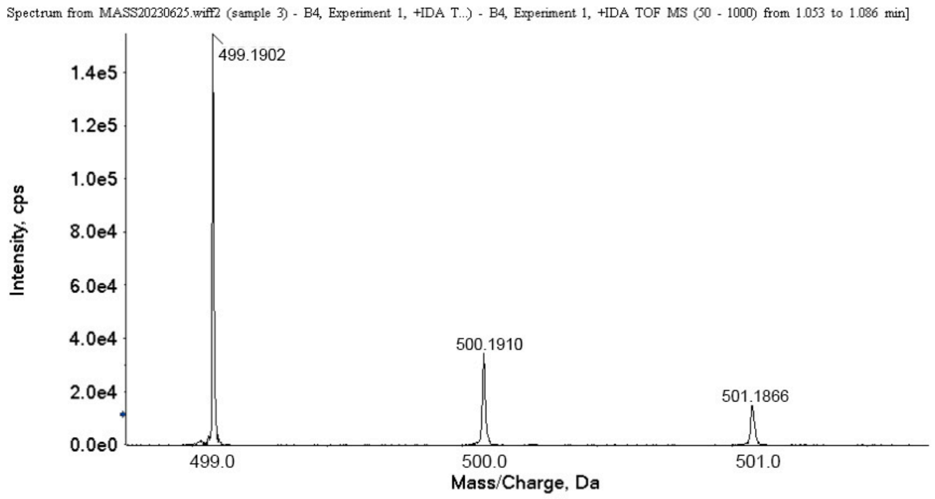

Hit	Formula	m/z	RDB	ppm	MS Rank	MSMS ppm	MSMS Rank	Found
1	$C_{26}H_{30}N_2O_6S$	499.1897	13.0	0.9	1			NA/NA

Fig. S56 HR-ESI-MS spectrum of **H1.**

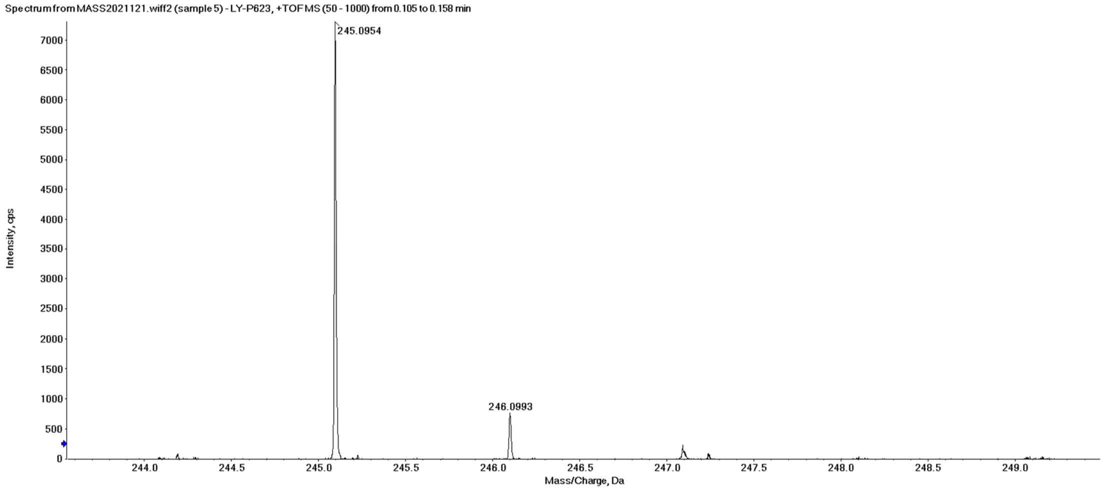

Hit	Formula	m/z	RDB	ppm	MS Rank	MSMS ppm	MSMS Rank	Found
1	$C_{10}H_{16}N_2O_3S$	245.0954	4.0	-0.2	1			NA/NA

Fig. S57 HR-ESI-MS spectrum of synthetic (+)-**1.**

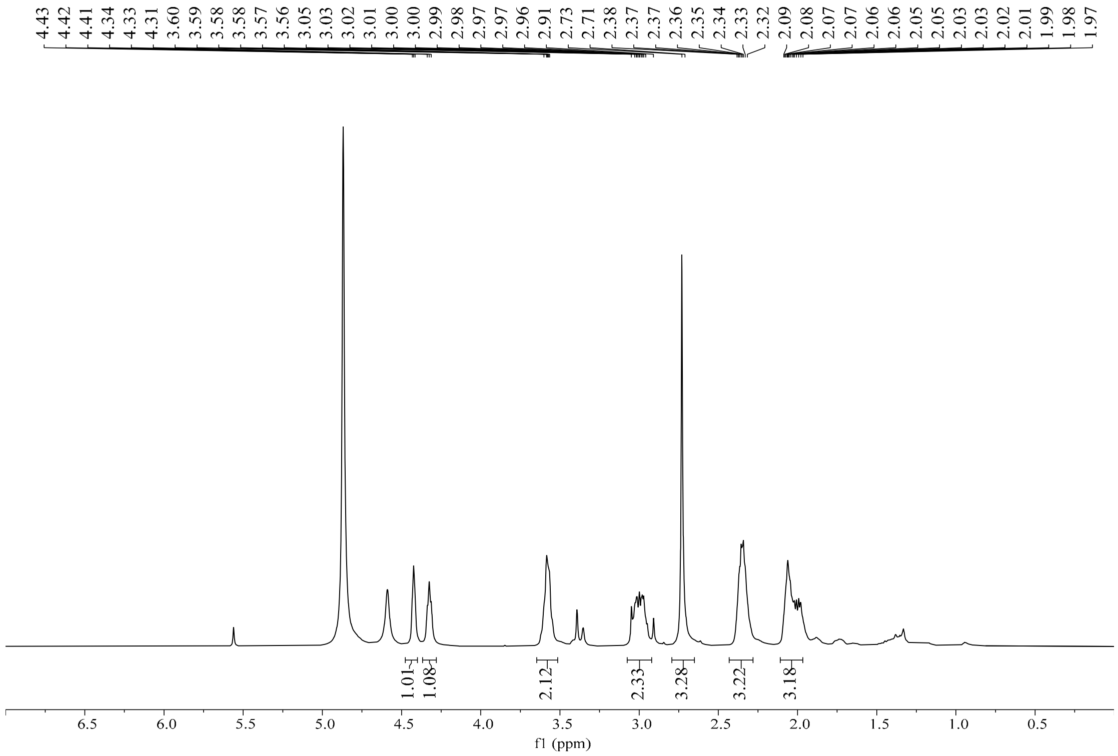

Fig. S58 ^{1}H NMR spectrum (600 MHz, CD_3OD) of synthetic (+)-**1.**

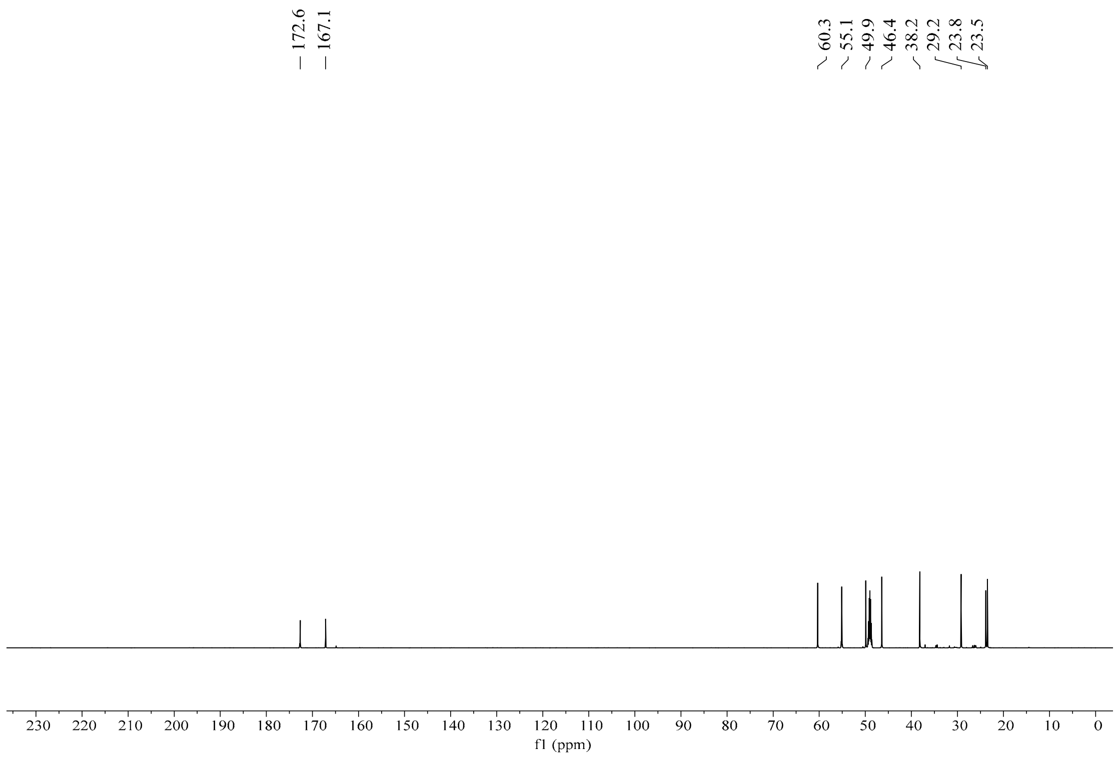

Fig. S59 ^{13}C NMR spectrum (150 MHz, CD_3OD) of synthetic (+)-**1.**

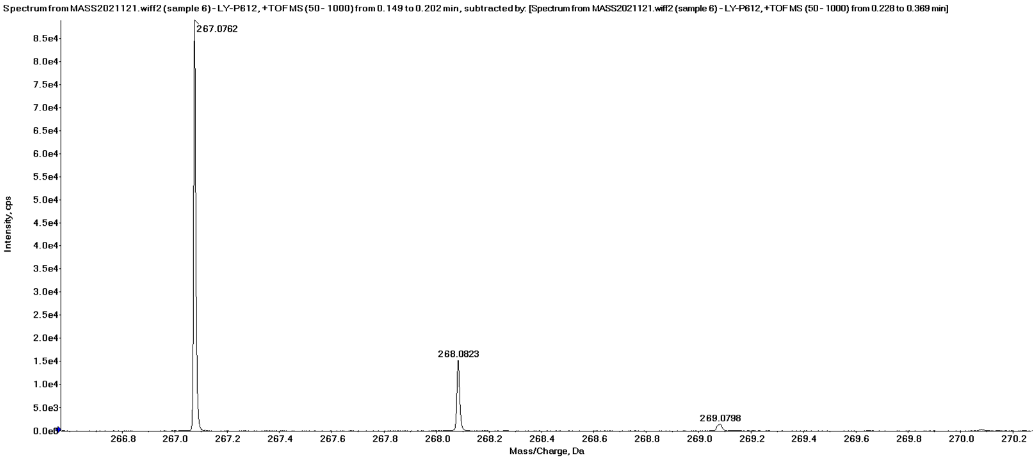

Hit	Formula	m/z	RDB	ppm	MS Rank	MSMS ppm	MSMS Rank	Found
1	$C_{10}H_{16}N_2O_3S$	267.0774	4.0	-4.4	1			NA/NA

Fig. S60 HR-ESI-MS spectrum of synthetic (-)-**1.**

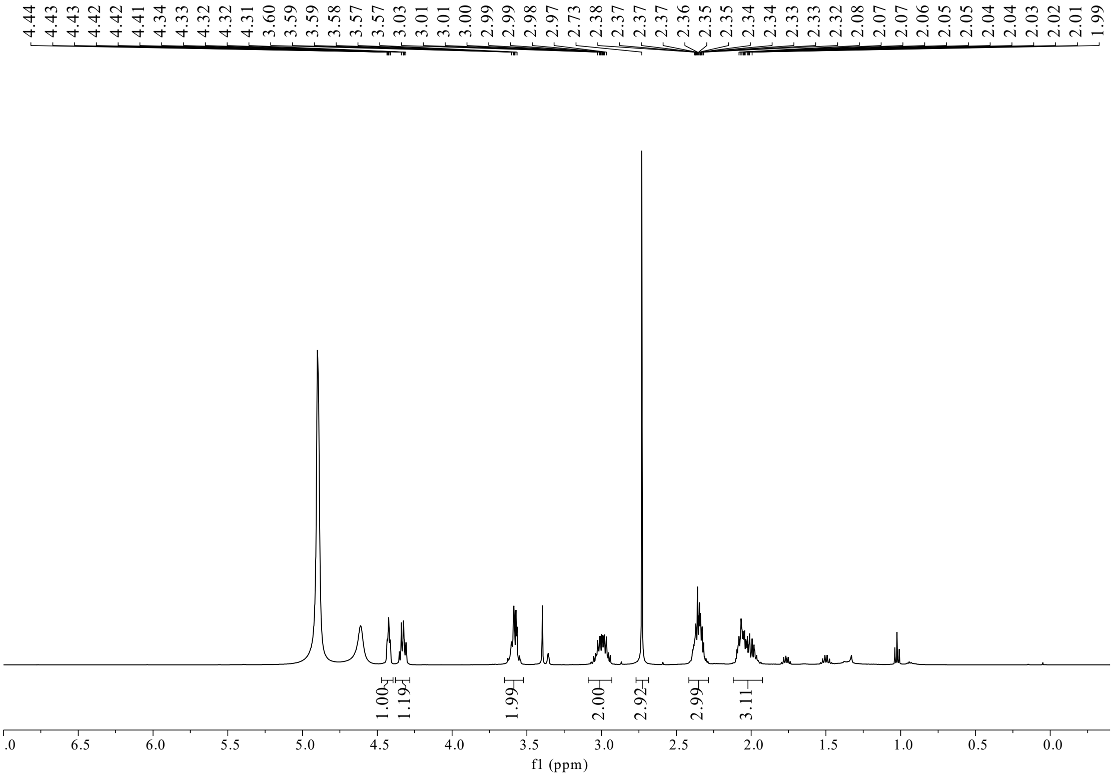

Fig. S61 ^{1}H NMR spectrum (600 MHz, CD_3OD) of synthetic (-)-**1.**

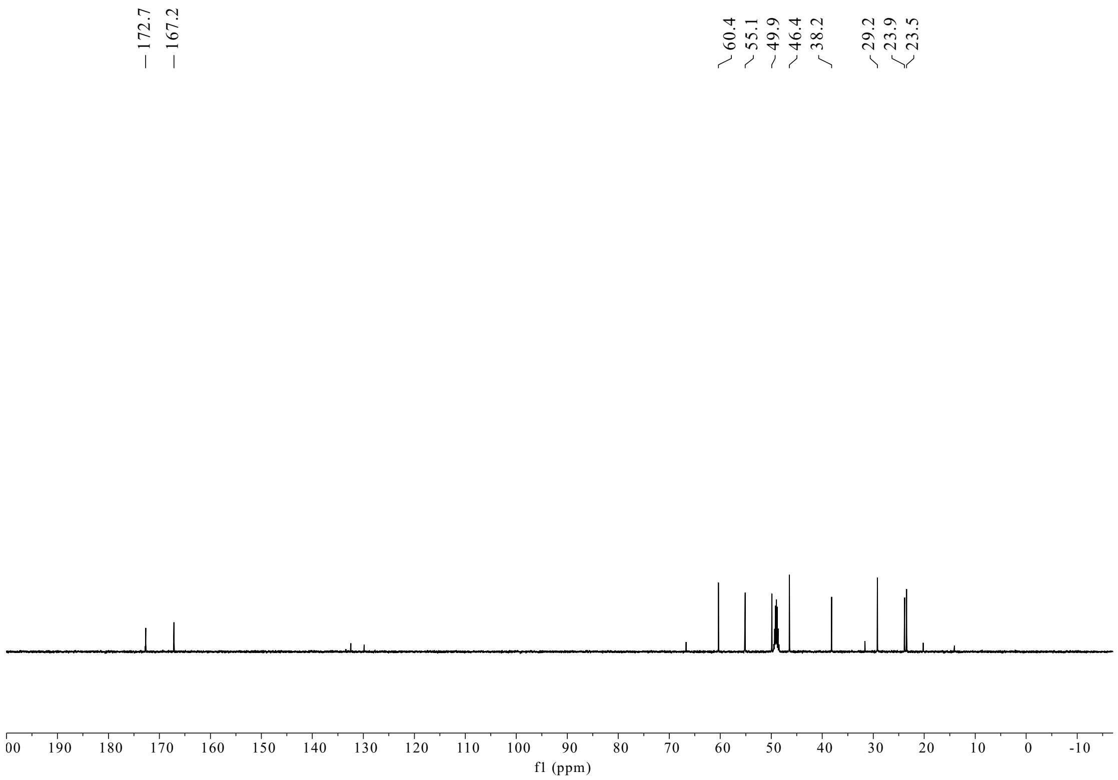

Fig. S62 ^{13}C NMR spectrum (150 MHz, CD_3OD) of synthetic (-)-**1.**

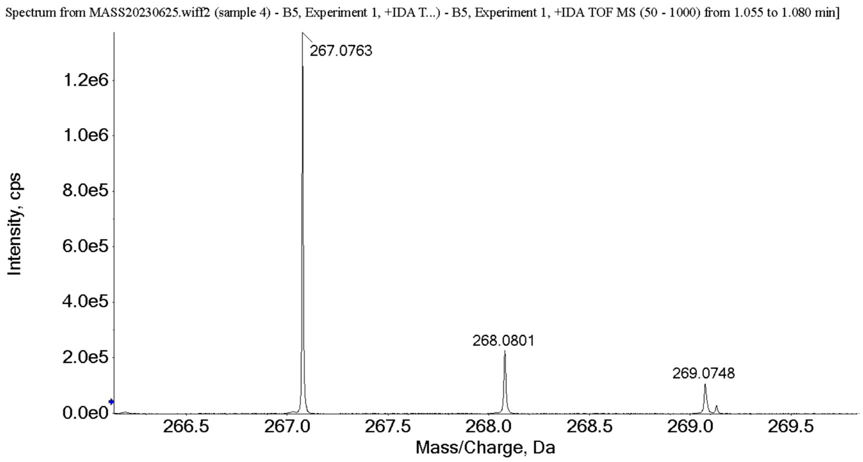

Hit	Formula	m/z	RDB	ppm	MS Rank	MSMS ppm	MSMS Rank	Found
1	$C_{10}H_{16}N_2O_3S$	267.0774	4.0	-4.1	1			NA/NA

Fig. S63 HR-ESI-MS spectrum of synthetic (+)-**2.**

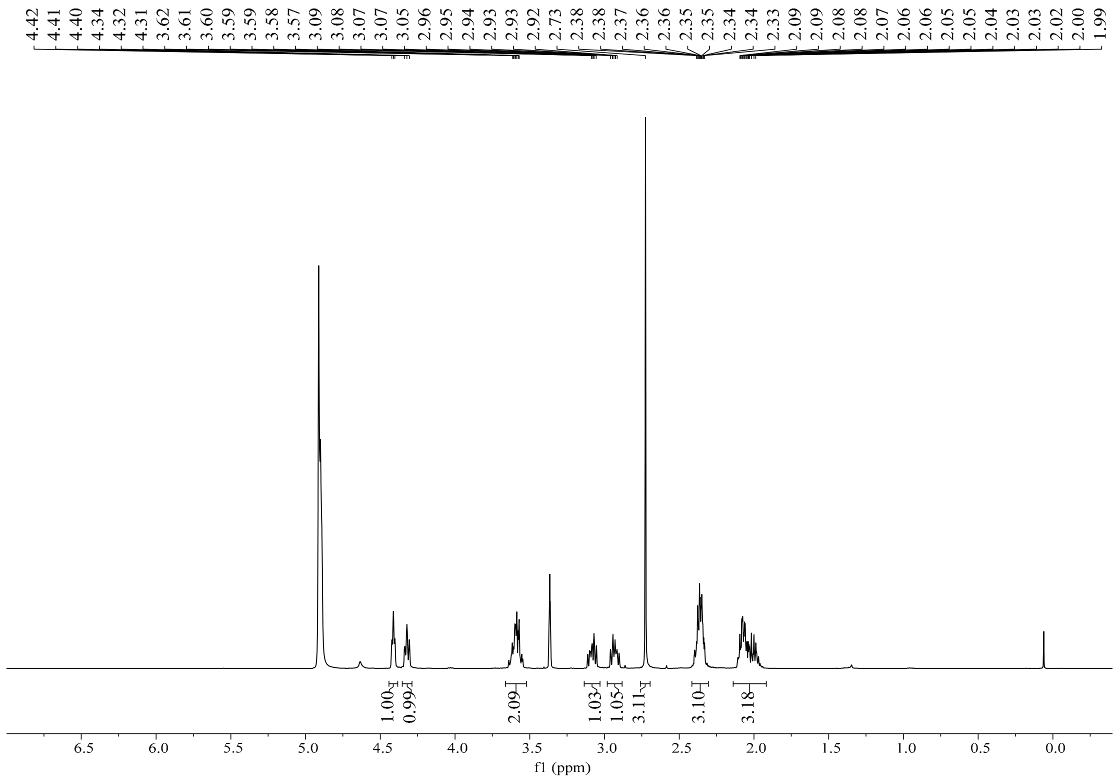

Fig. S64 ^{1}H NMR spectrum (600 MHz, CD3OD) of synthetic (+)-**2.**

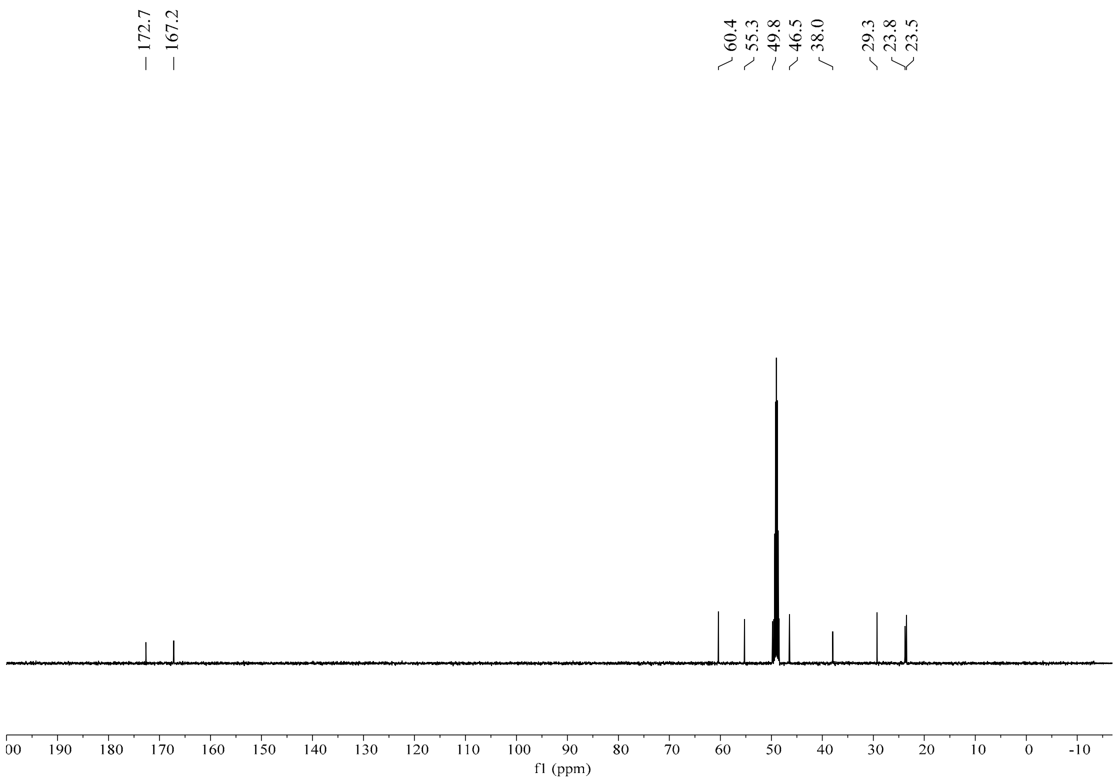

Fig. S65 ^{13}C NMR spectrum (150 MHz, CD3OD) of synthetic (+)-**2.**

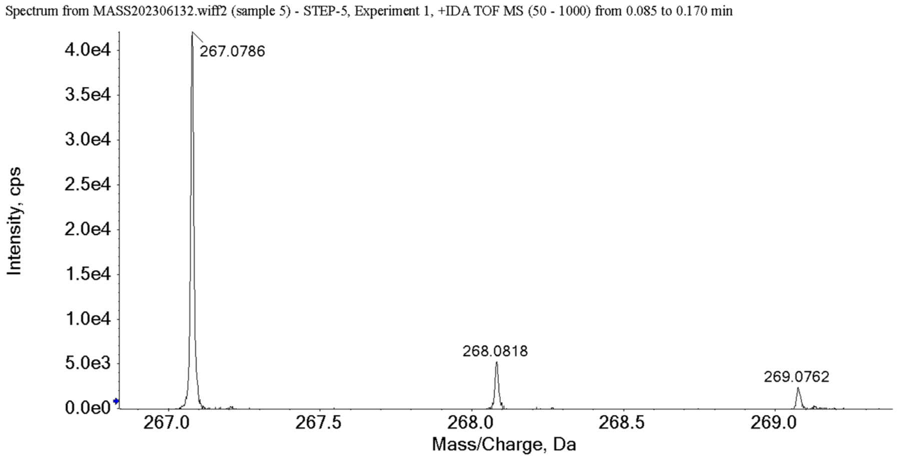

Hit	Formula	m/z	RDB	ppm	MS Rank	MSMS ppm	MSMS Rank	Found
1	$C_{10}H_{16}N_2O_3S$	267.0774	4.0	4.6	1			NA/NA

Fig. S66 HR-ESI-MS spectrum of synthetic (-)-**2.**

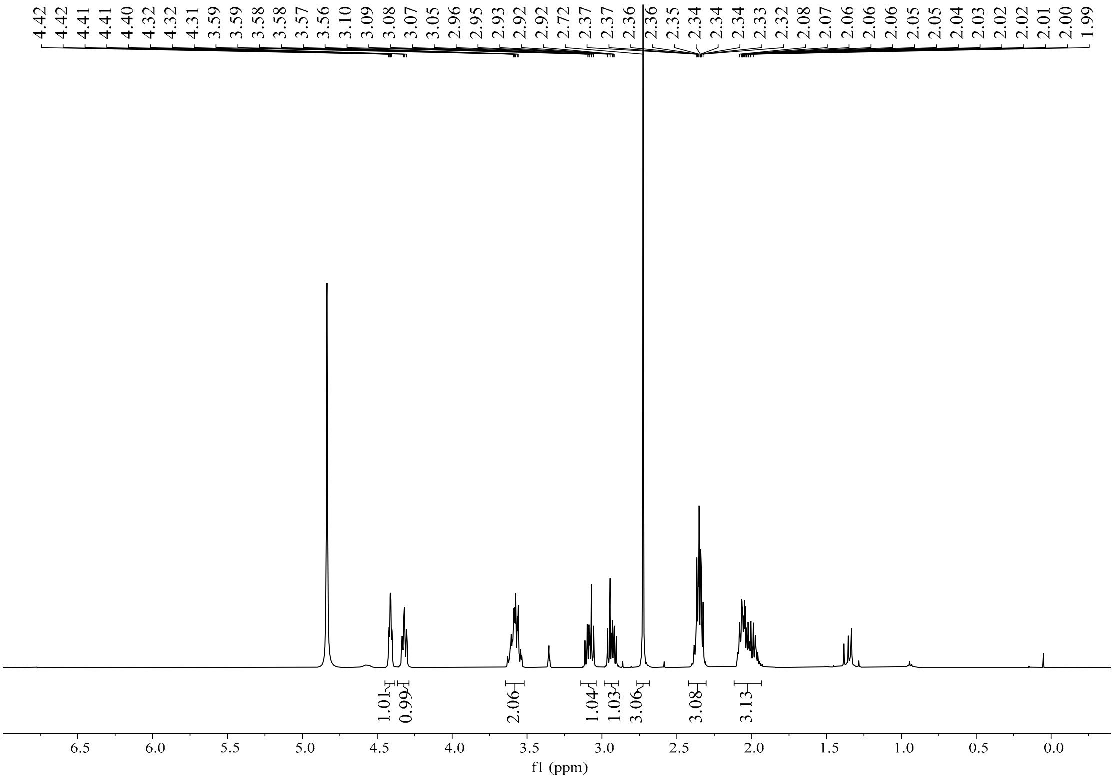

Fig. S67 ^{1}H NMR spectrum (600 MHz, CD_3OD) of synthetic (-)-**2.**

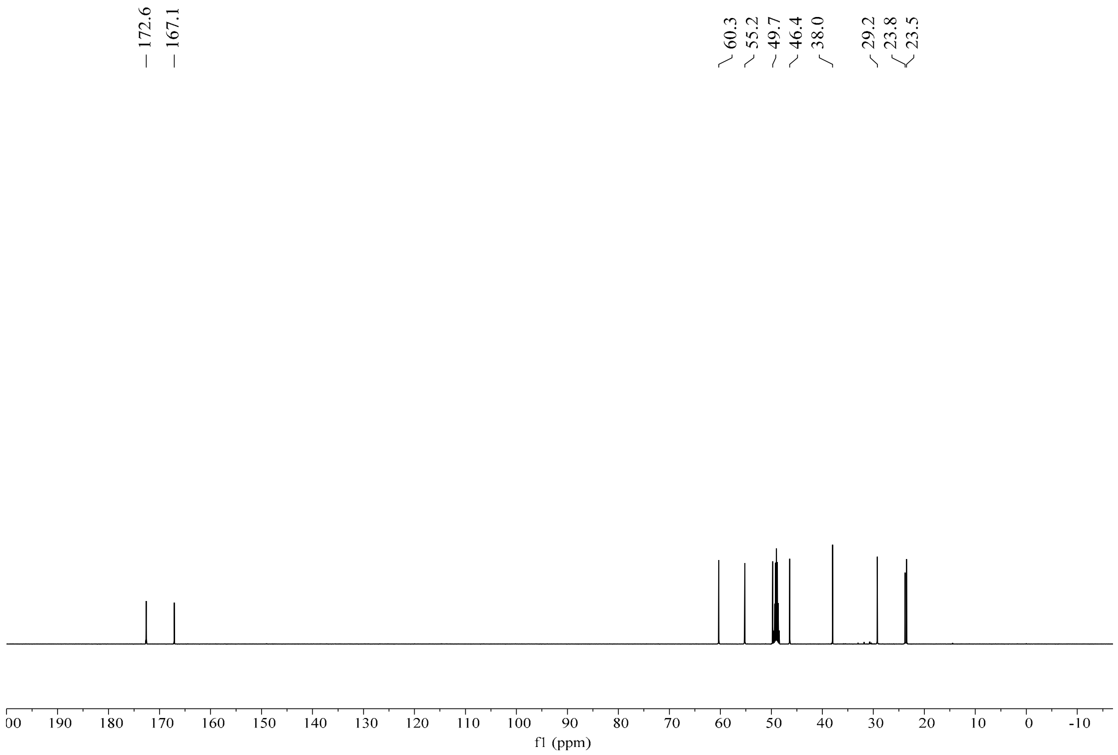

Fig. S68 ^{13}C NMR spectrum (150 MHz, CD_3OD) of synthetic (-)-**2.**

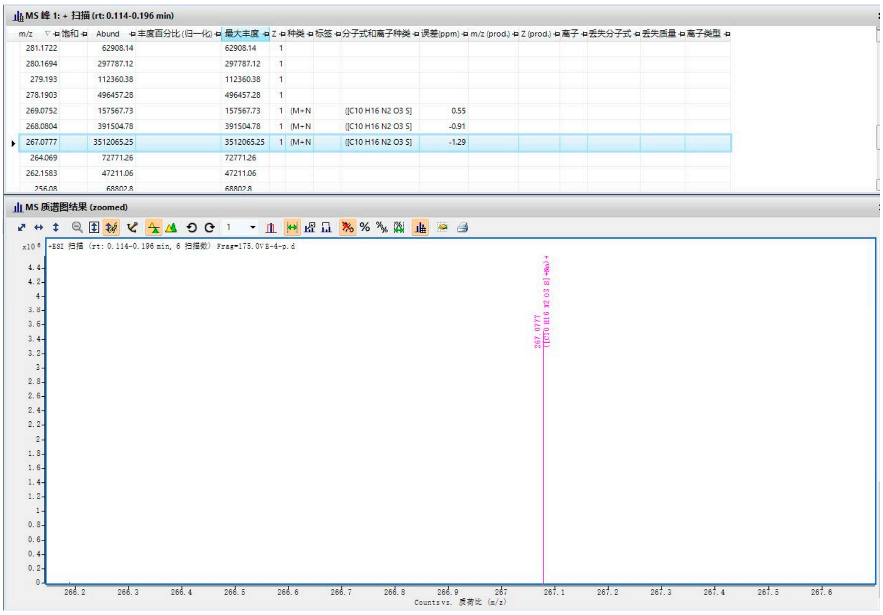

Fig. S69 HR-ESI-MS spectrum of synthetic (+)-**3.**

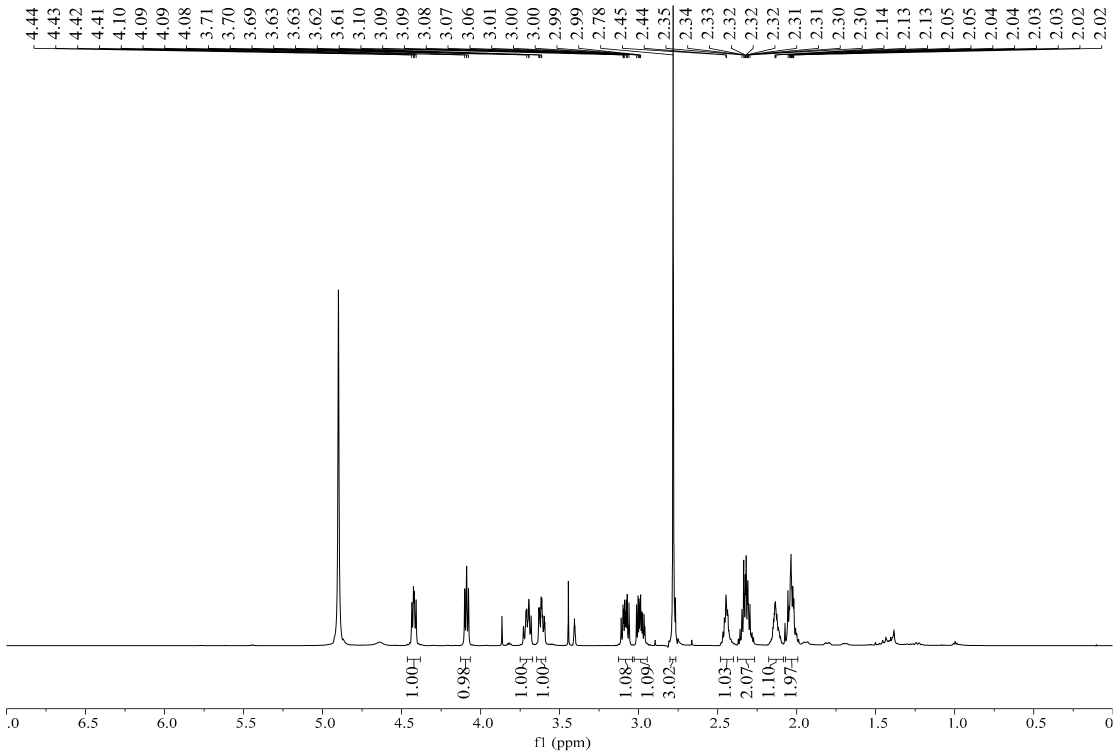

Fig. S70 ^{1}H NMR spectrum (600 MHz, CD_3OD) of synthetic (+)-**3.**

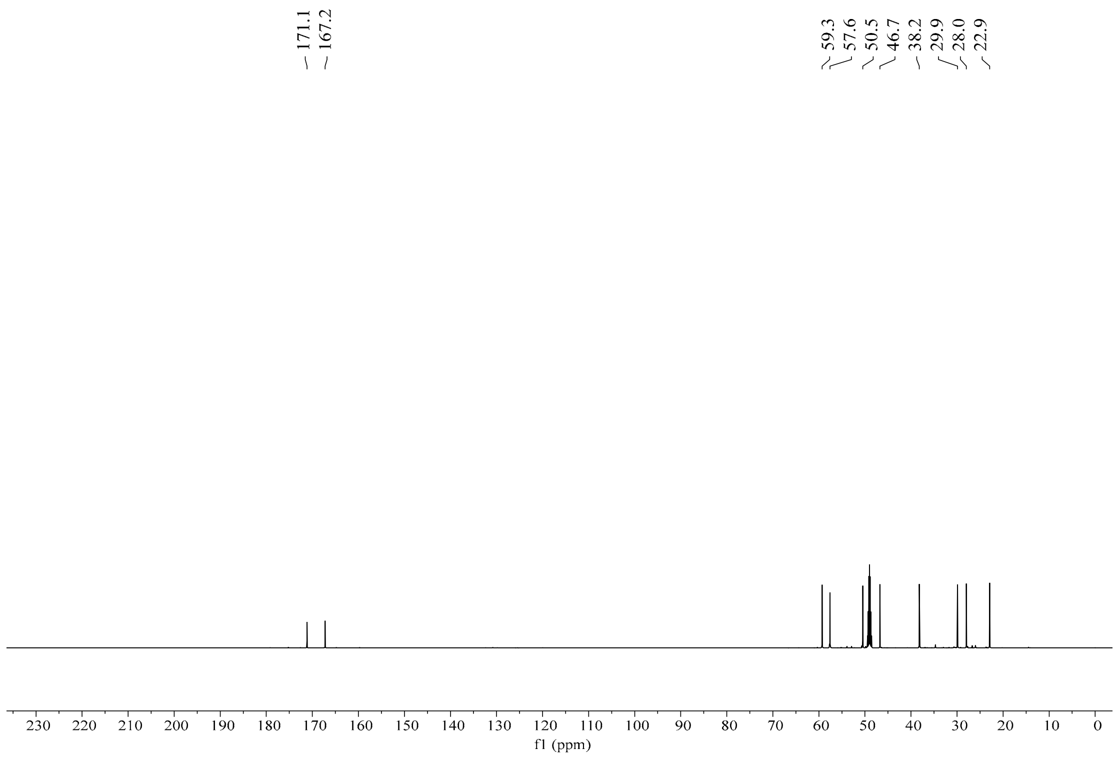

Fig. S71 ^{13}C NMR spectrum (150 MHz, CD_3OD) of synthetic (+)-**3.**

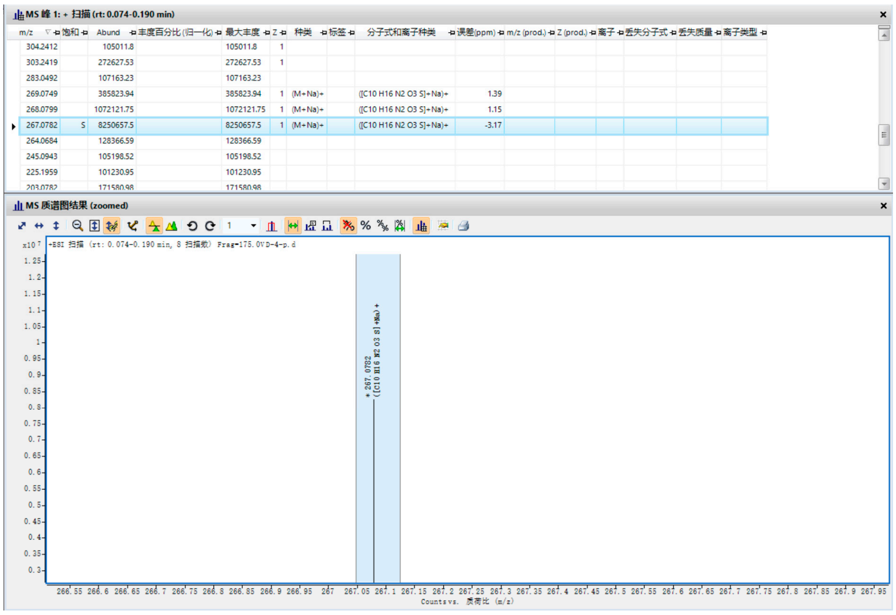

Fig. S72 HR-ESI-MS spectrum of synthetic (-)-**3.**

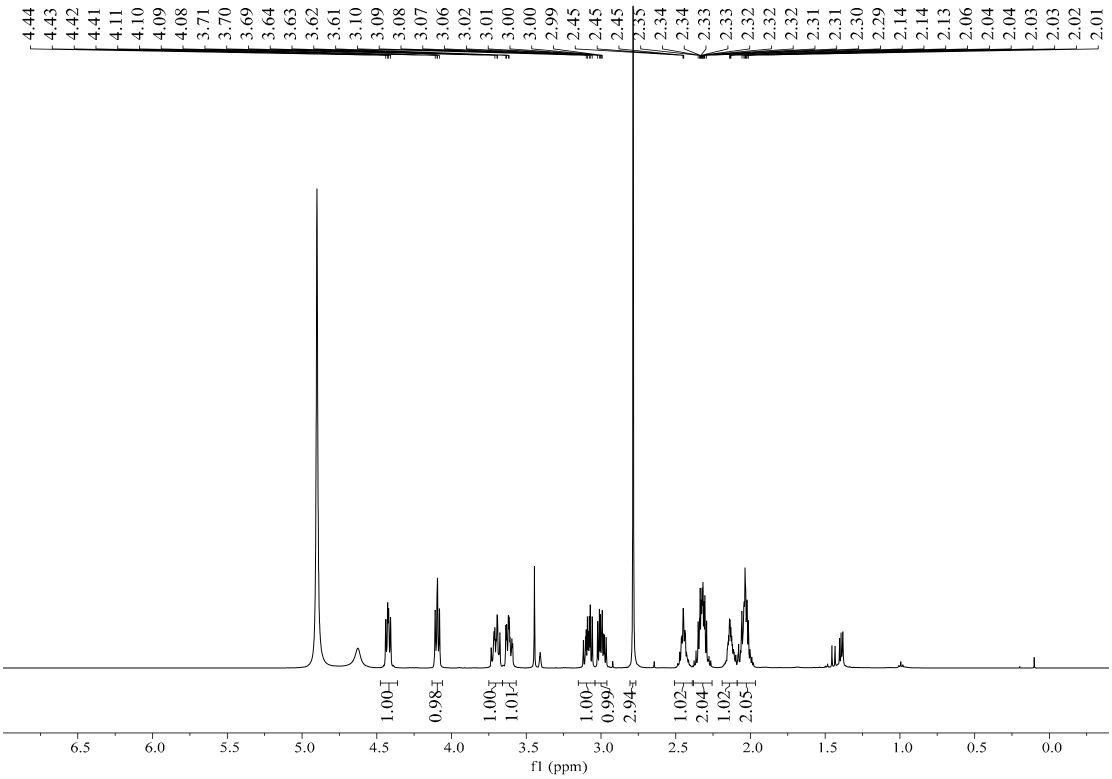

Fig. S73 ^{1}H NMR spectrum (600 MHz, CD_3OD) of synthetic (-)-**3.**

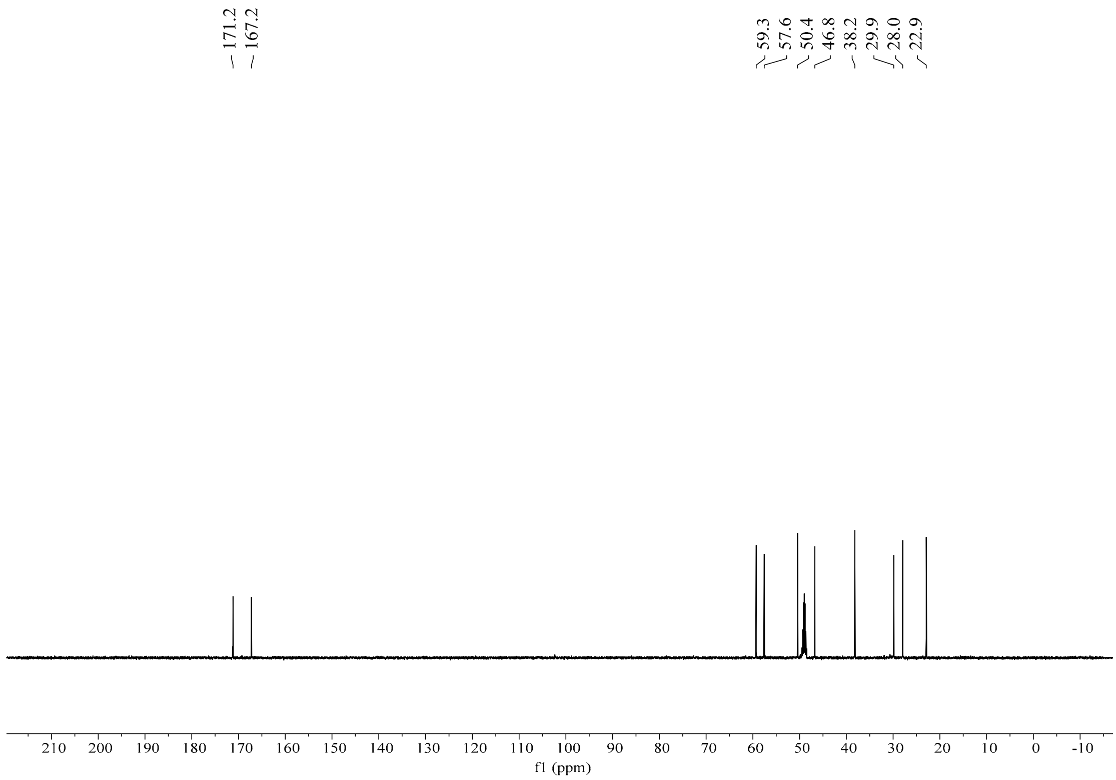

Fig. S74 ^{13}C NMR spectrum (150 MHz, CD_3OD) of synthetic (-)-**3.**

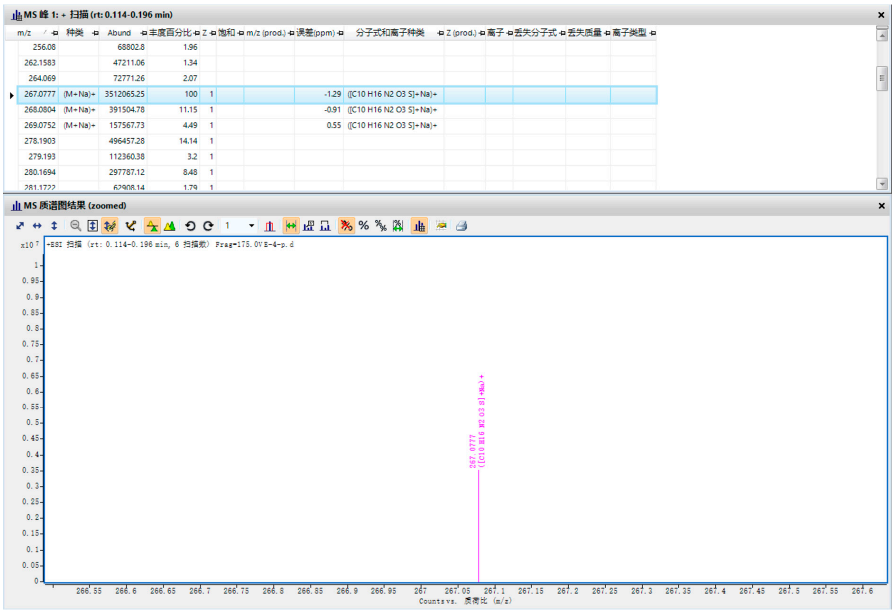

Fig. S75 HR-ESI-MS spectrum of synthetic (+)-**4.**

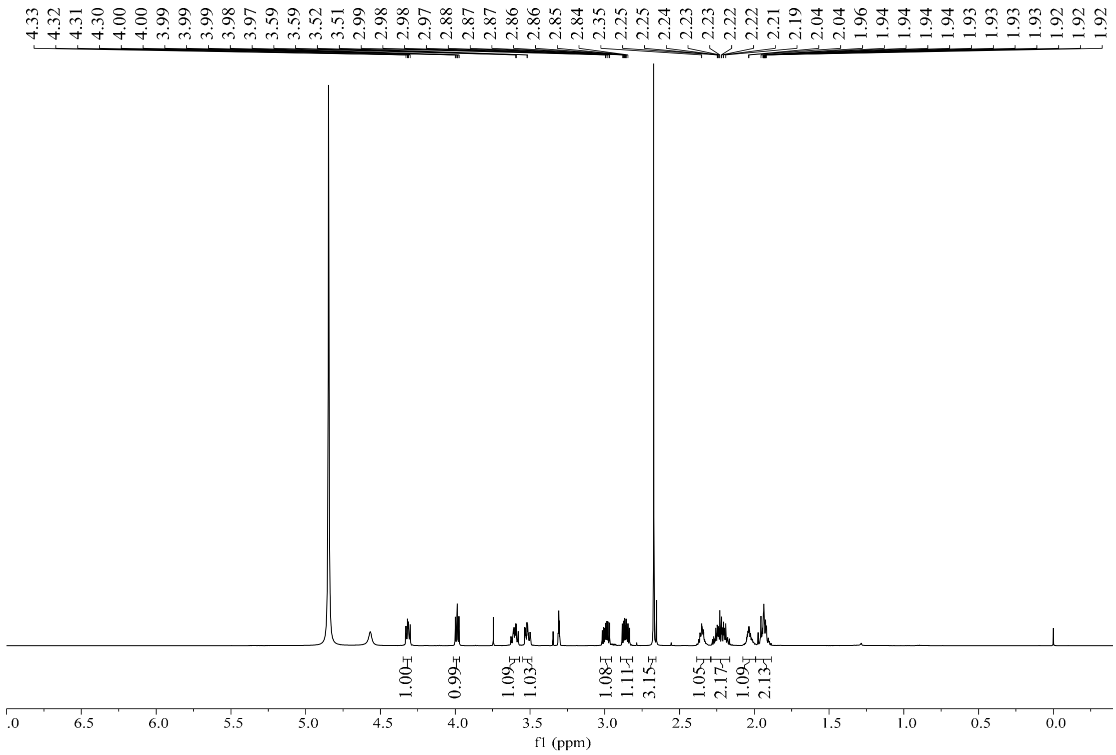

Fig. S76 ^{1}H NMR spectrum (600 MHz, CD_3OD) of synthetic (+)-**4.**

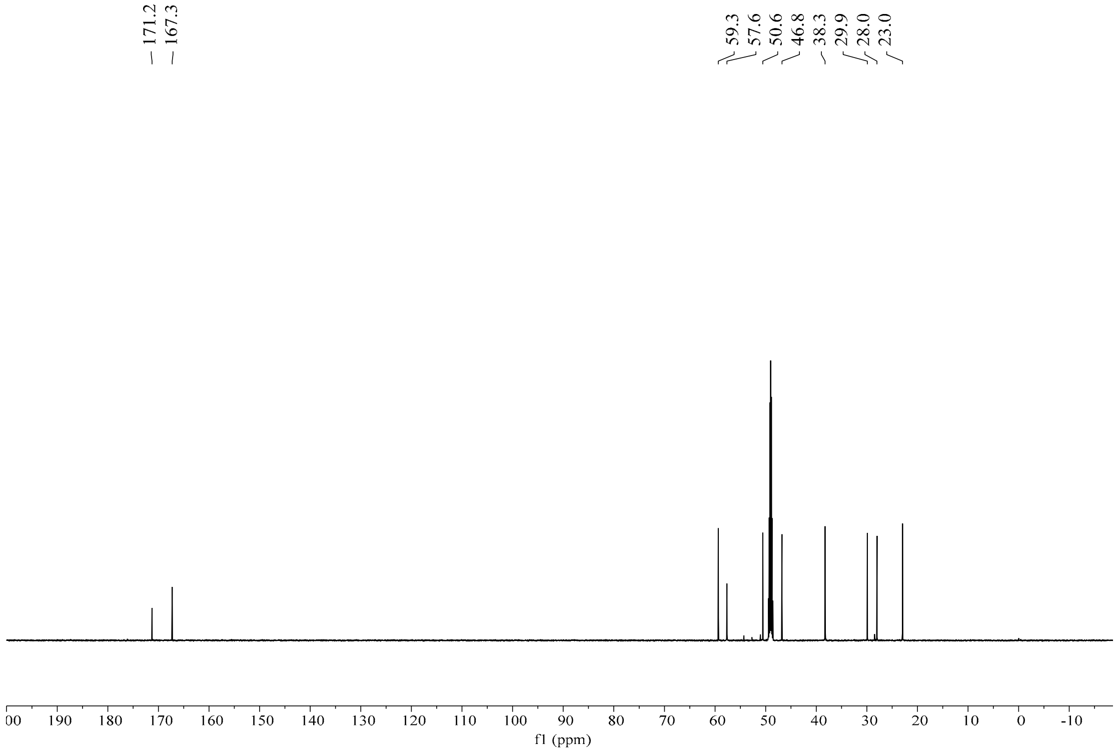

Fig. S77 ^{13}C NMR spectrum (150 MHz, CD_3OD) of synthetic (+)-**4.**

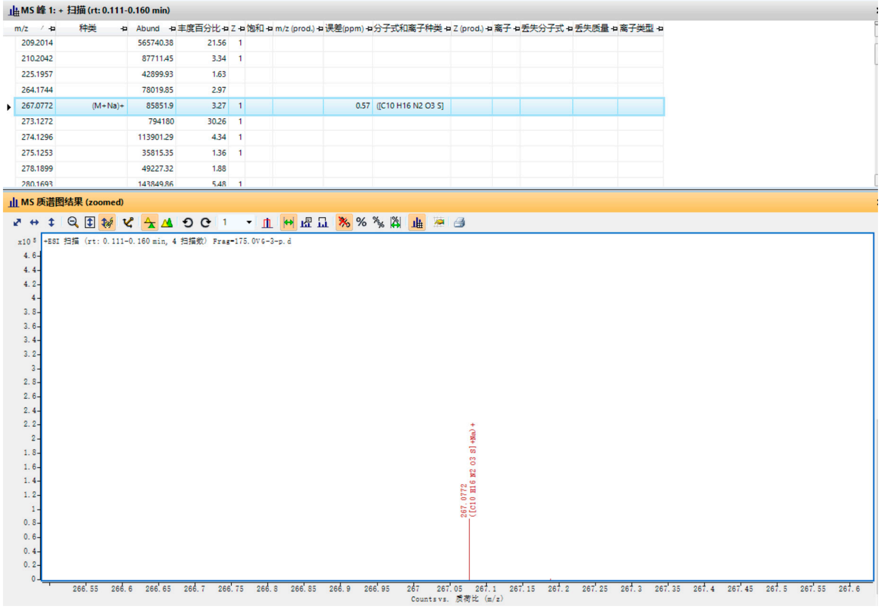

MS 峰 1: + 扫描 (rt: 0.111-0.160 min)

m/z	种类	Abund	丰度百分比	Z	饱和	m/z (prod.)	误差(ppm)	分子式和离子种类	Z (prod.)	离子	丢失分子式	丢失质量	离子类型
209.2014		565740.38	21.56	1									
210.2042		87711.45	3.34	1									
225.1957		42899.93	1.63										
264.1744		78019.85	2.97										
267.0772	(M+Na)+	85851.9	3.27	1			0.57	([C10 H16 N2 O3 S]					
273.1272		794180	30.26	1									
274.1296		113901.29	4.34	1									
275.1253		35815.35	1.36	1									
278.1899		49227.32	1.88										
280.1693		143849.86	5.48	1									

Fig. S78 HR-ESI-MS spectrum of synthetic (-)-**4.**

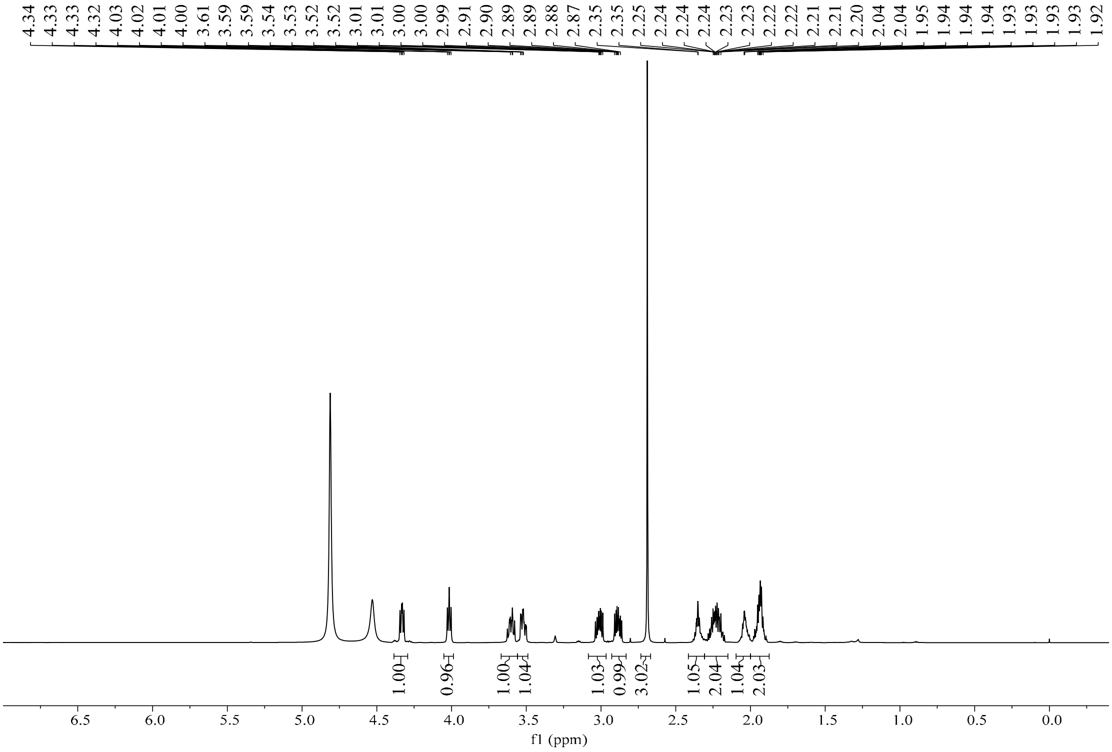

Fig. S79 ^{1}H NMR spectrum (600 MHz, CD_3OD) of synthetic (-)-**4.**

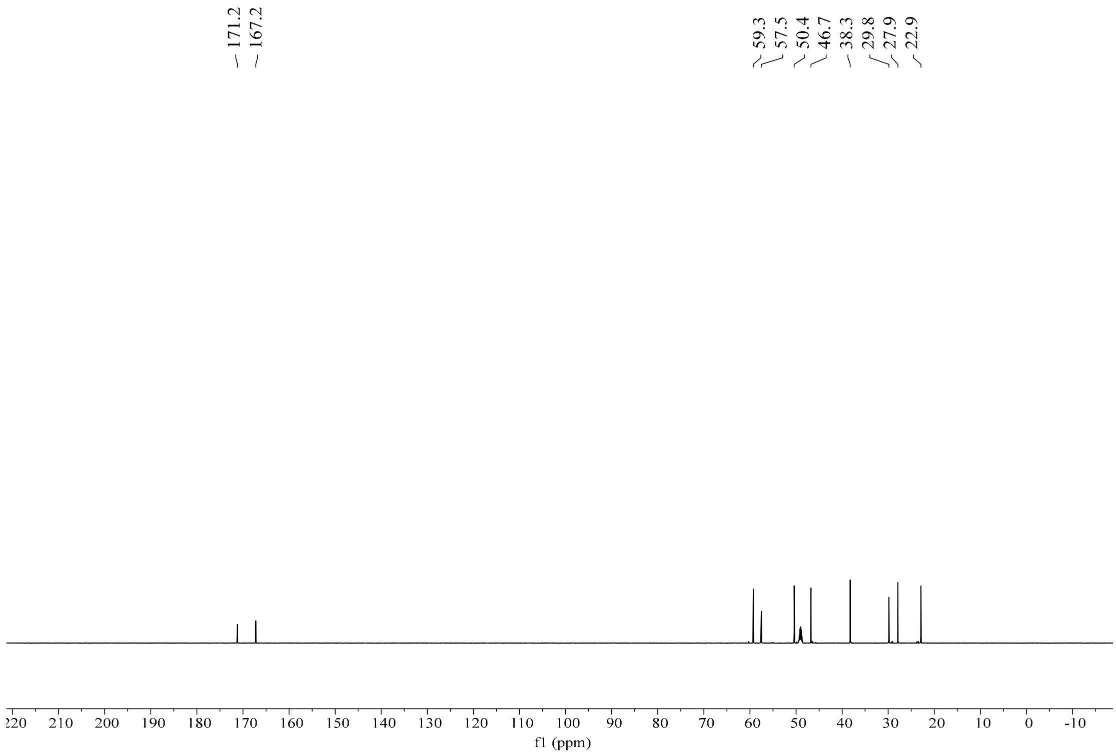

Fig. S80 ^{13}C NMR spectrum (150 MHz, CD_3OD) of synthetic (-)-**4.**
